# Supplementary material for: Multienzyme One‐Pot Cascades Incorporating Methyltransferases for the Strategic Diversification of Tetrahydroisoquinoline Alkaloids
Source: Angew Chem Int Ed Engl. 2021 Jul 16;60(34):18673–9. doi: 10.1002/anie.202104476 (PMC8457072; doi:10.1002/anie.202104476)
Supplement: Supplementary file 1 — Supporting Information [file ANIE-60-18673-s001.pdf]

Angewandte Chemie
Eine Zeitschrift der Gesellschaft Deutscher Chemiker

Supporting Information

Multienzyme One-Pot Cascades Incorporating Methyltransferases for the Strategic Diversification of Tetrahydroisoquinoline Alkaloids

*Fabiana Subrizi, Yu Wang, Benjamin Thair, Daniel Méndez-Sánchez, Rebecca Roddan, Max Cárdenas-Fernández, Jutta Siegrist, Michael Richter, Jennifer N. Andexer, John M. Ward, and Helen C. Hailes**

anie_202104476_sm_miscellaneous_information.pdf

Author Contributions

F.S. Conceptualization: Supporting; Investigation: Lead; Methodology: Lead; Supervision: Supporting; Writing – original draft: Lead
Y.W. Investigation: Supporting; Methodology: Supporting
B.T. Investigation: Supporting; Methodology: Supporting; Writing – original draft: Supporting
D.M. Investigation: Supporting; Methodology: Supporting
R.R. Investigation: Supporting; Methodology: Supporting
M.C. Investigation: Supporting; Methodology: Supporting
J.S. Investigation: Supporting; Methodology: Supporting
M.R. Investigation: Supporting; Methodology: Supporting; Writing – review & editing: Supporting
J.A. Investigation: Supporting; Methodology: Supporting; Writing – review & editing: Supporting
J.W. Conceptualization: Supporting; Investigation: Supporting; Methodology: Supporting
H.C.H. Conceptualization: Lead; Investigation: Supporting; Supervision: Lead; Writing – original draft: Lead; Writing – review & editing: Lead.

Table of Contents

1. Cloning, expression and enzyme preparation **S2**

1.1. Cloning procedures
1.2. Recombinant protein expression in *E. coli* BL21 (DE3)
1.3. Cell lysate preparation
1.4. Enzyme purification

2. General experimental and analytic methods **S5**

2.1. Chemicals, NMR spectroscopy and mass spectroscopy
2.2. Analytical and preparative HPLC

3. Biocatalytic reactions **S5**

3.1. Single step methylation reactions for preliminary screening
3.2. Comparison between pure enzymes and clarified lysates for *Rn*COMT and *Mx*SafC
3.3. Multi-enzyme methylation reactions with the SAM supply system (scale up optimization)
3.4. Preparative scale multi-enzyme cascade reactions with the SAM supply system and product characterisation

4. Analytical HPLC chromatograms **S52**

5. Docking **S54**

6. References **S55**

1. Cloning, expression and enzyme preparation

1.1. Cloning procedures

The cloning procedures of the genes encoding *Ec*MAT (*E. coli* methionine adenosyltransferase), *Rn*COMT (*Rattus norvegicus* catechol-O methyltransferase), *Ec*MTAN (*E. coli* methylthioadenosine/ *S*-adenosylhomocysteine nucleosidase), SafC from *Myxococcus xanthus* (*Mx*SafC), *Ef*TyrDC (*Enterococcus faecalis* DC32 decarboxylase), and *Cn*TYR (*Candidatus Nitrosopumilus salaria BD31* tyrosinase) have been described previously.[1–4]

Synthetic gene 6OMT_COPJA (NCBI accession number Q9LEL6) was provided by Eurofins Genomics in a plasmid vector pEX-K248, and sub-cloned in our in-house plasmid pET28a-SacB-SapI (containing the C-terminal His6Tag). The plasmid construct was transformed into chemically competent *E. coli* NovaBlue and grown overnight in LB agar with kanamycin 50 µg mL^{-1} at 37 °C. A colony was picked and grown overnight in 10 mL of LB broth with kanamycin 50 µg mL^{-1} at 37 °C and 250 rpm, and the plasmid was extracted using QIAprep Spin Miniprep kit (Qiagen). The plasmid construct was then transformed into expression host *E. coli* BL21(DE3).

Synthetic gene *Cj*NMT (CNMTI - *Coptis japonica*, UniProt accession number Q948P7) was provided by BioCat GmbH (Germany) in a plasmid vector pET28a (containing the N-terminal His6Tag). The plasmid construct was transformed into electro competent *E. coli* BL21(DE3) and grown overnight in LB agar with kanamycin 30 µg mL^{-1} at 37 °C. A colony was picked and grown overnight in 50 mL of LB broth with kanamycin 30 µg mL^{-1} at 37 °C and 150 rpm.

1.2. Recombinant protein expression in *E. coli* BL21 (DE3)

***Cj*-6-OMT (*Coptis japonica* norcoclaurine 6-*O*-methyltransferase) expression:** Selected enzyme glycerol stocks (*E. coli* BL21 (DE3)) were plated out on agar plates supplemented with 50 µg/mL kanamycin. A single colony was then picked to inoculate into 10 mL of TB media supplemented with 50 µg/mL kanamycin and grown at 37 °C and 250 rpm overnight (16 h). 1 mL of the overnight cultures were inoculated into a 500 mL baffled shaking flask containing 100 mL TB media supplemented with 50 µg/mL kanamycin at 37 °C, 250 rpm until an OD_{600} of approximately 0.8. Enzyme expression was initiated by the addition of 0.4 mM IPTG (Sigma-Aldrich) to the culture. Cells were further cultivated at 16 °C, 200 rpm for 48 h. The cells were then harvested by centrifugation at 10000 rpm for 15 min. The cell pellets were stored at -20 °C.

***Cj*CNMT (*Coptis japonica* coclaurine *N*-methyltransferase) expression:** For gene expression in 2 L of LB broth with kanamycin 30 µg mL^{-1} at 37 °C and 150 rpm in baffled flasks induction was carried out by adding 0.2 mM IPTG (final concentration) at an OD_{600} of 0.5. Bacterial cultures were further incubated overnight at 20 °C and 150 rpm.

***Ec*MAT, *Ec*MTAN, *Rn*COMT, and *Mx*SafC expression:** Selected enzyme glycerol stocks (*E. coli* BL21 (DE3)) were plated out on agar plates supplemented with 50 µg/mL kanamycin. A single colony was then picked to inoculate into 10 mL of LB media supplemented with 50 µg/mL kanamycin and grown at 37 °C and 250 rpm overnight (16 h). 1 mL of the overnight cultures were inoculated into a 500 mL baffled shaking flask containing 100 mL LB media supplemented with 50 µg/mL kanamycin at 37 °C, 250 rpm until an OD_{600} of approximately 0.6. Enzyme expression was initiated by the addition of 0.25 mM IPTG (Sigma-Aldrich) to the culture. Cells were further cultivated at 20 °C, 200 rpm for 16-24 h. The cells were then harvested by centrifugation at 10000 rpm for 15 min. The cell pellets were stored at -20 °C.

***Tf*NCS, *Cv*-TAm, decarboxylase, and tyrosinase expression** have been described previously.[4]

1.3. Cell lysate preparation

Cells pellets were resuspended in 50 mM HEPES buffer (pH 7.5) at a 1:25 volume ratio (1 mL of the resuspension buffer per 25 mL of cell suspension) and disrupted by 10 cycles of sonication on ice (10 s on plus 10 s off, 12 Watts output) equipped with a Process Timer. In the case of *Cv*-TAm the lysis buffer was supplemented with 1 mM PLP. Lysed cells were centrifuged at 4 °C, 10000 rpm for 15 min. The supernatant protein concentration was measured following the standard Bradford procedure. The samples were duplicated and the average OD_{595} were used for cell lysate concentration calculations. To check the protein expression, the supernatant was examined using an SDS gel (Figure S1).

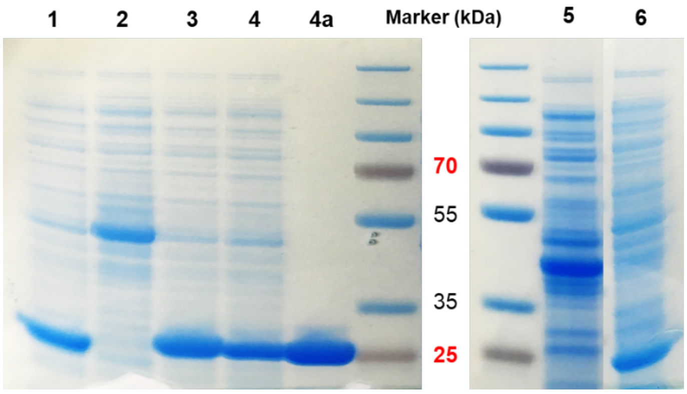

Figure S1. SDS-PAGE analysis of clarified lysates (1-6) and purified proteins (4a). For each protein, 10-25 µg was applied; 1 – *Rn*COMT (25 kDa), 2 – *Ec*MAT (44 kDa), 3 – *Ec*MTAN (24 kDa), 4 – *Mx*SafC (25 kDa), 4a – pure *Mx*SafC (25 kDa), 5 – *Cj*-6-OMT (39 kDa), 6 – *Tf*NCS (25 kDa), the calculated molecular weight inside the parentheses includes the His6-tag.

1.4. Enzyme purification

***Rn*COMT, *Mx*SafC, and *Ec*MTAN purification:** Cell pellets were lysed as described above. The supernatant was collected by centrifugation at 4 °C, 10000 rpm for 15 min, then filtered through a 0.2 µm cellulose acetate springe filter. Enzymes were purified *via* Ni-NTA affinity chromatography. After stepwise washing with 2 mL fractions of purification buffer containing 0, 10, 20, 50, 100 and 150 mM imidazole, enzymes were eluted from the column using fractions of 2 mL purification buffer containing 200, 250, and 2 x 500 mM imidazole. Finally, proteins were desalted on PD-10 columns according to the protocol of the manufacturer (GE Healthcare, UK). The success of the purification was controlled via SDS PAGE analysis (Figure S2).

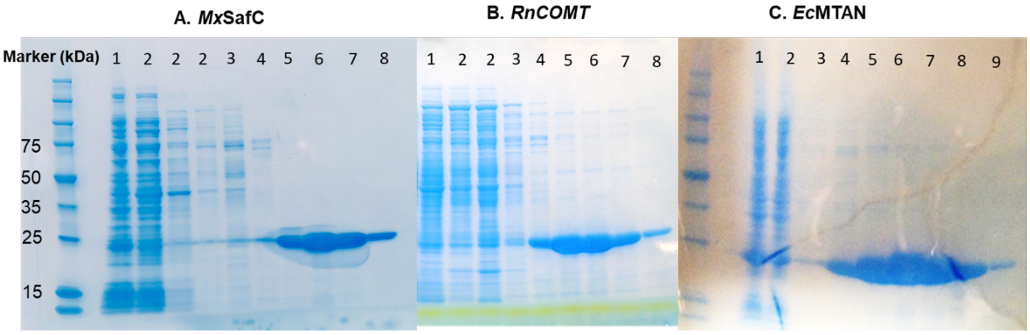

Figure S2. SDS-PAGE analysis of purified protein A – *Mx*SafC (25 kDa), B – *Rn*COMT (25 kDa), C – *Ec*MTAN (24 kDa), 4 – *Tf*NCS (25 kDa); (1 - crude extract, 2 - flow through, wash/ elution steps with buffer containing: 3 - 100 mM imidazole, 4 - 150 mM imidazole, 5 - 200 mM imidazole, 6 - 250 mM imidazole, 7/8- 500 mM imidazole). The calculated molecular weight inside the parentheses always includes the His6-tag.

***Ec*MAT and *Tf*NCS (Δ29*Tf*NCS) purification:** Cell pellets were lysed as described above. The supernatant was collected by centrifugation at 4 °C, 10000 rpm for 15 min, then filtered through a 0.2 µm cellulose acetate springe filter. An empty cartridge charged with Ni-NTA (8 mL) was washed with 40 mL of MilliQ water, followed by 40 mL of binding buffer (50 mM HEPES, 20 mM imidazole (Sigma-Aldrich), 100 mM NaCl, pH 7.5). The filtered supernatant was then passed through the Ni-NTA column, and the column was washed with wash buffer (40 mL, 50 mM HEPES, 40 mM imidazole, pH 7.5) to remove some background protein. The bound protein was then eluted with elution buffer (50 mM HEPES, 500 mM imidazole, 100 mM NaCl, pH 7.5) until all the protein was collected. The eluent containing pure enzyme was concentrated using a vivaspin (10000 MW) at 4 °C, 8000 rpm for 5 min until 2.5 mL eluent remained. Then the concentrated eluent was desalted into 3.5 mL of 50 mM HEPES (pH 7.5), using a SephadexTM G-25 in PD-10 column (GE Healthcare lifesciences). The concentration of the pure protein was measured by OD_{280} using a Nanodrop. The protein was aliquoted and stored at -20 °C in 10% glycerol. To check the protein purity, the expression supernatant, and pure protein were examined using an SDS gel (Figure S3).

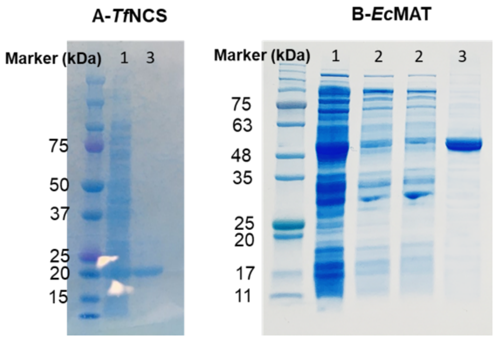

Figure S3. SDS-PAGE analysis of purified protein (1 - crude extract, elution steps with buffer containing: 2 - 40 mM imidazole, 3 - 500 mM imidazole). A –EcMAT (44 kDa), B – TfNCS (25 kDa), the calculated molecular weight inside the parentheses always includes the His6-tag.

***Cj*CNMT purification:** The purification of *Cj*CNMT was carried out *via* an Äkta Purifier (GE Healthcare) according to the following protocol. Lysis buffer (40 mM Tris HCl, 100 mM NaCl, 20 mM imidazole, pH 8.0), elution buffer (40 mM Tris HCl, 100 mM NaCl, 500 mM imidazole; pH 8.0) and desalting buffer (100 mM potassium phosphate, pH 7.5) were used. The cell pellet was stored at -20 °C. For lysis it was thawed and resuspended in lysis buffer using 2-5 mL per gram of wet weight. Then lysozyme and DNaseI (each 0.1 mg/mL) were added and incubated on ice for 30 min. Cell disruption was achieved by using a French Press (1,81 kbar at 5 °C). The lysate was cleared by centrifugation at 10,000 x g for 30 min at 4°C and subsequently filtered through a 0.2 µm filter membrane. For purification through IMAC a Ni-NTA column was equilibrated with lysis buffer. Then the cleared sample was loaded onto the column with a volumetric flow rate of 2.0 mL/min. The unbound or unspecific bound protein was washed away with lysis buffer until the absorption at 280 nm reached the baseline. Further, the His_6-tagged protein was eluted using a step gradient of lysis buffer and elution buffer (first step: 5% (v/v) elution buffer, second step: 60% (v/v) elution buffer, third step: 100% elution buffer). The target enzyme was found in the fraction using 60% elution buffer. A sample of each fraction was collected and monitored *via* SDS-PAGE analysis. The fraction containing the enriched protein were desalted using PD10 columns. Freeze-drying was carried out and the protein was stored and -20 °C.

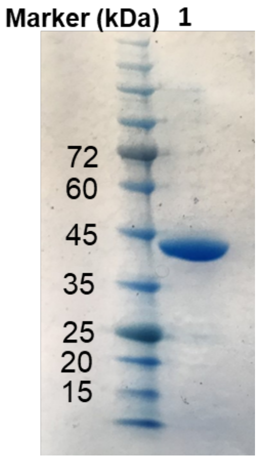

Figure S4. SDS-PAGE analysis of purified protein; 1- *Cj*CNMT (41 kDa) sample after desalting, the calculated molecular weight inside the parentheses always includes the His6-tag.

2. General experimental and analytic methods

2.1. Chemicals, NMR spectroscopy and mass spectrometry

The solvents and chemicals were purchased from Sigma-Aldrich (Saint Louis, MO, USA) or Alfa Aesar (Thermo Fisher Scientific Inc., MA, USA). All chemicals were purchased in the highest purity available and were used as supplied. Adenosine 5′-triphosphate (ATP) was purchased as a disodium salt hydrate Grade II, ≥98.5% (HPLC), crystalline, from Sigma-Aldrich. *S*-(5'-Adenosyl)-L-methionine (SAM) was purchased as the *p*-toluenesulphonate from Sigma-Aldrich. ^{1}H and ^{13}C NMR spectra were recorded respectively at 600 MHz and 150 MHz on a Bruker Avance 600 spectrometer or at 700 MHz and 176 MHz on a Bruker Avance 700 spectrometer in the stated solvent. Chemical shifts (in ppm) are quoted relative to tetramethylsilane and referenced to residual protonated solvent. Coupling constants (*J*) are measured in Hertz (Hz) and multiplicities for ^{1}H NMR coupling are shown as s (singlet), d (doublet), t (triplet), and m (multiplet). Mass spectra were obtained using either a VG70-SE or MAT 900XP spectrometer at the Department of Chemistry, University College London. All optical rotations were measured on a Bellingham and Stadley ADP 430 polarimeter with a path length of 0.5 cm.

2.2. Analytical and preparative HPLC

Achiral analytical HPLC methods were performed with a HPLC system Agilent 1260 Infinity II system equipped with a DAD detector.

Method A1: Analytical reverse-phase (RP) HPLC was conducted with a ACE 5-C18 300 column (4 µm, 4.6 mm × 150 mm) at 30 °C [5% B for 2 min, gradient of 5% to 70% B up to 10 min, 70% B to 100% B over 0.1 min, 100% B for 1.9 min, 100% B to 5% B over 0.1 min, 5% B for 2.4 min (A = H_2O with 0.1% TFA; B = acetonitrile) flow rate = 1 mL/min; A_{280} if not otherwise specified].

Method A2: Analytical reverse-phase (RP) HPLC was conducted with a ACE 5-C18 300 column (4 µm, 4.6 mm × 150 mm) at 30 °C [10% B for 1 min, gradient of 10% to 70% B up to 5 min, 70% B to 100% B over 0.1 min, 100% B for 0.4 min, 100% B to 10% B over 0.1 min, 5% B for 3.4 min (A = H_2O with 0.1% TFA; B = acetonitrile) flow rate = 1 mL/min; A_{280} if not otherwise specified].

Preprative HPLC methods were developed with an Angilent 1260 InfinityTM HPLC System, with a 1260 InfinityTM Preparative Pump, a 1260 InfinityTM Preparative-scale Fraction Collector, a 1260 InfinityTM Multiple Wavelength Detector and a 1260 InfinityTM Preparative Autosampler.

Method B: The separation of compounds (*S*)-6-OMe-**1**, (*S*)-2-NMe-6-OMe-**1**, (*S*)-6-OMe-**2**, (*S*)-6,7-$(OMe)_2$-**2**, (*S*)-6,3'-$(OMe)_2$-**3**, (*S*)-6,4'-$(OMe)_2$-**3**, (*S*)-2-NMe-6,3'-$(OMe)_2$-**3**, (*S*)-6-OMe-**5**, (*S*)-6-OMe-**6**, 6-OMe-**9**, 7-OMe-**9**, 6-OMe-**10**, 7-OMe-**10**, and (*S*)-6-OMe-**11** was achieved with a SupelcoTM Discovery BIO wide pore (C18, 10 µm, 2.12 cm x 25 cm) preparative column at 25 °C [5% B for 3 min, gradient of 5% to 70% B up to 20 min, 70% B to 100% B over 0.1 min, 100% B for 1.9 min, 100% B to 5% B over 1 min, 5% B for 5 min (A = H_2O with 0.1% TFA; B = acetonitrile with 0.1% TFA) flow rate = 8 mL/min; products were identified via UV absorbances at 214 nm and 280 nm]. The injection volume was 500-900 µL.

Chiral analytical HPLC methods were performed with a HPLC system Agilent 1260 Infinity II system equipped with a DAD detector.

Method C1: The chiral separation of compound (*rac*)-**1** and (*S*)-**1**, (*S*)-6-OMe-**1**, (*rac*)-**2** and (*S*)-**2**, (S)-6-OMe-**2**, (*rac*)-**5** and (*S*)-**5**, (*S*)-6-OMe-**5**, (*S*)-6-OMe-**6**, (*S*)-6-OMe-**11** were achieved with an Supelco Astec Chirobiotic$_{TM}$ T column (25 cm × 4.6 mm) and a flow speed of 1 mL/min at 30 °C. The injection volume was 10 µL. Products were measured via UV absorbance at 230 nm. Methanol (0.2% AcOH, 0.1% TEA) was used as a mobile phase over 40 min.

Method C2: The chiral separation of compound (*rac*)-**3** and *(S)*-**3** were achieved with a Supelco Astec Chirobiotic$_{TM}$ T column (25 cm × 4.6 mm) and a flow speed of 0.2 mL/min at 30 °C. The injection volumes were 5 µL. Compounds were detected by UV absorbance at 230 nm. An isocratic mobile phase 20 mM NH_4OAc pH 4:MeOH (70:30) was used over 120 min.

Method C3: The chiral separation of compound (*rac*)-**11** and (*S*)-**11** were achieved with an Supelco Astec Chirobiotic$_{TM}$ T2 column (25 cm × 4.6 mm) and a flow speed of 1 mL/min at 30 °C. The injection volume was 5 µL. Products were detected via UV absorbance at 230 nm. Methanol (0.2% AcOH, 0.1% TEA) was used as a mobile phase over 40 min.

Method C4: The chiral separation of compound (*rac*)-**6** and (*S*)-**6** were achieved with a Diacel Chiralpak AD-H column (25 cm × 4.6 mm, 5 µM) with an isocratic *n*-hexane:ethanol:diethylamine (80:20:0.01) mobile phase at 1 mL min^{-1}. Products were detected via UV absorbance at 280 nm The column temperature used was 30 °C.

3. Biocatalytic reactions

3.1. Single step methylation reactions for preliminary screening

The assay was conducted in a 96-well plate. Each well contained the reaction mixture consisting of 50 mM HEPES pH 7.5, 20 mM $MgCl_2$, 0.5 mM substrate (**1-14**), 3 mM SAM, and the specific methyltrasferase *Rn*COMT, *Mx*SafC or *Cj*-6-OMT as clarified lysate (10% v/v) in a total volume of 100 µL. The assay was performed at 37 °C, 750 rpm for 90 min unless otherwise specified. After this time, the reaction was quenched with 100 µL of MeOH. All the experiments were performed in duplicate and the reaction mixture was prepared freshly for each experiment. The conversion yields reported refer to depletion of starting material and are calculated using calibration curves for each substrate. When no starting material was left at the end of the reaction a quantitative conversion was reported.

HPLC traces and calibration curves for substrates in Figure 2:

HPLC Method A1 (achiral) (*S*)-**1**

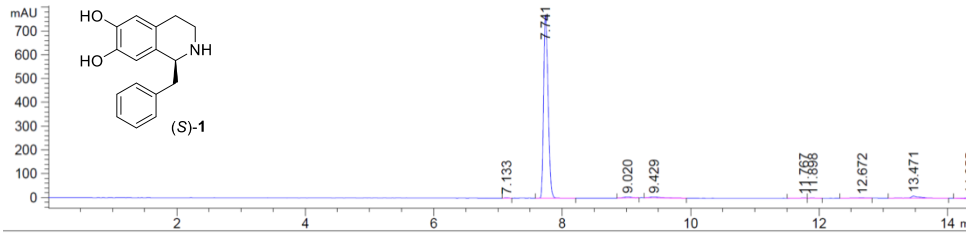

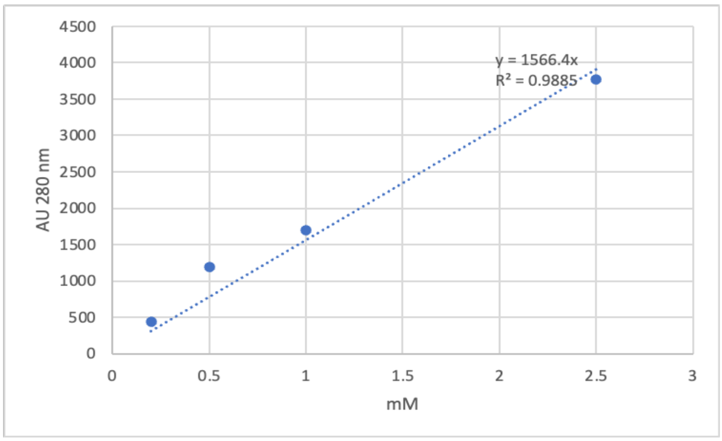

HPLC Method A2 (achiral) (*S*)-**2**

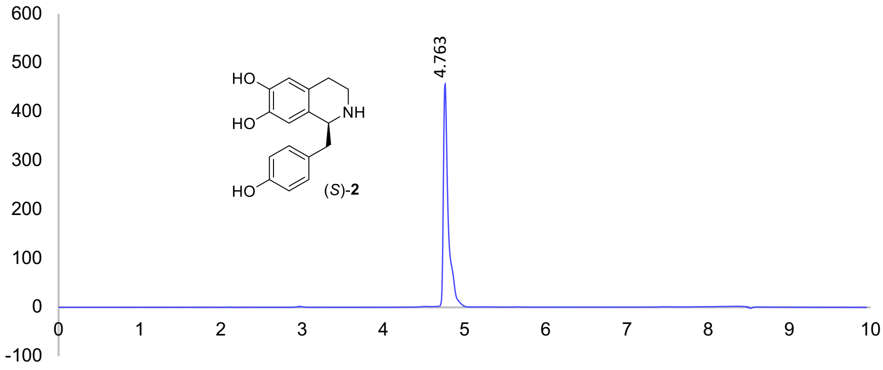

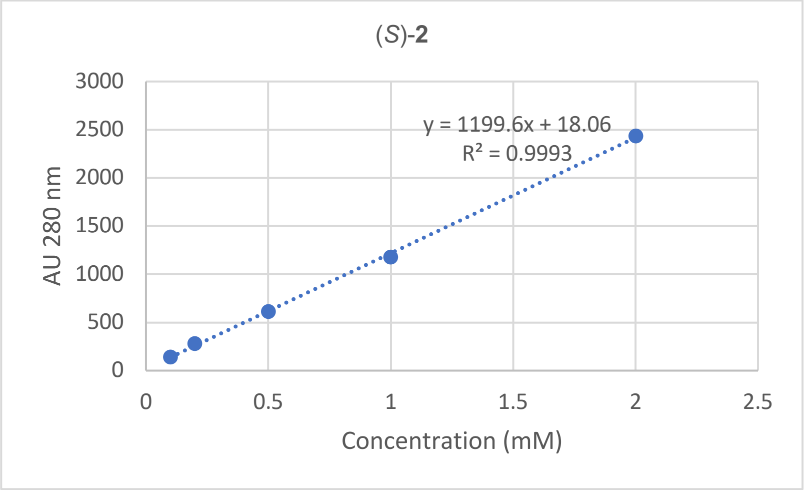

HPLC Method A1 (achiral) (*S*)-**3**

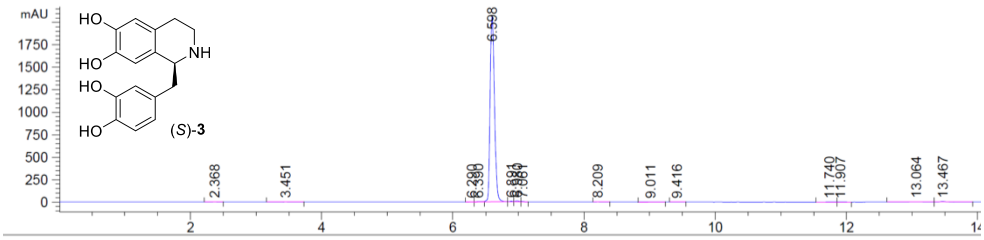

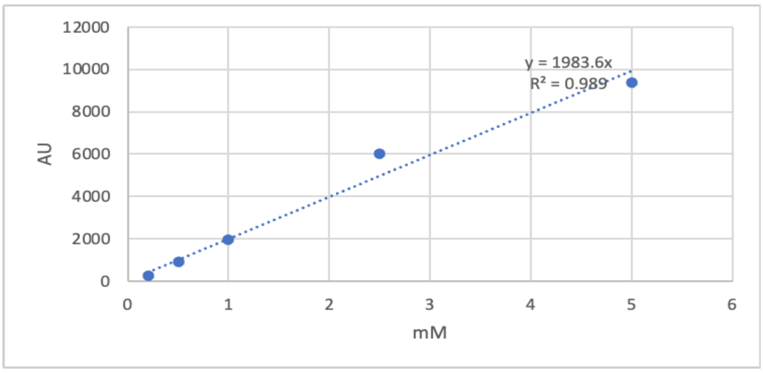

HPLC Method A1 (achiral) (*S*)-**4**

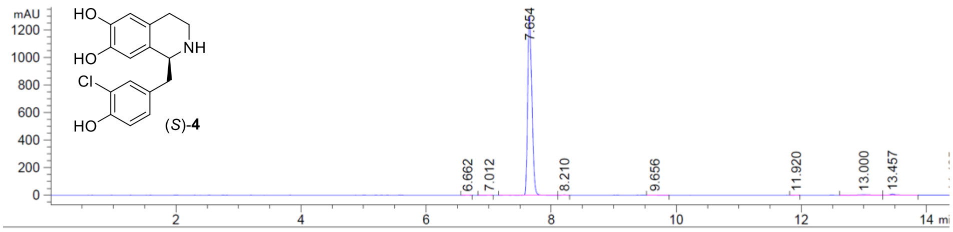

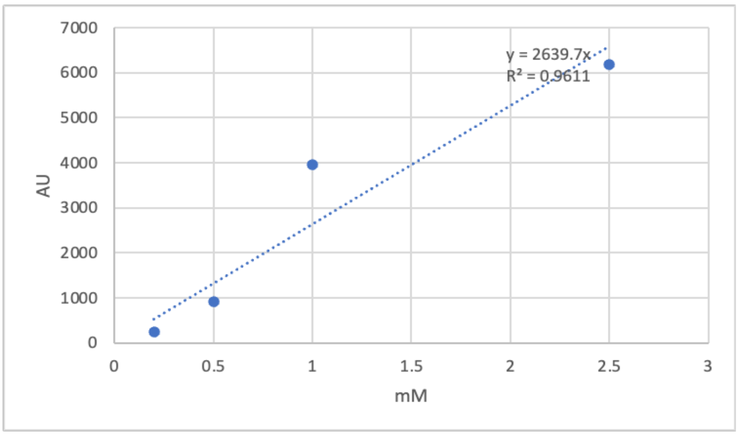

HPLC Method A1 (achiral)

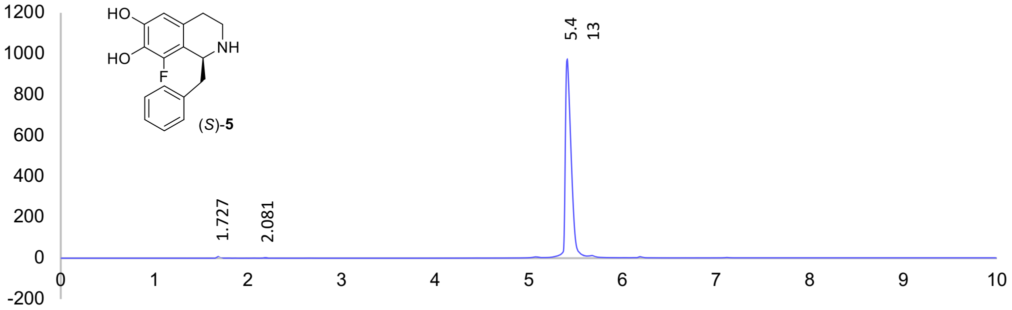

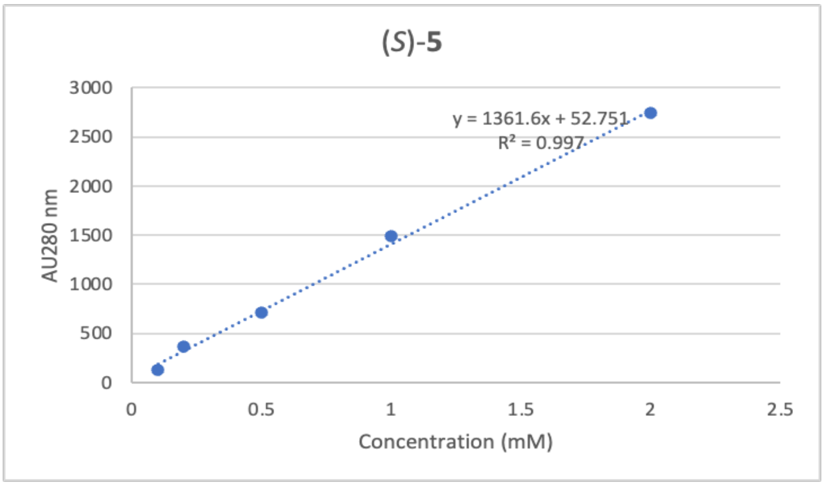

HPLC Method A1 (achiral)

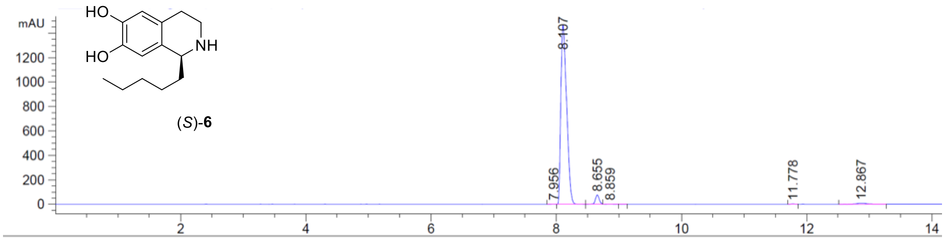

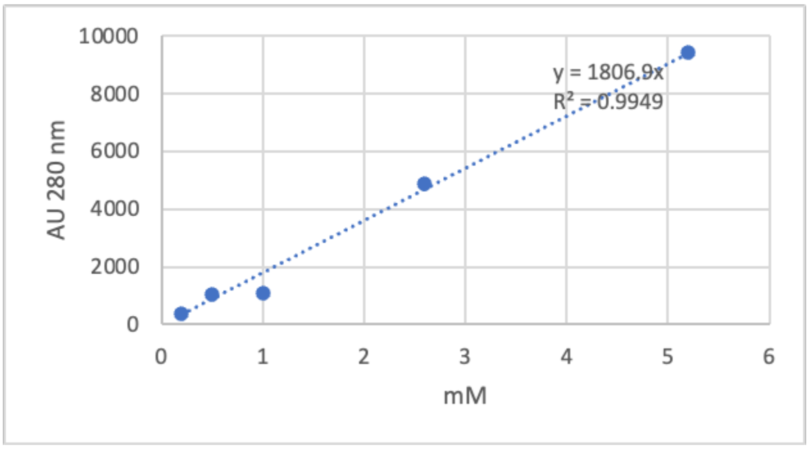

HPLC Method A1 (achiral)

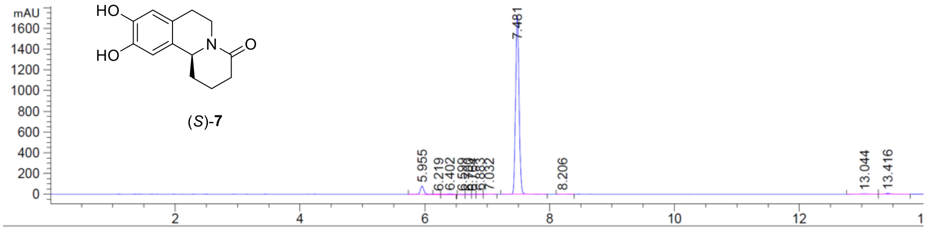

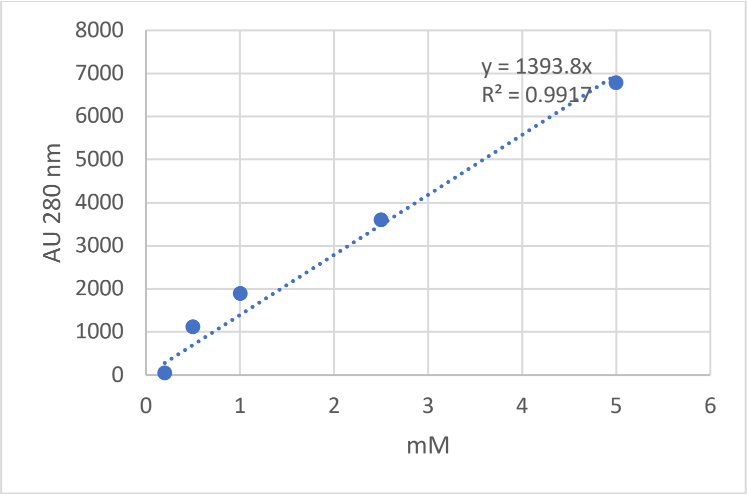

HPLC Method A1 (achiral)

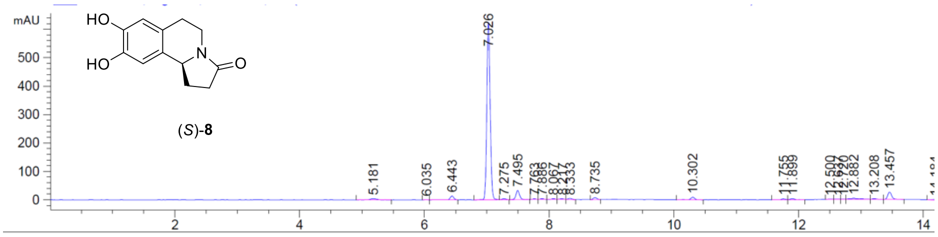

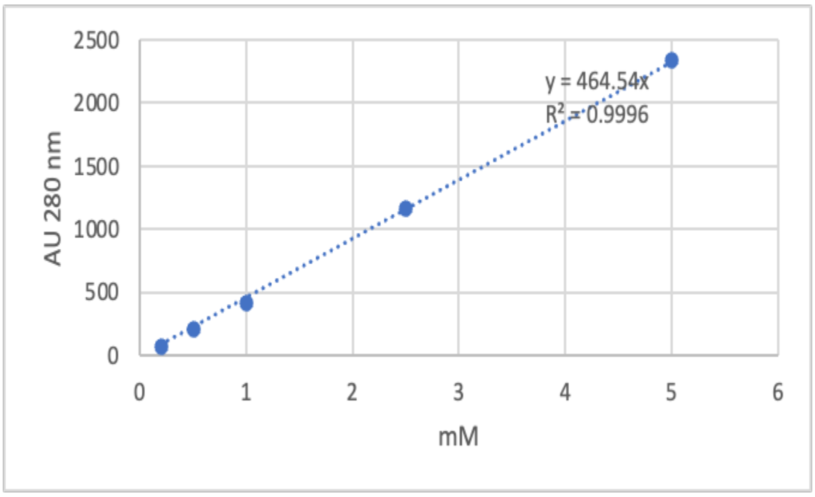

SUPPORTING INFORMATION

HPLC Method A1 (achiral)

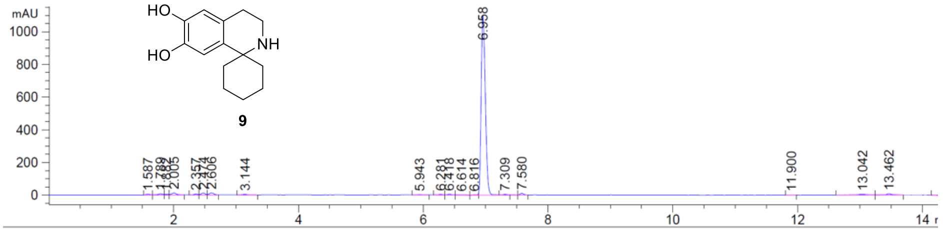

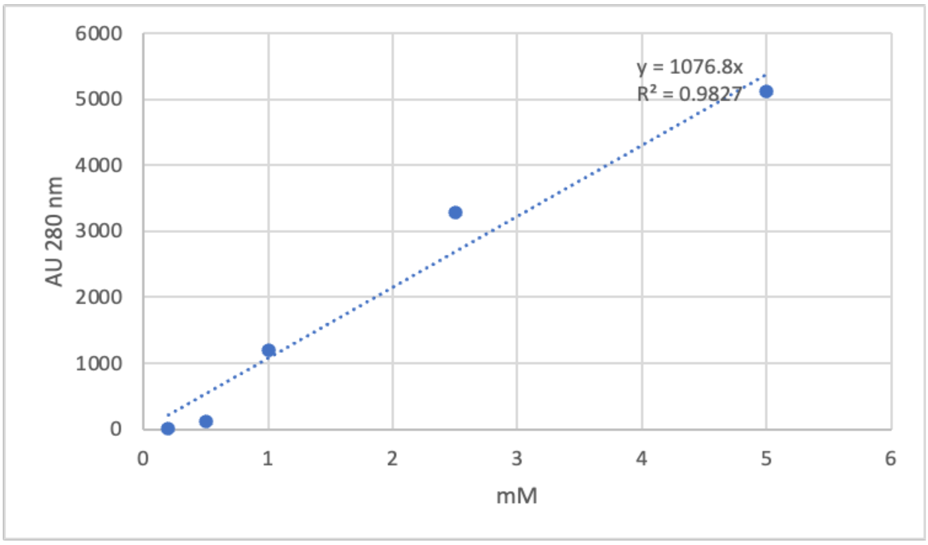

HPLC Method A1 (achiral)

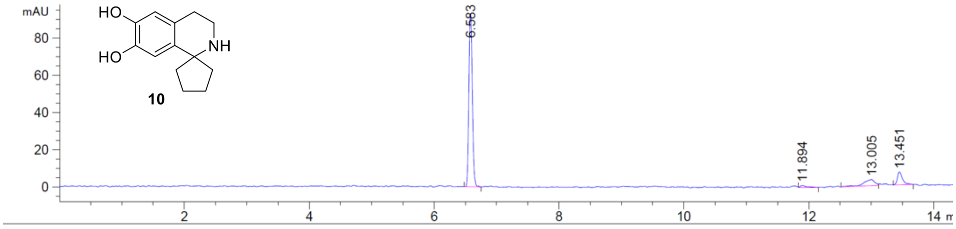

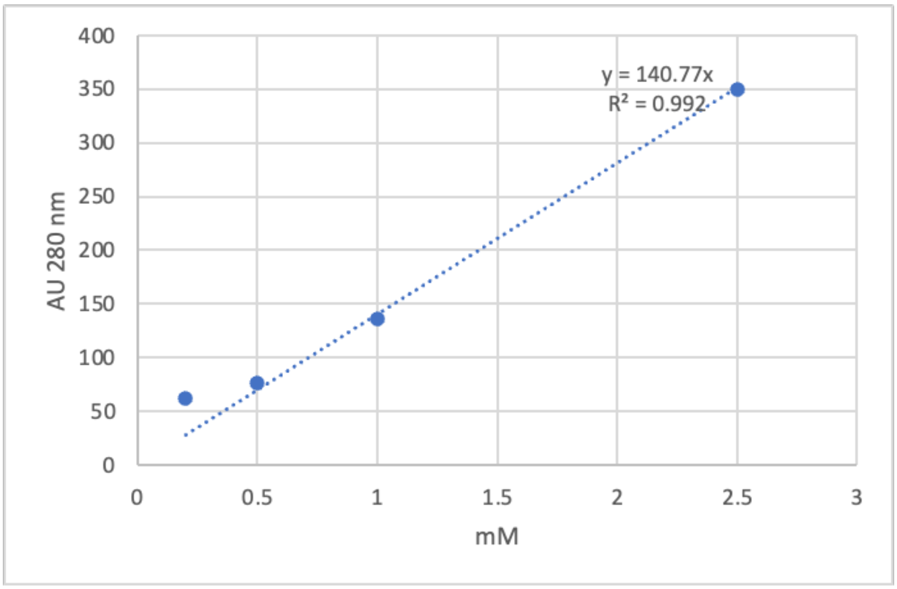

HPLC Method A1 (achiral)

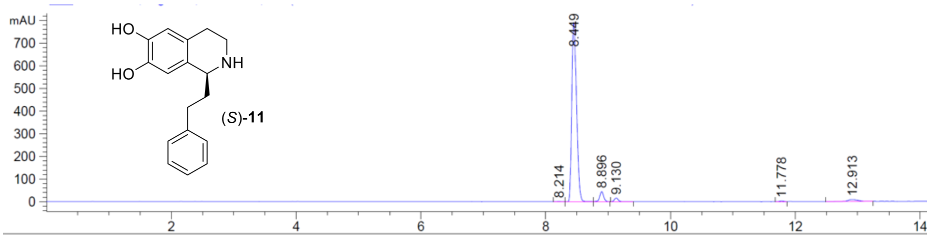

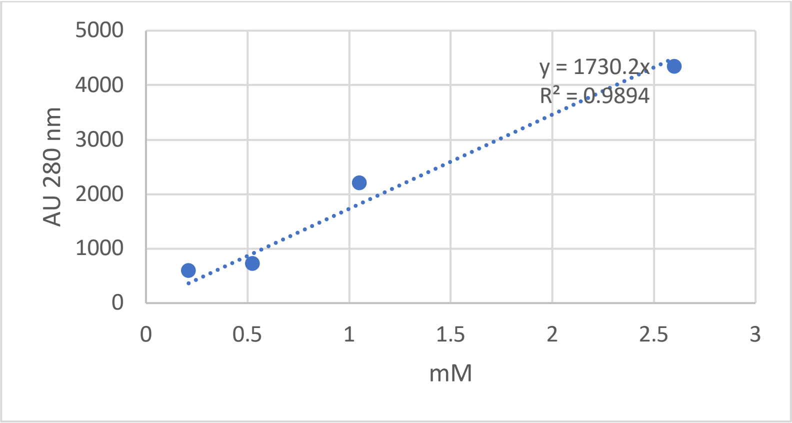

3.2. Comparison between pure enzymes and clarified lysates for *Rn*COMT and *Mx*SafC

The assay was conducted in Eppendorf vials. Each vial contained the reaction mixture consisting of 50 mM HEPES pH 7.5, 20 mM $MgCl_2$, 2.5 mM substrate **1-3**, 2.5 mM SAM, and the specific methyltrasferase *Rn*COMT, *Mx*SafC as clarified lysate (10% v/v) or pure enzyme (0.4 mg/mL) in a total volume of 100 µL. The assay was performed at 37 °C, 450 rpm for 4 h. After this time, the reaction was quenched with 100 µL of MeOH and clarified by centrifugation (Figure S4).

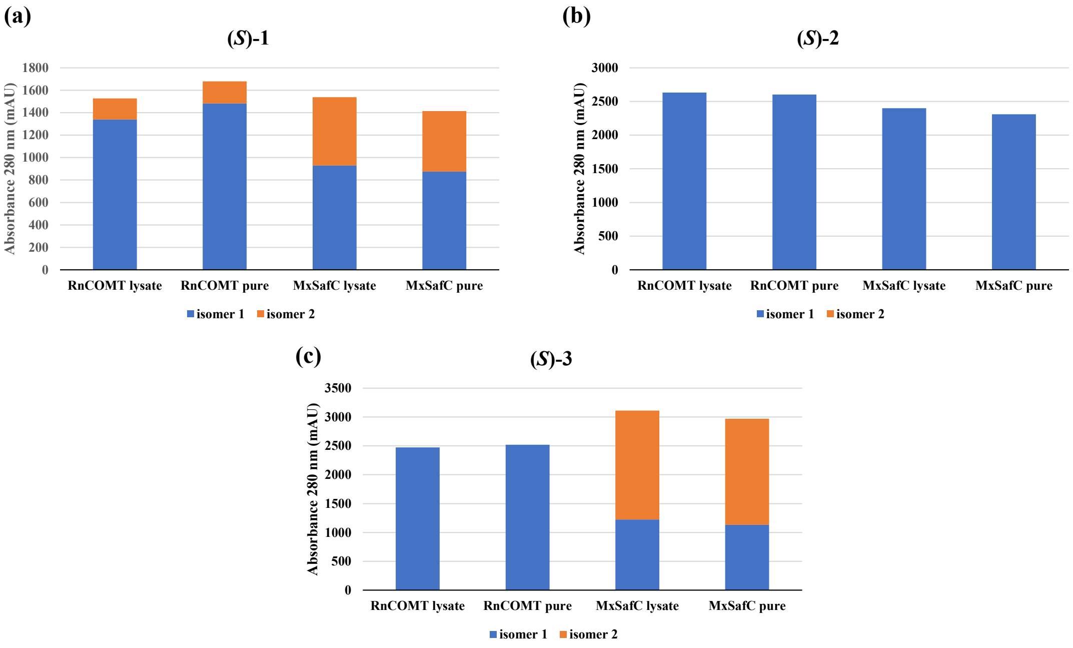

Figure S5. Comparison between pure enzymes and clarified lysates for *Rn*COMT and *Mx*SafC for (a) substrate (*S*)-**1**, (b) substrate (*S*)-**2**, and (c) substrate (*S*)-**3**. Regioselectivity for pure enzyme and clarified lysate can also be compared as peak areas of the two different regioisomers are reported.

3.3. Multi-enzyme methylation reactions with the SAM supply system (scale-up optimization)

Small scale assays were carried out in order to find the optimal conditions for the scale-up reactions. Optimised conditions are reported. The reaction mixture consisted of 50 mM HEPES pH 7.5, 20 mM $MgCl_2$, 200 mM KCl, 5 mM substrate (**1-14**), ATP (1-4 equiv), L-methionine (1-4 equiv), in a total volume of 200 µL. The methylation step was started by adding 10% v/v *Rn*COMT lysate together with *Ec*MAT (0.4 mg/mL) and *Ec*MTAN (0.025 mg/mL). Finally, the pure *Ec*MAT and *Ec*MTAN were replaced by the clarified lysate and 10% v/v *Ec*MAT lysate and 2.5% v/v *Ec*MTAN lysate were added.

The assay was performed at 37 °C, 450 rpm in eppendorf vials for 19 h unless otherwise specified. After this time, the reaction was quenched with 100 µL of MeOH.

3.4. Preparative scale multi-enzyme cascade reactions with the SAM supply system and product characterisation

Four-enzymes cascade to (*S*)-1-benzyl-6-methoxy-1,2,3,4-tetrahydroisoquinolin-7-ol (*S*)-6-OMe-1

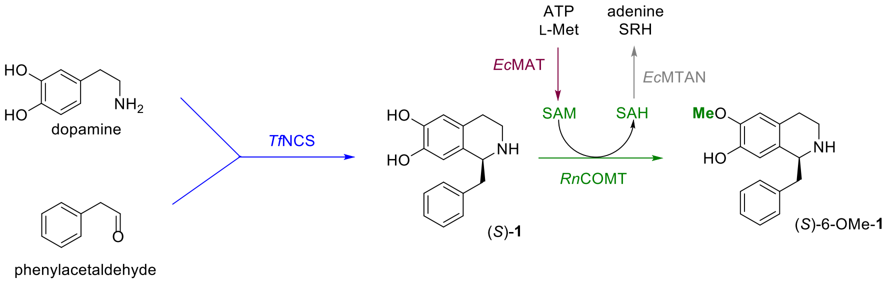

Dopamine hydrochloride (9.45 mg, 0.05 mmol), phenylacetaldehyde (8.4 µl in 0.5 mL of CH_3CN, 0.075 mmol), sodium ascorbate (9.9 mg, 0.05 mmol), and 10% v/v of *Tf*NCS clarified lysate desalted into 50 mM HEPES (pH 7.5), using a SephadexTM G-25 in PD-10 column (GE Healthcare lifesciences), were combined with HEPES (50 mM pH 7.5) to a total volume of 5 mL. The reaction was stirred at 37 °C for 16 h. Once the reaction had completed, the yellow suspension was centrifuged, and the supernatant used for the next step. ATP (55 mg, 0.10 mmol) and L-methionine (15 mg, 0.10 mmol) were combined in HEPES buffer (50 mM) and the pH was adjusted to 7.5. The ATP/L-methionine mixture was added to the supernatant. The methylation step was initiated upon addition of 10% v/v of *Ec*MAT lysate, 2% v/v of *Ec*MTAN lysate, and 10% v/v of *Rn*COMT lysate in a total volume of 10 mL containing 20 mM $MgCl_2$ and 200 mM KCl. The reaction was incubated overnight at 37 °C and 180 rpm. After this time an aliquot was taken to calculate the yield by HPLC against the standard product (see calibration curve below). The reaction was centrifuged, the supernatant was basified with $NaHCO_3$ and extracted with EtOAc (3 x 10 mL). The organic fractions were collected, evaporated under vacuum and the residue purified using preparative HPLC (method B). Fractions containing the desired product were freeze-dried to give (*S*)-**1a** as white powder (TFA salt, 10 mg, 55% yield, 96% yield by HPLC). Trace impurities (5%) were also identified by NMR spectroscopy corresponding to the regioisomeric product methylated at *O*-7. ^{1}H NMR (700 MHz, MeOD) δ 7.40 (t, *J* = 7.6 Hz, 2H, Ar-H), 7.36 – 7.30 (m, 3H, Ar-H), 6.79 (s, 1H, 5-H), 6.63 (s, 1H, 8-H), 4.70 (dd, *J* = 8.8, 5.7 Hz, 1H, 1-H), 3.86 (s, 3H, OMe), 3.53 – 3.45 (m, 2H, 3-*H*H and NHCHC*H*H), 3.30 – 3.26 (m, 1H, 3-H*H*), 3.08 (m, 2H, NHCHCH*H* and 4-*H*H), 3.03 – 2.96 (dt, *J* = 17.1, 6.3 Hz, 1H, 4-H*H*); ^{13}C NMR (176 MHz, MeOD) δ 149.4, 146.9, 136.6, 130.6, 130.3, 128.8, 124.9, 123.6, 114.2, 112.6, 57.7, 56.4, 41.2, 40.9, 25.9; HRMS (ES+) found $[M+H]^+$ 270.1488; $C_{17}H_{20}NO_2$ requires 270.1489; $[\alpha]_D^{25}$ -5.8 (c 0.24, MeOH).

HPLC Method A1 (achiral)

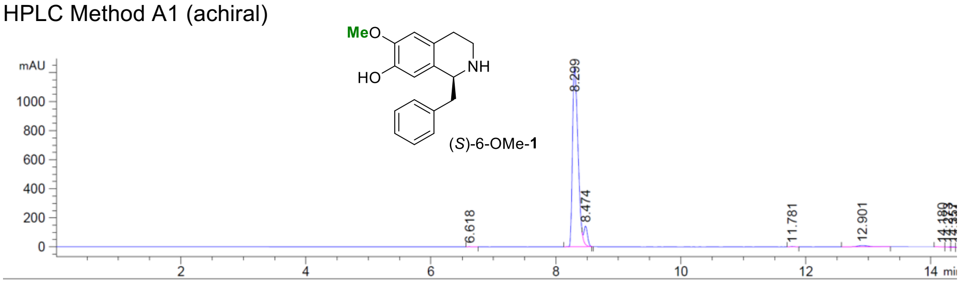

Calibration curve

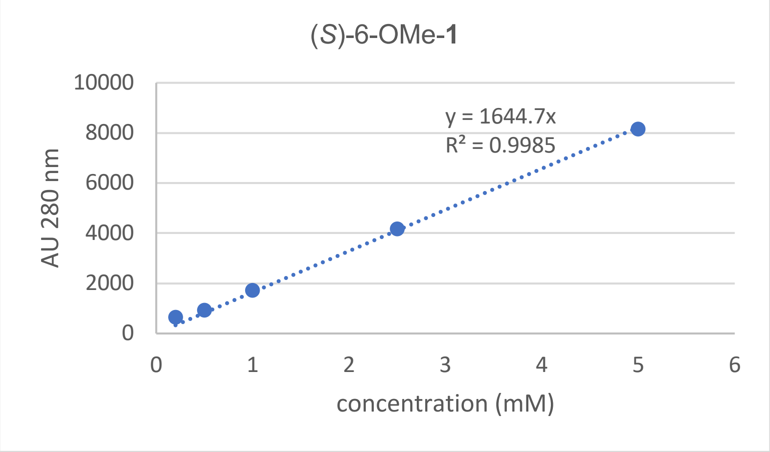

HPLC method C1 (chiral) (*S*)-**1** (>97% e.e.)

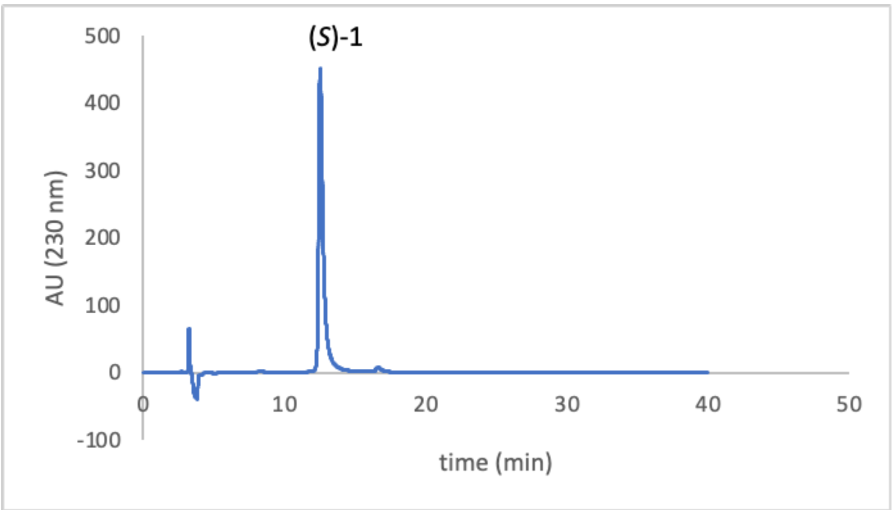

HPLC method C1 (chiral), rac-**1**

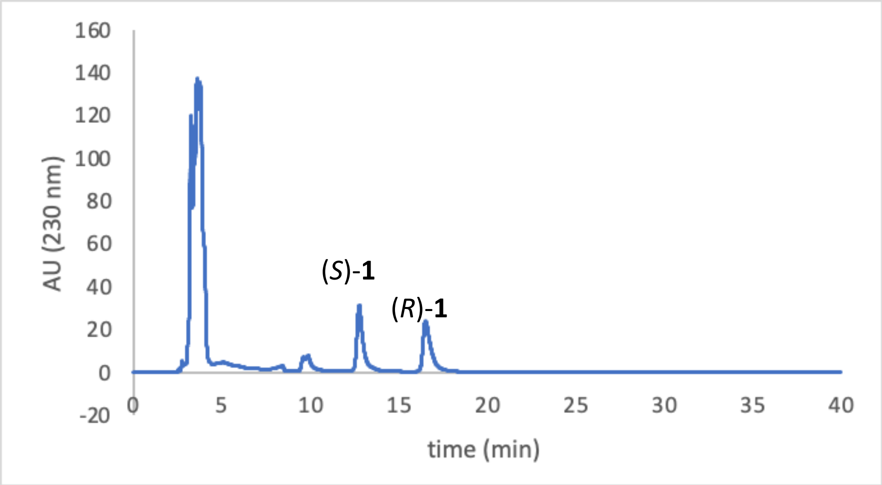

HPLC method C1 (chiral) (*S*)-6-OMe-**1**

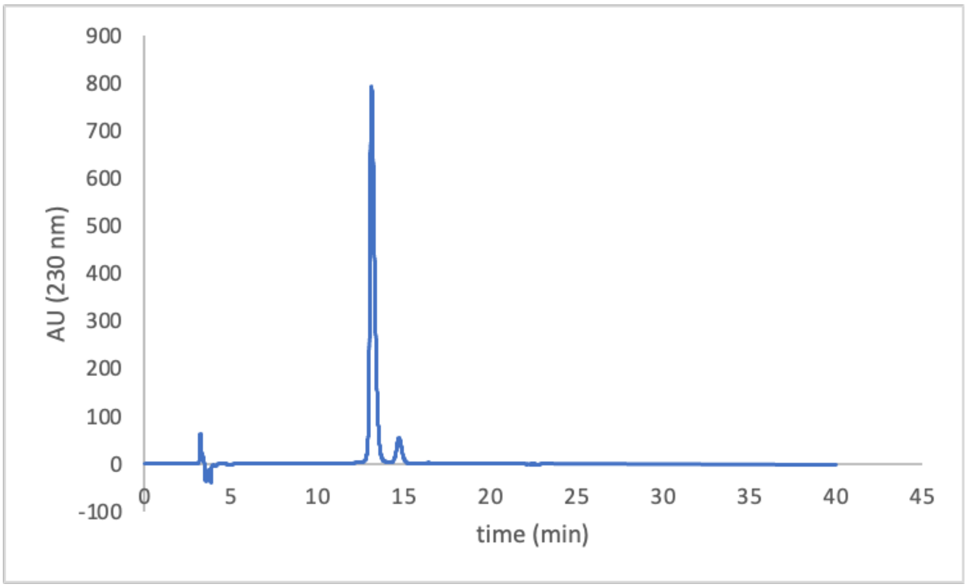

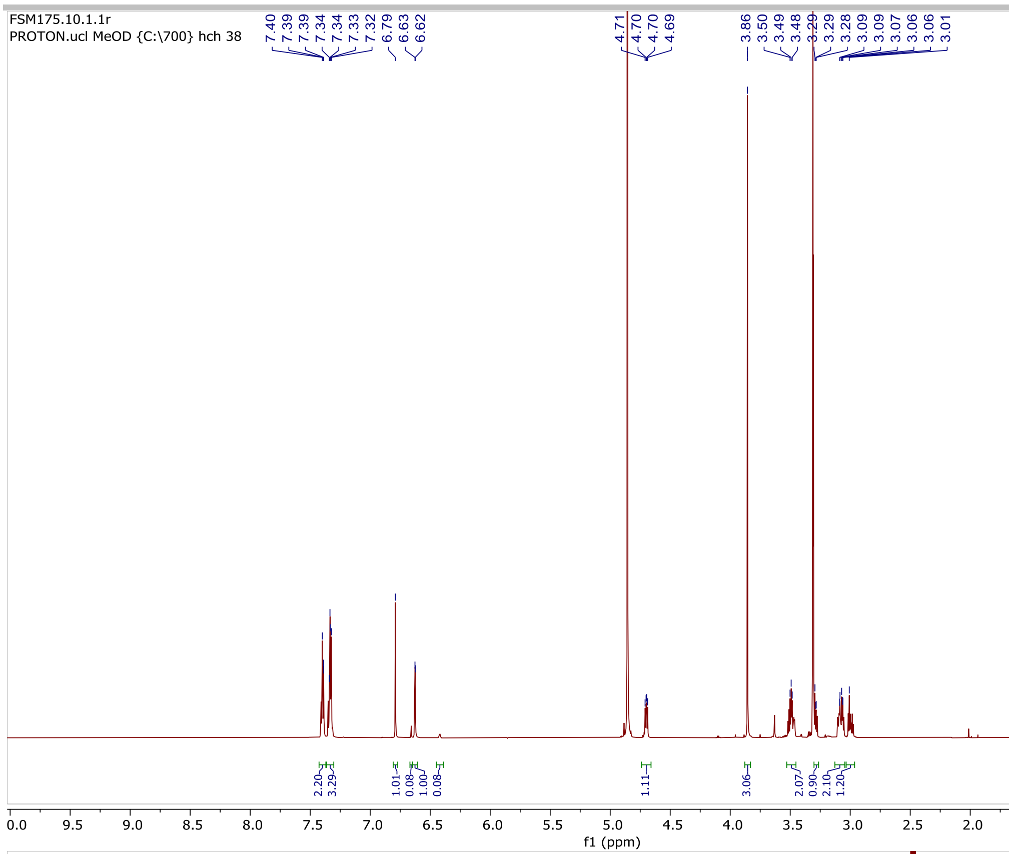
FSM175.10.1.1r
PROTON.ucl MeOD {C:\700} hch 38
7.40
7.39
7.39
7.34
7.34
7.33
7.32
6.79
6.63
6.62
4.71
4.70
4.70
4.69
3.86
3.50
3.49
3.48
3.29
3.29
3.28
3.09
3.09
3.07
3.06
3.06
3.01
2.20
3.29
1.01
0.08
1.00
0.08
1.11
3.06
2.07
0.90
2.10
1.20
f1 (ppm)

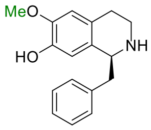
MeO
HO
NH

(*S*)-6-OMe-**1**

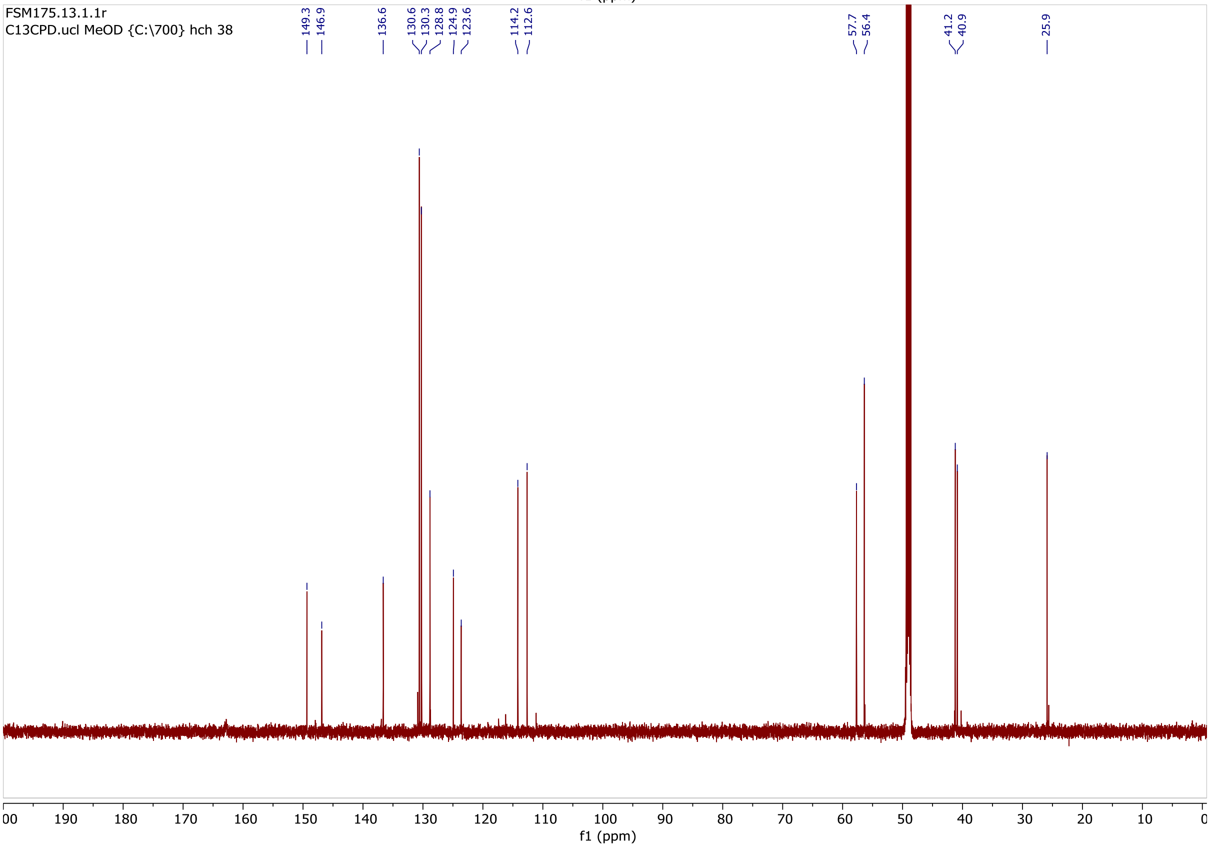
FSM175.13.1.1r
C13CPD.ucl MeOD {C:\700} hch 38
149.3
146.9
136.6
130.6
130.3
128.8
124.9
123.6
114.2
112.6
57.7
56.4
41.2
40.9
25.9
f1 (ppm)

Five-enzyme cascade to (*S*)-1-benzyl-6-methoxy-2-methyl-1,2,3,4-tetrahydroisoquinolin-7-ol (*S*)-2-NMe-6-OMe-1

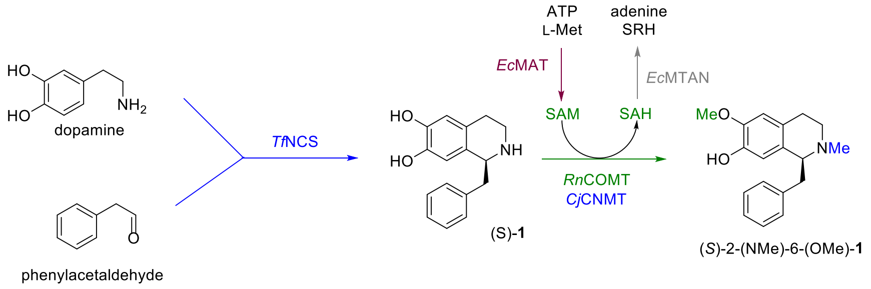

Dopamine hydrochloride (9.45 mg, 0.05 mmol), phenylacetaldehyde (8.4 µL in 0.5 mL of CH_3CN, 0.075 mmol), sodium ascorbate (9.9 mg, 0.05 mmol), and purified *Tf*NCS (2.5 mg) were combined with HEPES (50 mM pH 7.5) to a total volume of 5 mL. The reaction was stirred at 37 °C until completion. Once the reaction had completed, the yellow suspension was concentrated under reduced pressure and the residue dissolved in HEPES (50 mM pH 7.5) containing 20 mM $MgCl_2$ and 100 mM NaCl. The methylation step was initiated in the same pot and all the components were added to have a final concentration of 15 mM ATP (82.5 mg, 0.15 mmol), 15 mM of L-methionine (0.15 mmol, 22.4 mg), 0.4 mg/mL of *Ec*MAT, 0.1 mg/mL of *Ec*MTAN, 0.6 mg/mL of *Cj*NMT, and 10 % v/v of *Rn*COMT lysate in a total volume of 10 mL. The reaction was started upon enzymes addition and incubated overnight at 37 °C at 180 rpm. After this time an aliquot was taken to calculate the yield by HPLC against the standard product (see the calibration curve below). The reaction was quenched with 1 M HCl (1 mL) and the product was purified using preparative HPLC (method B, retention time: 16.0 min). Fractions containing the desired product were freeze-dried to give (*S*)-2-NMe-6-(OMe)-**1** as a powder (TFA salt, 8.7 mg, 46% yield, 66% yield by HPLC). Trace impurities (< 5%) were also identified by NMR corresponding to the regioisomeric product methylated at *O*-7. HRMS (ES+) found [M+H]+ 284.1647; $C_{18}H_{20}NO_2$ requires 284.1651.

The product was characterized as a free base and only signals for the major regioisomer are given:

^{1}H NMR (500 MHz, MeOD) δ 7.28 – 7.21 (m, 2H, Ar-H), 7.18 (d, *J* = 7.3 Hz, 1H, Ar-H), 7.13 – 7.06 (m, 2H, Ar-H), 6.64 (s, 1H, 5-H), 6.09 (s, 1H, 8-H), 3.80 (s, 3H, -OMe), 3.78 (dd, *J* = 7.5, 5.2 Hz, 1H, 1-H), 3.26 – 3.13 (m, 2H, 3-*H*H and NHCHCH*H*), 2.91 – 2.82 (m, 2H, NHCHCH*H* and 4-*H*H), 2.76 (ddd, *J* = 12.1, 6.0, 3.7 Hz, 1H, 3-H), 2.71 – 2.60 (m, 1H, 4-H), 2.49 (s, 3H, -NMe); ^{13}C NMR (126 MHz, MeOD) δ 173.0, 147.9, 145.2, 140.8, 2 x 130.7, 2 x 129.3, 127.2, 125.3, 115.6, 112.6, 65.9, 56.3, 47.5, 42.5, 41.5, 25.9.

Another *N*-methylation reaction was also carried out on (*S*)-6-OMe-**1** using *Ec*MAT and *Ec*MTAN as clarified lysate giving the same product (*S*)-2-NMe-6-OMe-**1** and the experimental details are reported below.

Compound (*S*)-**1** (TFA salt, 8.0 mg, 0.02 mmol), ATP (16.5 mg, 0.03 mmol), and L-methionine (5.0 mg, 0.03 mmol) were combined in HEPES buffer (50 mM pH 7.5). The methylation step was initiated upon addition of 10% v/v of *Ec*MAT lysate, 2% v/v of *Ec*MTAN lysate, and 1 mg/mL of pure *Cj*NMT in a total volume of 5 mL containing 20 mM $MgCl_2$ and 200 mM KCl. The reaction was incubated overnight at 37 °C and 180 rpm. After this time the reaction was centrifuged, the supernatant was basified with $NaHCO_3$ and extracted with EtOAc (3 x 10 mL). The organic fractions were collected, evaporated under vacuum and the residue purified using preparative HPLC (method B). Fractions containing the desired product were freeze-dried to give (*S*)-2-NMe-6-OMe-**1** as a powder (TFA salt, 3.5 mg, 49% yield, 59% yield by HPLC). The characterization data agreed with that above.

HPLC Method A1

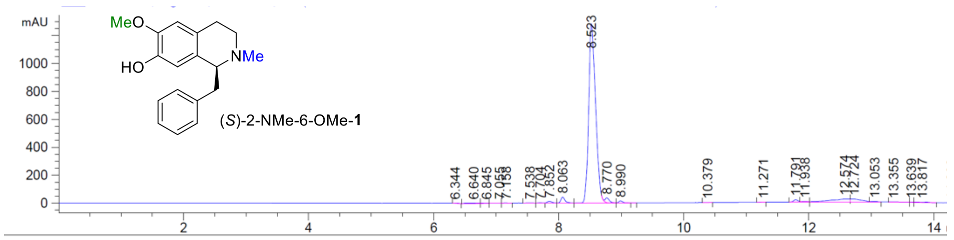

Calibration curve

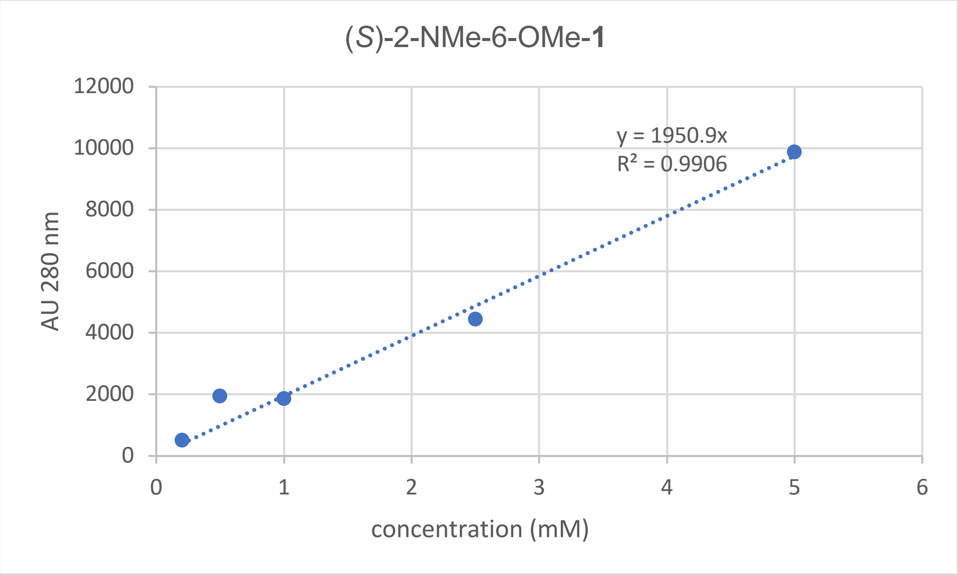

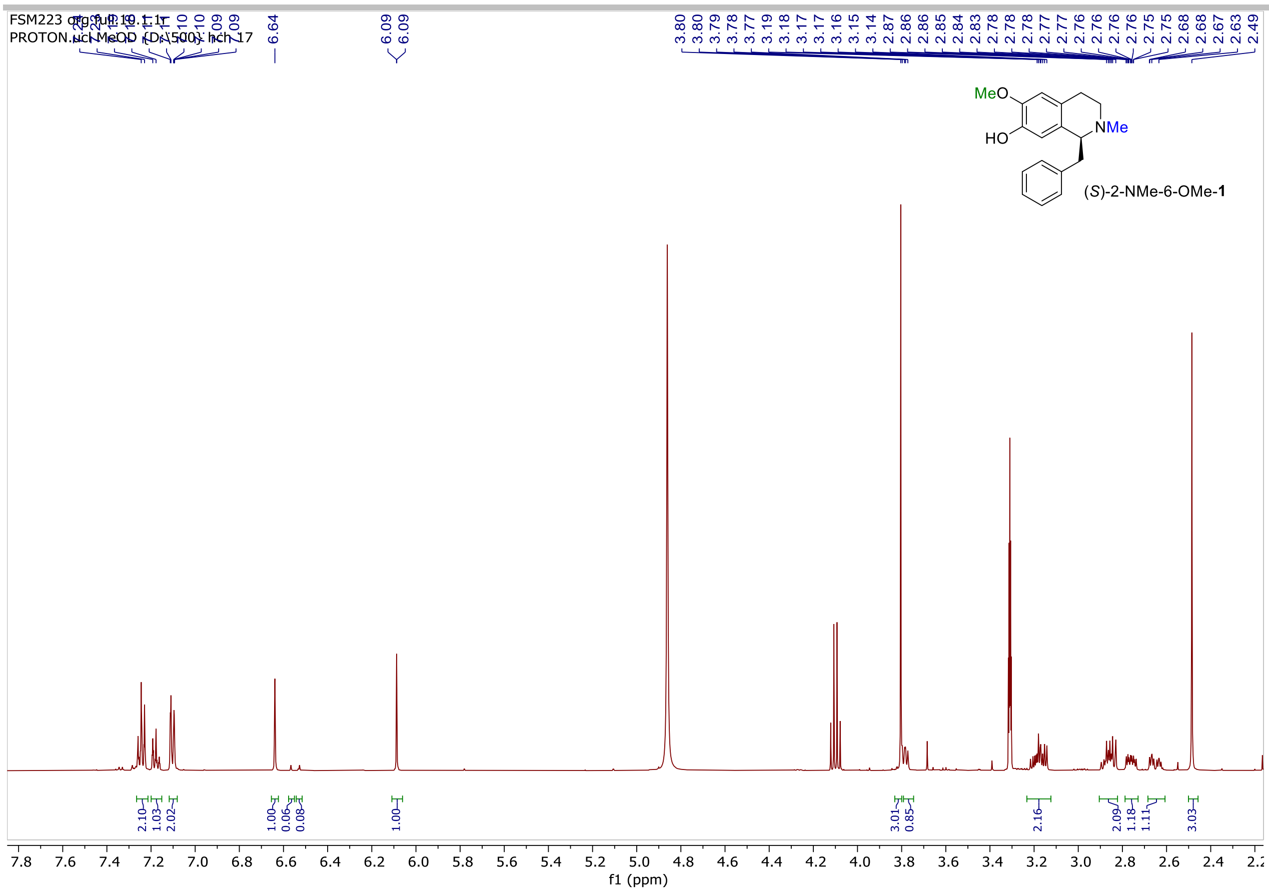
FSM223 org full.19.1.1r
PROTON.ucl MeOD {D:\500} hch 17
(S)-2-NMe-6-OMe-1
MeO
HO
NMe
f1 (ppm)

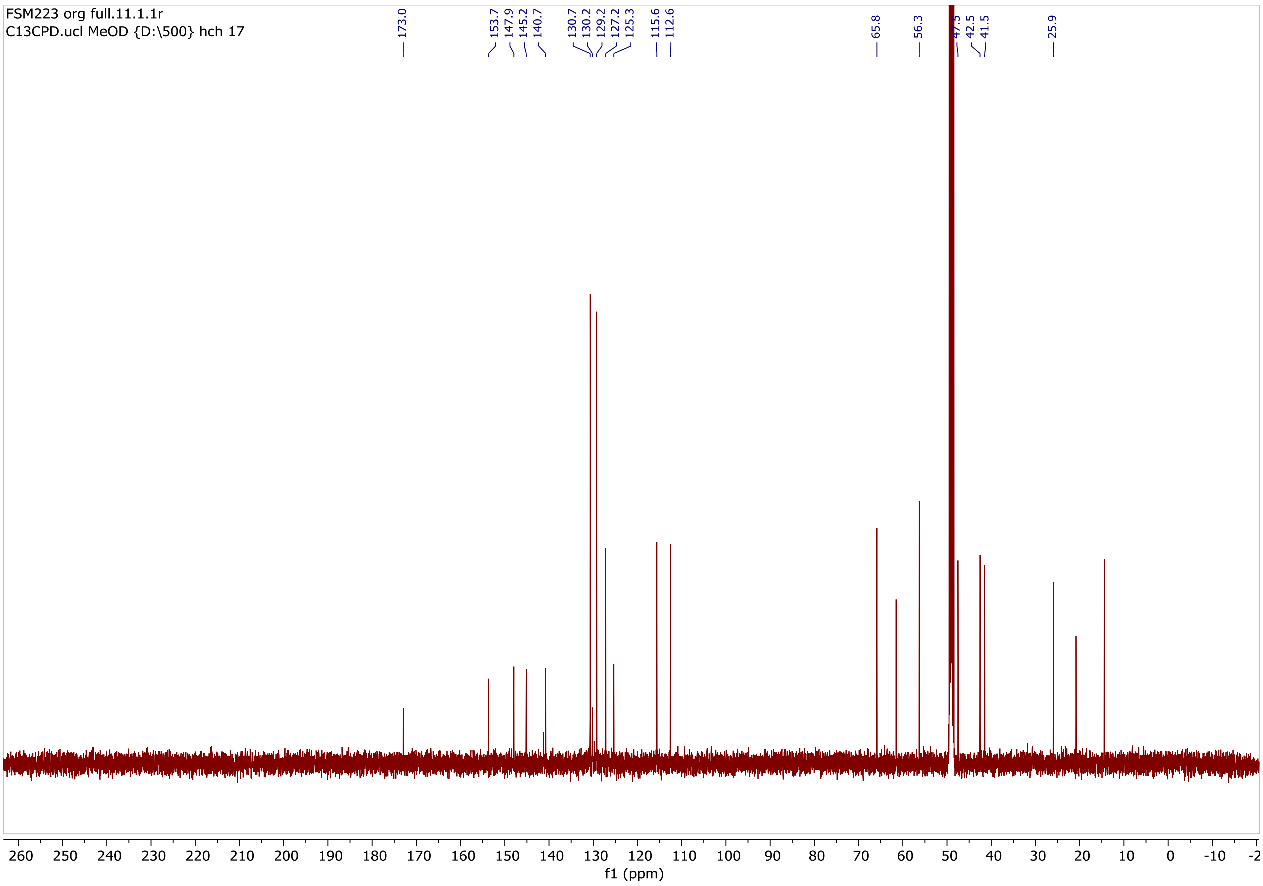
FSM223 org full.11.1.1r
C13CPD.ucl MeOD {D:\500} hch 17
173.0
153.7
147.9
145.2
140.7
130.7
130.2
129.2
127.2
125.3
115.6
112.6
65.8
56.3
42.5
41.5
25.9
f1 (ppm)

Seven-enzymes cascade to (*S*)-1-(4-hydroxybenzyl)-6-methoxy-1,2,3,4-tetrahydroisoquinolin-7-ol (*S*)-6-OMe-2

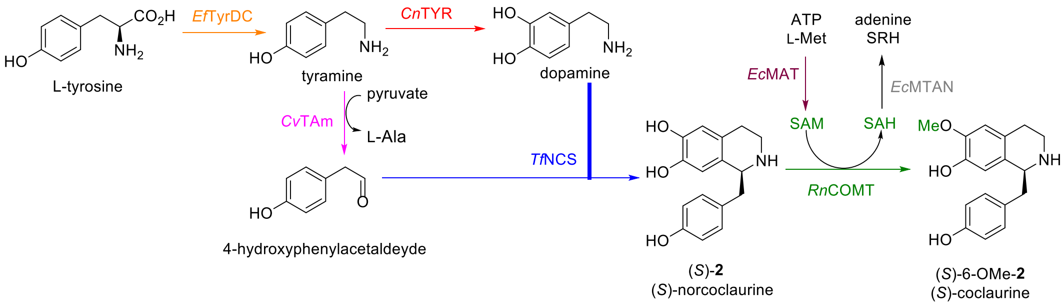

The reaction mixture (50 mL, pH 5.5) consisted of 50 mM HEPES, 20 mM L-tyrosine hydrochloride salt, 50 mM sodium ascorbate, 100 µM $CuSO_4{\cdot}5H_2O$, 5 mM PLP and 10 mM sodium pyruvate. To initiate the decarboxylation step, 10% v/v of *Ef*TyrDC lysate was added to the reaction mixture which was incubated at 25 °C, 250 rpm for 8 h. Then, the reaction mixture was adjusted to pH 7.5 with 2.5 M NaOH, and the next steps performed with 5% v/v of *Cn*TYR lysate, 10% v/v of *Cv*TAm lysate and 50 µg/mL of *Tf*NCS at 37 °C, 250 rpm for 8 h. The reaction mixture was then centrifuged, and 40 mM $MgCl_2$, 200 mM KCl, 5 mM ATP and 5 mM L-methionine were added to the supernatant. To initiate the methylation step, 10% (v/v) of *Rn*COMT cell lysate, 10% (v/v) of *Ec*MAT cell lysate and 2.5% v/v of *Ec*MTAN lysate were added and the reaction mixture was incubated at 37 °C, 250 rpm for another 12 h. After this time the reaction was centrifuged, and the supernatant was extracted with EtOAc (3 x 20 mL). The organic fractions were collected, evaporated under vacuum, and the residue purified using preparative HPLC (method B). Fractions containing the desired product were freeze-dried to give (*S*)-6-OMe-**2** as an off-white powder (TFA salt, 26.2 mg, 55%, 92% yield by HPLC). Trace impurities (5%) were also identified by NMR corresponding to the regioisomeric product methylated at *O*-7. ^{1}H NMR (700 MHz; MeOD) δ = 7.12 (d, *J* = 8.4 Hz, 2H, 2'-H and 6'-H), 6.80 (d, *J* = 8.4 Hz, 2H, 3'-H and 5'-H), 6.76 (s, 1H, 5-H), 6.63 (s, 1H, 8-H), 4.59 (dd, *J* = 8.4, 6.0 Hz, 1H, 1-H), 3.84 (s, 3H, OMe), 3.47 (dt, *J* = 12.5, 6.0 Hz, 1H, 3-*H*H), 3.36-3.32 (m, 1H, NHCHC*H*H), 3.26 (ddd, *J* = 12.5, 7.3, 6.0, 1H, 3-H*H*), 3.05 (dt, *J* = 16.9, 6.0, 1H, 4-*H*H), 3.00-2.95 (m, 2H, 4-H*H* and NHCHCH*H*); ^{13}C (176 MHz; MeOD) δ = 158.2, 149.2, 146.7, 131.7, 127.0, 125.0, 123.7, 117.0, 114.2, 112.1, 57.8, 56.4, 40.8, 40.4, 25.9; HRMS (ES+) found $[M+H]^+$ 286.1443; $C_{17}H_{20}NO_3$ requires 286.1365; $[\alpha]_D^{25}$ -13.3 (c 0.14, MeOH).[5]

HPLC Method A2 (achiral)

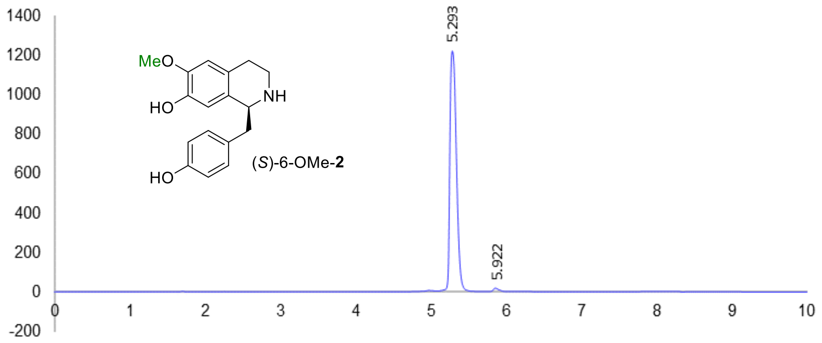

Calibration curve

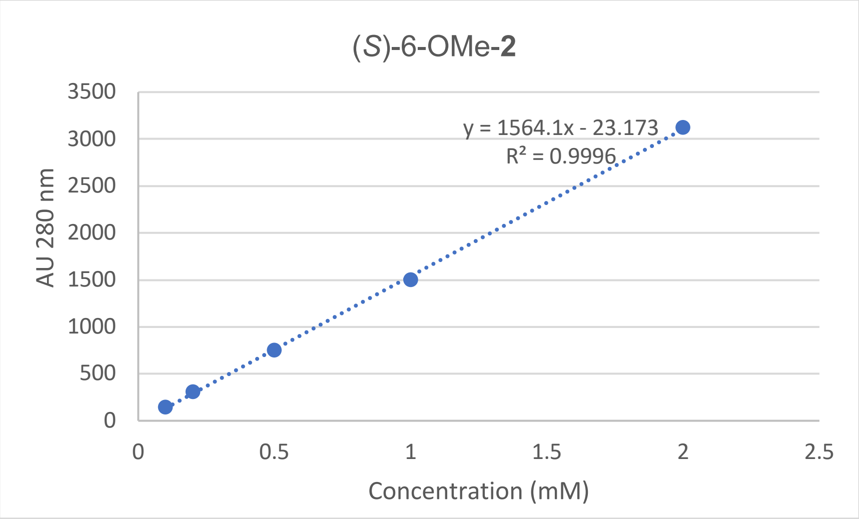

HPLC method C1 (chiral) (*S*)-**2**[4]

e.e. > 97%

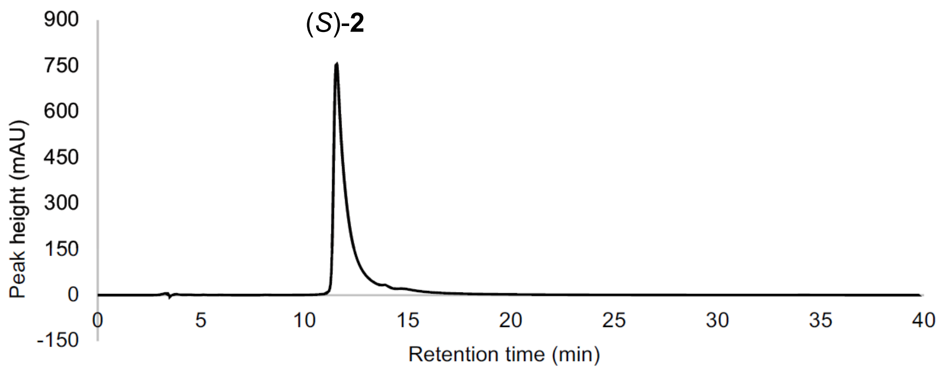

HPLC method C1 (chiral) rac-**2**[4]

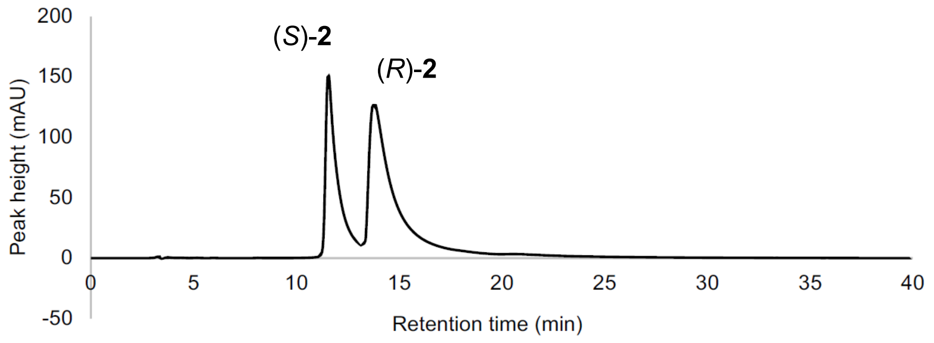

HPLC method C1 (chiral) (*S*)-6-OMe-**2**

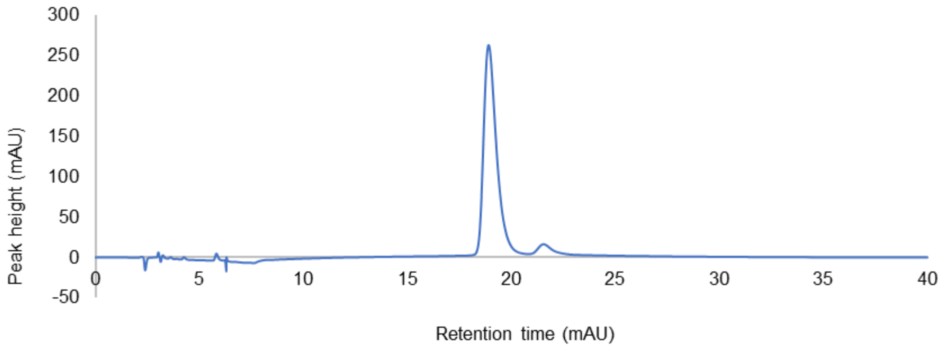

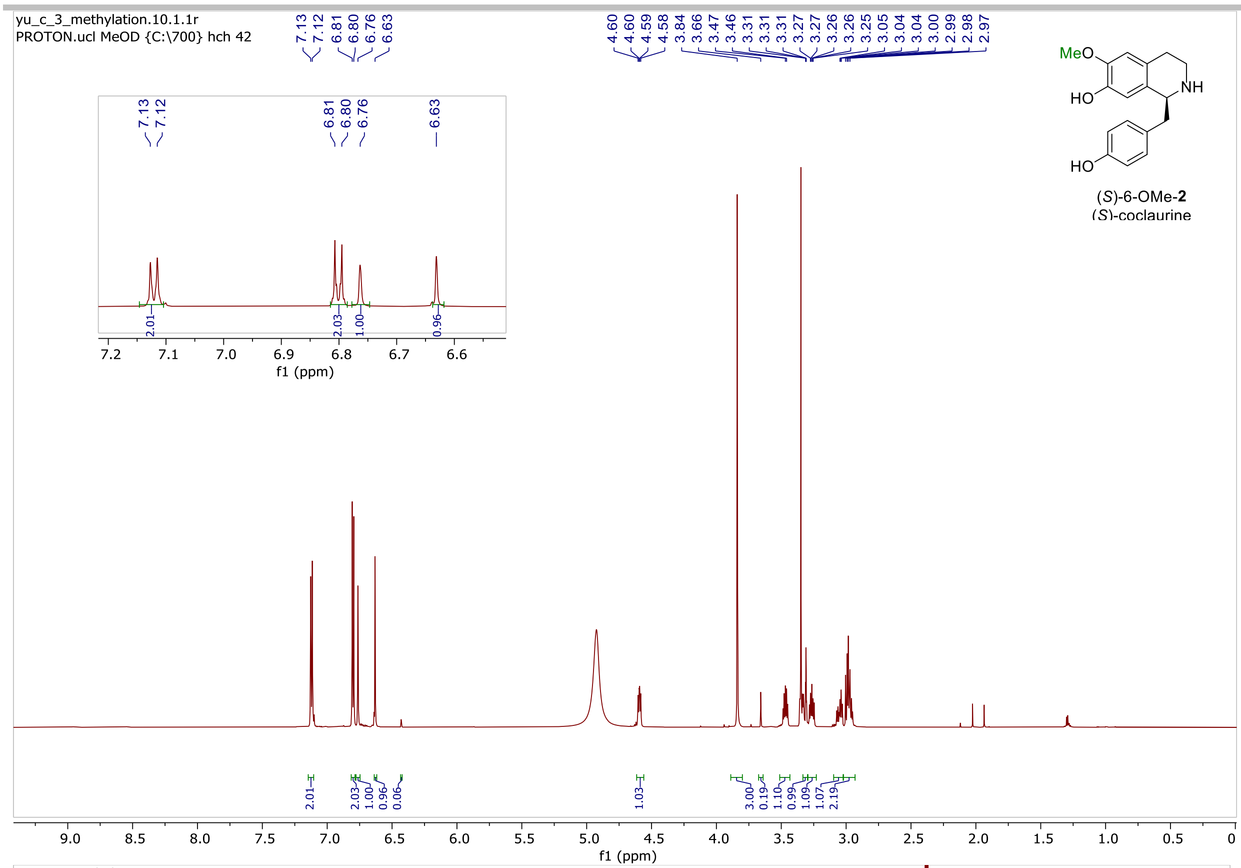
yu_c_3_methylation.10.1.1r
PROTON.ucl MeOD {C:\700} hch 42
7.13
7.12
6.81
6.80
6.76
6.63
4.60
4.60
4.59
4.58
3.84
3.66
3.47
3.46
3.31
3.31
3.31
3.27
3.27
3.26
3.26
3.25
3.05
3.04
3.04
3.00
2.99
2.98
2.97
MeO
HO
NH
HO
(S)-6-OMe-2
(S)-coclaurine
2.01
2.03
1.00
0.96
0.06
1.03
3.00
0.19
1.10
0.99
1.09
1.07
2.19
f1 (ppm)

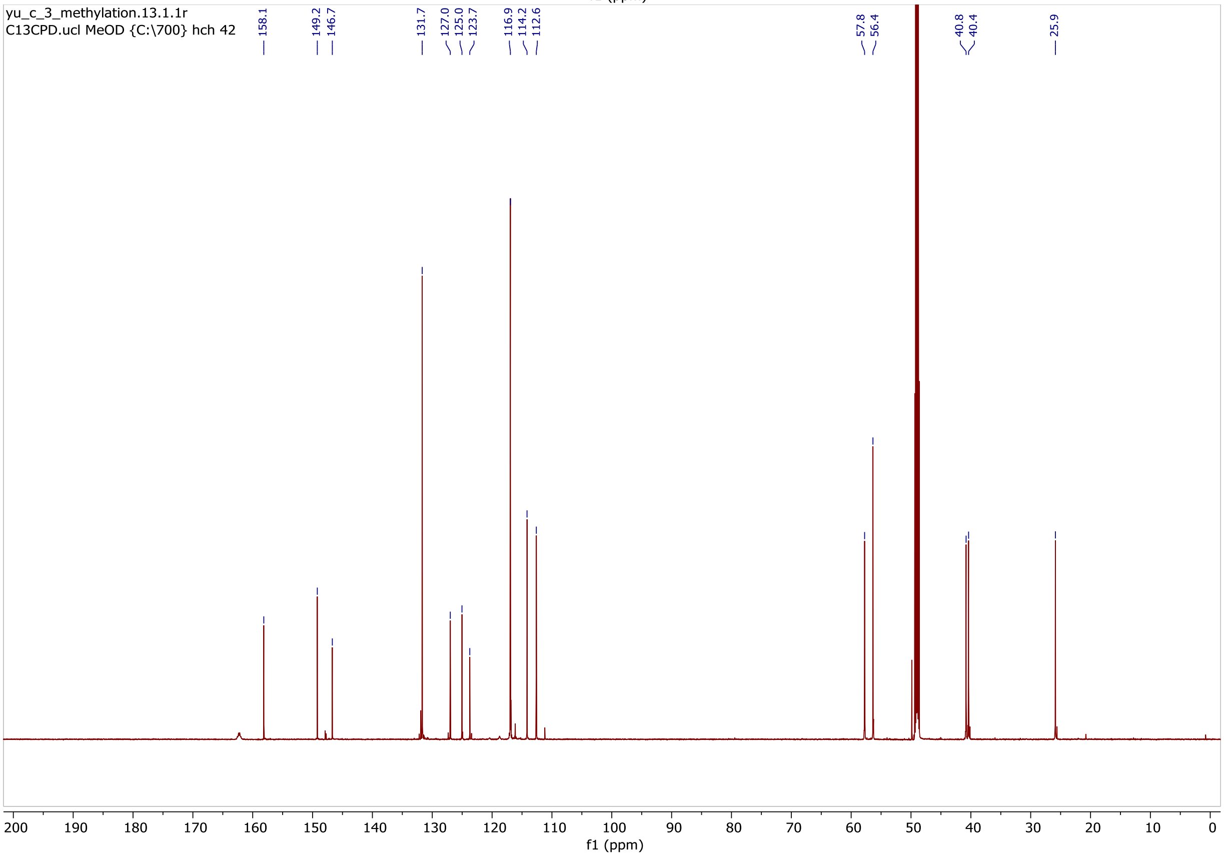
yu_c_3_methylation.13.1.1r
C13CPD.ucl MeOD {C:\700} hch 42
158.1
149.2
146.7
131.7
127.0
125.0
123.7
116.9
114.2
112.6
57.8
56.4
40.8
40.4
25.9
f1 (ppm)

Seven-enzymes cascade to (*S*)-1-(4-hydroxybenzyl)-6-methoxy-1,2,3,4-tetrahydroisoquinolin-7-ol (*S*)-6-OMe-2, (*S*)-1-(4-hydroxybenzyl)-7-methoxy-1,2,3,4-tetrahydroisoquinolin-6-ol (*S*)-7-OMe-2, and ((*S*)-4-((6,7-dimethoxy-1,2,3,4-tetrahydroisoquinolin-1-yl)methyl)phenol (*S*)-6,7-(OMe)₂-2

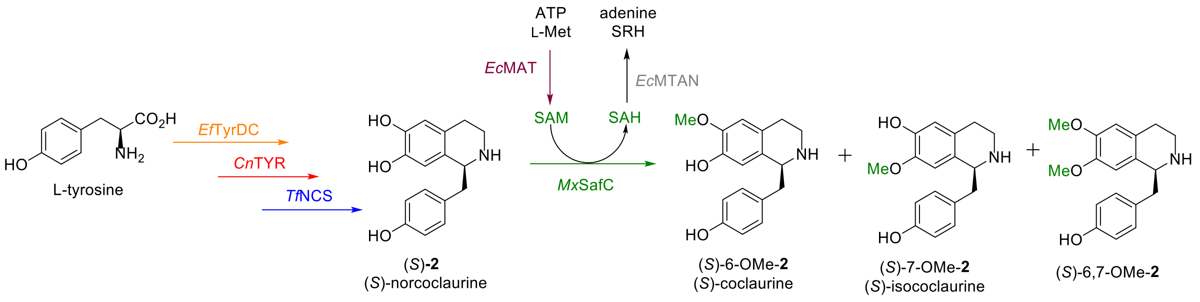

The reaction mixture (100 mL, pH 5.5) consisted of 50 mM HEPES, 2.5 mM L-tyrosine hydrochloride salt, 10 mM sodium ascorbate, 40 μM $CuSO_4 \cdot 5H_2O$, 1.25 mM PLP and 1.25 mM sodium pyruvate. To initiate the decarboxylation step, 10% v/v of *Ef*TyrDC cell lysate was added to the reaction mixture which was incubated at 25 °C, 250 rpm for 8 h. Then, the reaction mixture was adjusted to pH 7.5 with 2.5 M NaOH, and the next steps performed with 5% v/v of *Cn*TYR lysate, 10% v/v of *Cv*TAm lysate and 50 μg/mL of *Tf*NCS at 37 °C, 250 rpm for 8 h. The reaction mixture was then centrifuged, and 40 mM $MgCl_2$, 200 mM KCl, 5 mM ATP and 5 mM L-methionine were added to the supernatant. To initiate the methylation step, 10% v/v of *Rn*COMT lysate, 10% v/v of *Ec*MAT lysate and 2.5% v/v of *Ec*MTAN lysate were added and the reaction mixture, which was incubated at 37 °C, 250 rpm for another 12 h. The reaction was centrifuged, and the supernatant was extracted with EtOAc (3 x 20 mL). The organic fractions were collected, evaporated under vacuum, and the residue purified using preparative HPLC (method B). Fractions containing the desired products were freeze-dried to give (*S*)-6-OMe-**2** and (*S*)-7-OMe-**2** as mixture (a powder) (TFA salt, 20.5 mg, 43%, 89% yield by HPLC). Fractions containing the desired product were freeze-dried to give (S)-6,7-(OMe)₂-**2** (off-white powder) (TFA salt, 2.9 mg, 6%, 9% yield by HPLC).

Compound (*S*)-7-OMe-**2**: ^{1}H NMR (700 MHz; CD_3OD) δ = 7.10 (app.dt, *J* = 7.0, 2.8 Hz, 2H, 2'-H and 6'-H), 6.81-6.79 (m, 2H, 3'-H and 5'-H), 6.64 (s, 1H, 5-H), 6.44 (s, 1H, 8-H), 4.59 (app. dd, *J* = 9.1, 5.6 Hz, 1H, 1-H), 3.66 (s, 3H, 7-OMe), 3.50 (app. ddd, *J* =12.6, 7.7, 5.6 Hz, 1H, 3-H*H*), 3.32-3.31 (m, 1H, NHCHCH*H*), 3.30-3.29 (m, 1H, 3-*H*H), 3.06-3.02 (m, 1H, 4-H*H*), 3.00-2.92 (m, 2H, NHCHC*H*H and 4-*H*H); ^{13}C (176 MHz; MeOD) δ = 158.1, 147.9, 147.7, 131.8, 127.2, 124.8, 123.3, 116.8, 116.0, 111.1, 57.7, 56.2, 40.4, 40.1, 25.5; HRMS (ES+) found [M+H]+ 286.1437; $C_{17}H_{20}NO_3$ requires 286.1437.

Compound (*S*)-6,7-(OMe)₂-**2**: ^{1}H NMR (700 MHz; CD_3OD) δ = 7.10 (app.dt, *J* = 8.4, 2.8 Hz, 2H, 2'-H and 6'-H), 6.80 (app.dt, *J* = 9.1, 2.8 Hz, 2H, 3'-H and 5'-H), 6.79 (s, 1H, 8-H), 6.47 (s, 1H, 5-H), 4.64 (t, *J* = 7.7 Hz, 1H, 1-H), 3.81 (s, 3H, 6-OMe), 3.64 (s, 3H, 7-OMe), 3.53 (app. ddd, *J* =12.6, 7.7, 5.6 Hz, 1H, 3-H*H*), 3.35-3.32 (m, 2H, NHCHCH*H* and 3-*H*H), 3.07-3.00 (m, 3H, NHCHC*H*H, 4-H*H* and 4-HH); ^{13}C NMR (176 MHz; CD_3OD) δ = 158.2, 150.4, 149.1, 132.4, 131.8, 127.0, 124.7, 116.8, 112.7, 111.3, 57.6, 56.3, 56.2, 40.3, 40.0, 25.7; HRMS (ES+) found $[M+H]^+$ 300.1594; $C_{18}H_{22}NO_3$ requires 300.1594; $[\alpha]_D^{25}$ - 21.5 (c 0.2, MeOH).

HPLC Method A2 (achiral)

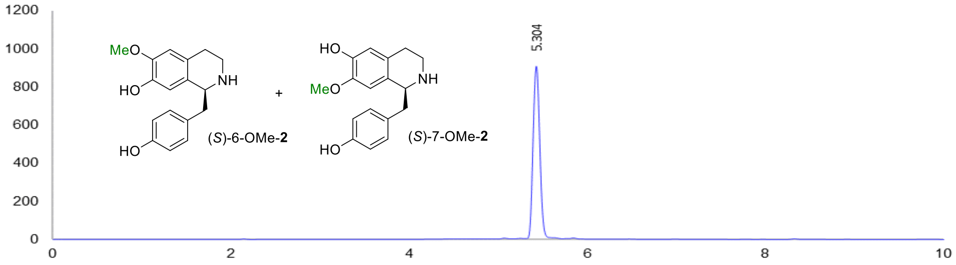

When using a 40 min HPLC method: 0.0 min (95 % H_2O/5 % MeCN)-5 min (95 % H_2O/5 % MeCN)-30 min (30 % H_2O/70 % MeCN)-31 min (100 % MeCN)-35 min (100 % MeCN)-36 min (95 % H_2O/5 % MeCN)-40 min (95 % H_2O/5 % MeCN).

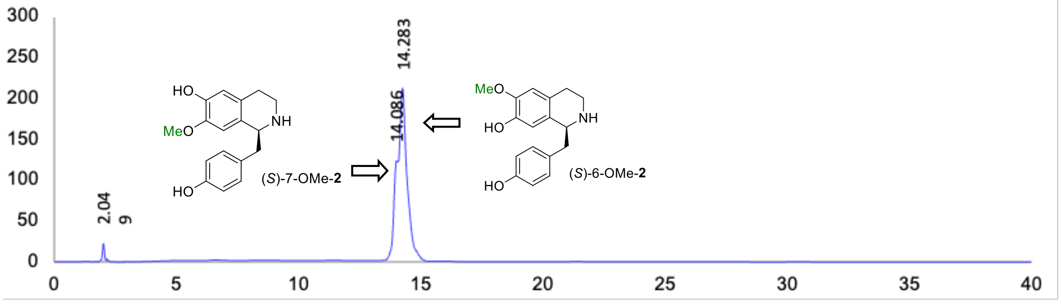

HPLC Method A2 (achiral)

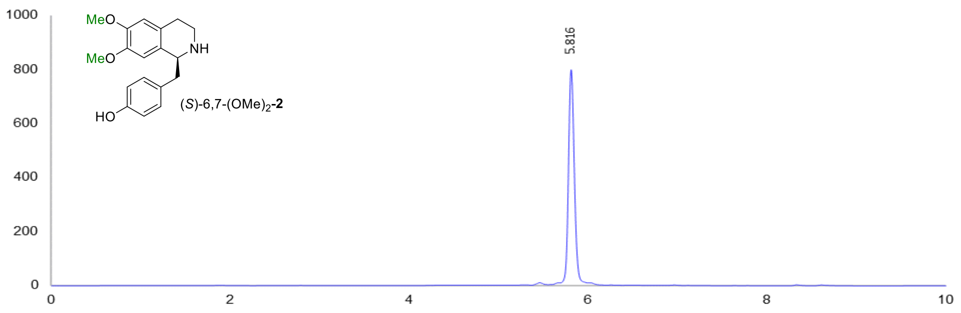

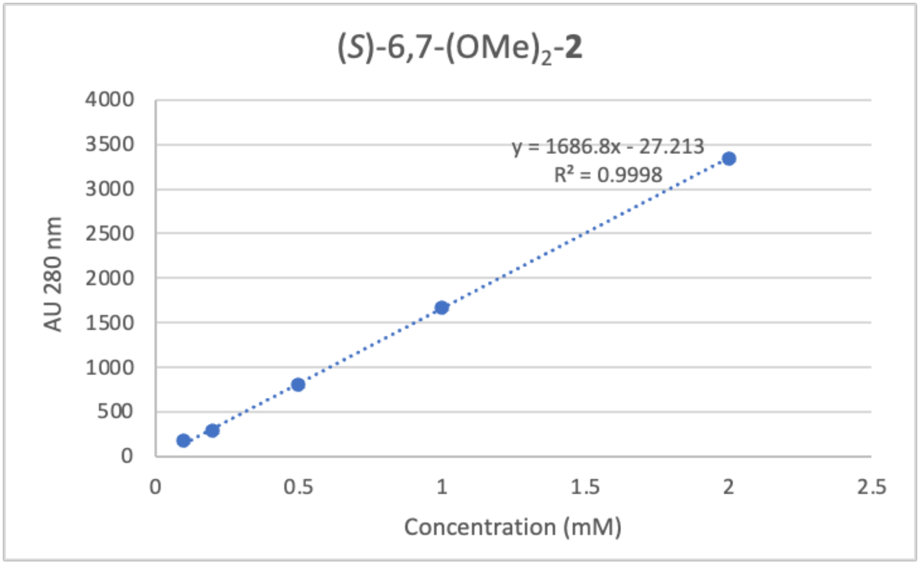

HPLC method C1 (chiral) (*S*)-6,7-$(OMe)_2$-**2**

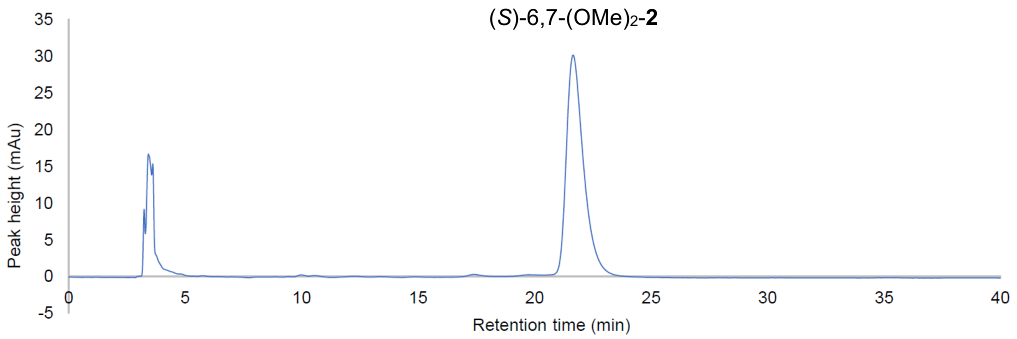

HPLC method C1 (chiral) rac-6,7-$(OMe)_2$-**2** (rac-**2** prepared following the literature[6] which was methylated as described above)

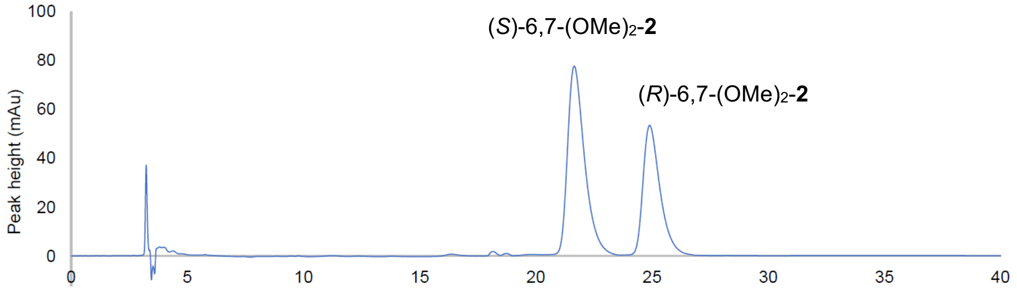

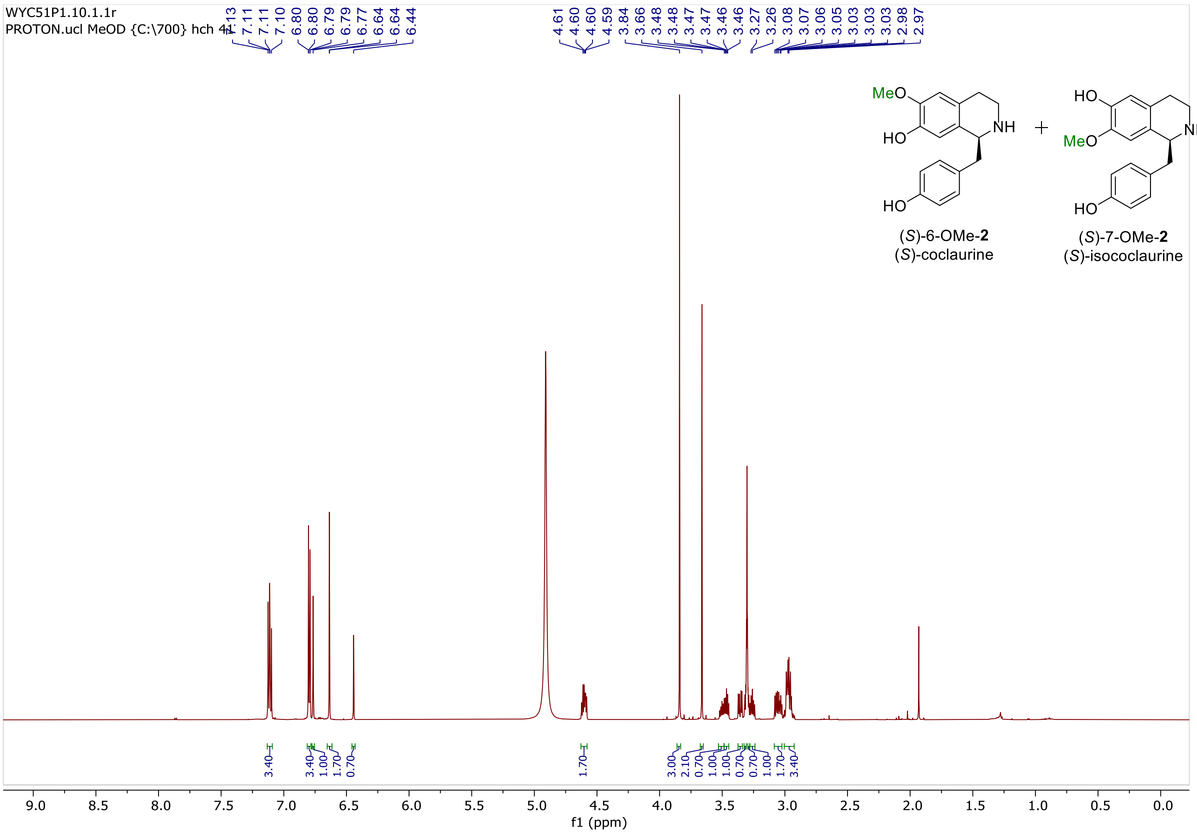
WYC51P1.10.1.1r
PROTON.ucl MeOD {C:\700} hch 41
7.13 7.11 7.11 7.10 6.80 6.79 6.79 6.77 6.64 6.64 6.44
4.61 4.60 4.60 4.59 3.84 3.66 3.48 3.48 3.47 3.47 3.46 3.46 3.27 3.26 3.08 3.07 3.06 3.05 3.03 3.03 3.03 2.98 2.97
MeO
HO
NH
HO
(S)-6-OMe-2
(S)-coclaurine
+
HO
MeO
NH
HO
(S)-7-OMe-2
(S)-isococlaurine
3.40 3.40 1.00 1.70 0.70 1.70 3.00 2.10 0.70 1.00 1.00 0.70 0.70 1.00 1.70 3.40
f1 (ppm)

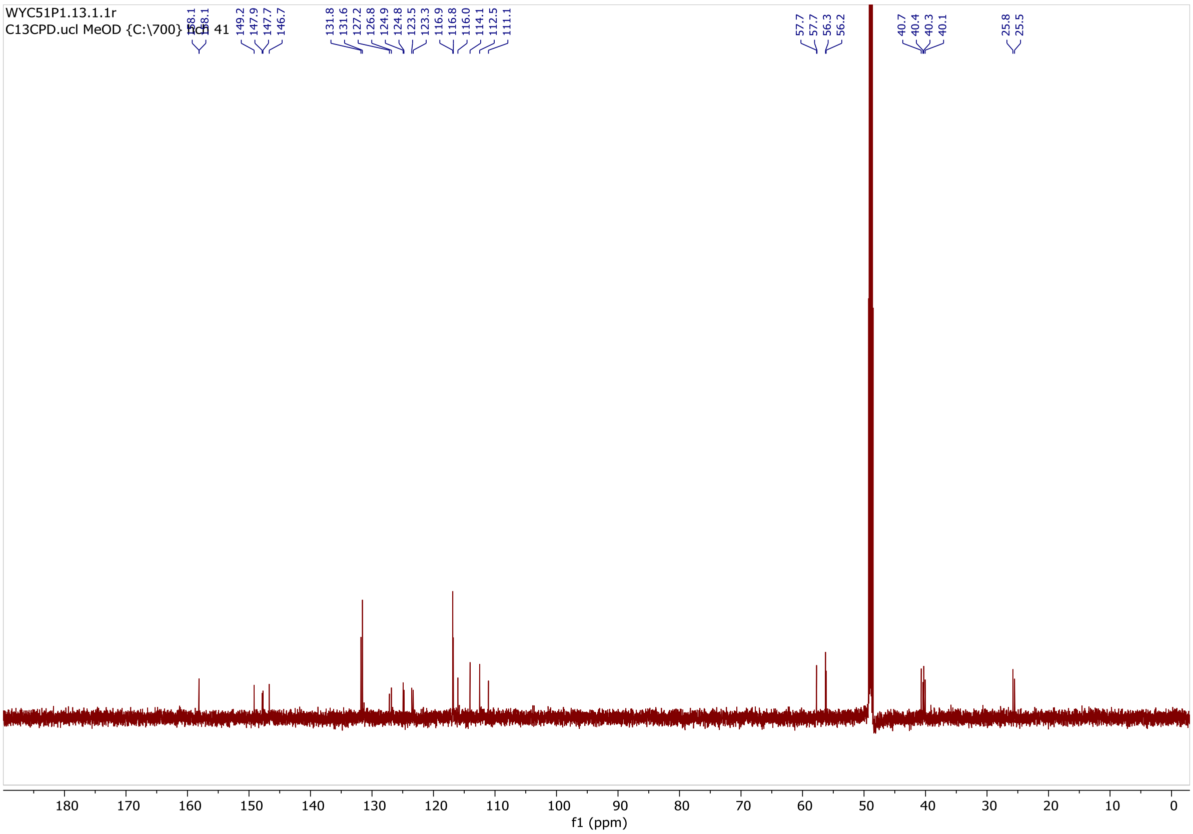
WYC51P1.13.1.1r
C13CPD.ucl MeOD {C:\700} hch 41
158.1 158.1 149.2 147.9 147.7 146.7 131.8 131.6 127.2 126.8 124.9 124.8 123.5 123.3 116.9 116.8 116.0 114.1 112.5 111.1
57.7 57.7 56.3 56.2 40.7 40.4 40.3 40.1 25.8 25.5
f1 (ppm)

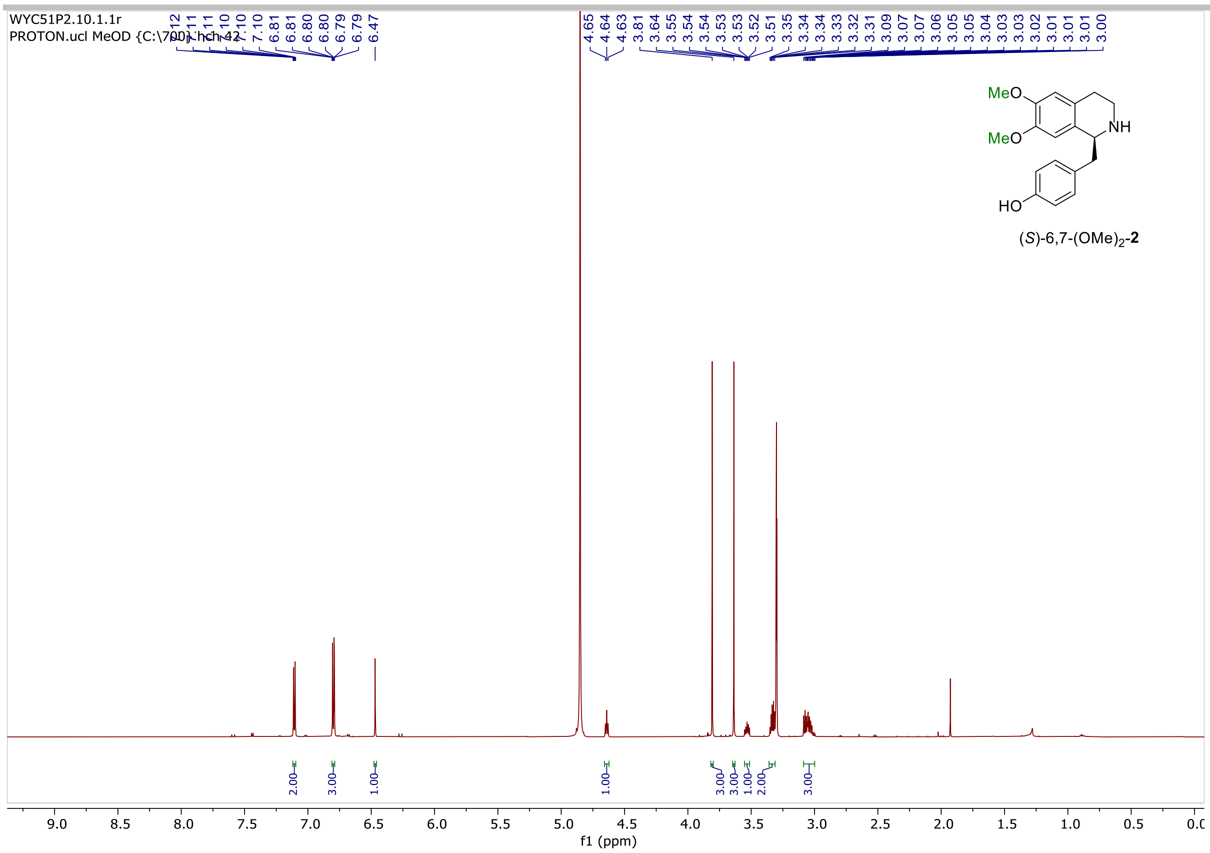
WYC51P2.10.1.1r
PROTON.ucl MeOD {C:\700} hch 42
7.12 7.11 7.11 7.10 7.10 7.10 6.81 6.81 6.80 6.79 6.79 6.47
4.65 4.64 4.63 3.81 3.64 3.55 3.54 3.54 3.53 3.53 3.52 3.51 3.35 3.34 3.34 3.33 3.32 3.31 3.09 3.07 3.07 3.06 3.05 3.05 3.04 3.03 3.03 3.02 3.01 3.01 3.01 3.00
MeO
MeO
NH
HO
(S)-6,7-(OMe)2-2
2.00 3.00 1.00 1.00 3.00 3.00 1.00 2.00 3.00
f1 (ppm)

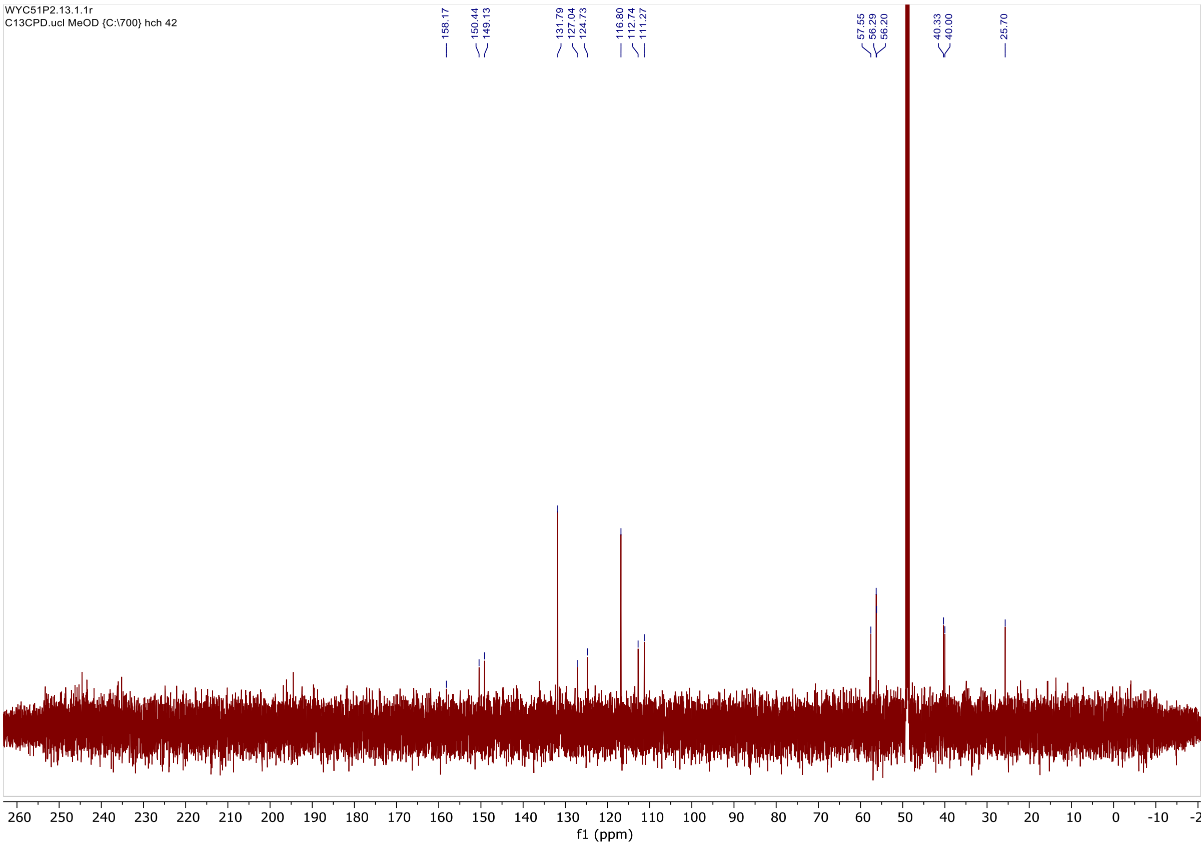
WYC51P2.13.1.1r
C13CPD.ucl MeOD {C:\700} hch 42
158.17 150.44 149.13 131.79 127.04 124.73 116.80 112.74 111.27 57.55 56.29 56.20 40.33 40.00 25.70
f1 (ppm)

Five-Enzymes Cascade to (*S*)-1-(4-hydroxy-3-methoxybenzyl)-6-methoxy-1,2,3,4-tetrahydroisoquinolin-7-ol (*S*)-6,3'-(OMe)$_2$-3

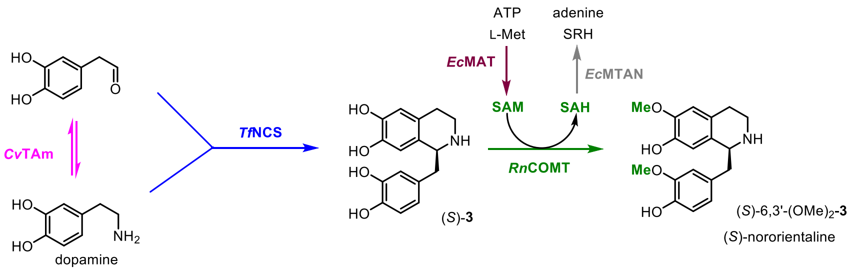

Dopamine hydrochloride (19 mg, 0.1 mmol), sodium ascorbate (20 mg, 0.1 mmol), sodium pyruvate (5.5 mg, 0.05 mmol), *Cv*TAm 10% v/v lysate, and 10% v/v of *Tf*NCS lysate desalted into 50 mM HEPES (pH 7.5), using a SephadexTM G-25 in PD-10 column (GE Healthcare lifesciences), were combined with HEPES (50 mM pH 7.5) containing PLP 0.5 mM to a total volume of 5 mL. The reaction was stirred at 37 °C until completion. Once the reaction had completed, the yellow suspension was centrifuged, and the supernatant used for the next step. ATP (110 mg, 0.20 mmol) and L-methionine (30 mg, 0.20 mmol) were combined in HEPES buffer 50 mM and the pH was adjusted to 7.5. The ATP/L-methionine mixture was added to the supernatant and the methylation step was initiated upon addition of 10% v/v of *Ec*MAT lysate, 2% v/v of *Ec*MTAN lysate, and 10% v/v of *Rn*COMT lysate in a total volume of 10 mL containing 20 mM $MgCl_2$ and 200 mM KCl. The reaction was incubated overnight at 37 °C and 180 rpm. After this time an aliquot was taken to calculate the yield by HPLC against the standard product (see the calibration curve below). The reaction was centrifuged, the supernatant was basified with $NaHCO_3$ and extracted with EtOAc (3 x 10 mL). The organic fractions were collected, evaporated under vacuum and the residue purified using preparative HPLC (method B). Fractions containing the desired product were freeze-dried to give nororientaline (*S*)-6,3'-(OMe)$_2$-**3** as white powder (TFA salt, 7 mg, 34% yield, 64% yield by HPLC). Trace impurities (< 5%) were also identified by NMR corresponding to the regioisomeric product methylated at *O*-7. ^{1}H NMR (700 MHz; CD_3OD) δ = 6.84 (d, *J* = 2.1 Hz, 1H, 2'-H), 6.81 (d, *J* = 8.0 Hz, 1H, 5'-H), 6.78 (s, 1H, 8-H), 6.75 (dd, *J* = 8.0, 2.1 Hz, 1H, 6'-H), 6.68 (s, 1-H, 5-H), 4.64 (dd, *J* = 8.8, 5.6 Hz, 1H, 1-H), 3.85 (s, 3H, 6-OMe), 3.83 (s, 3H, 3'-OMe), 3.49-3.43 (m, 1H, NHCHC*H*H), 3.39 (dd, *J* = 14.6, 5.5 Hz, 1H, NHCHCH*H*), 3.30-3.24 (m, 1H, 3-*H*H), 3.07-3.01 (m, 1H, 4-*H*H), 2.95-3.02 (m, 2H, 3-H*H* and 4-H*H*); ^{13}C NMR (151 MHz; CD_3OD) δ = 149.5, 149.3, 147.4, 146.9, 127.5, 125.0, 123.6, 123.2, 116.8, 114.2, 113.9, 112.6, 57.7, 56.4, 40.8, 25.9; HRMS (ES+) found [M +H]$^+$ 316.1544; $C_{18}H_{22}NO_4$ requires 316.1543; $[\alpha]_D^{25}$ + 2.0 (c 0.1, MeOH).[7]

HPLC method A1 (achiral)

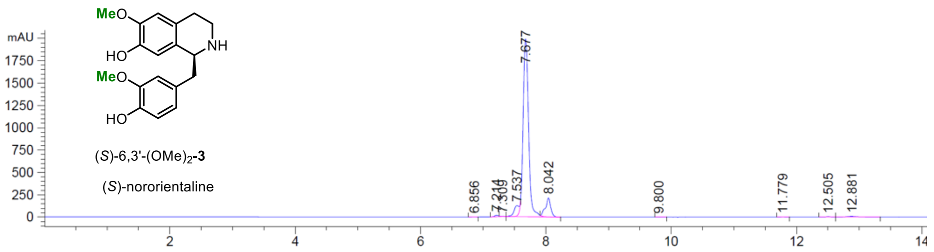

Calibration curve

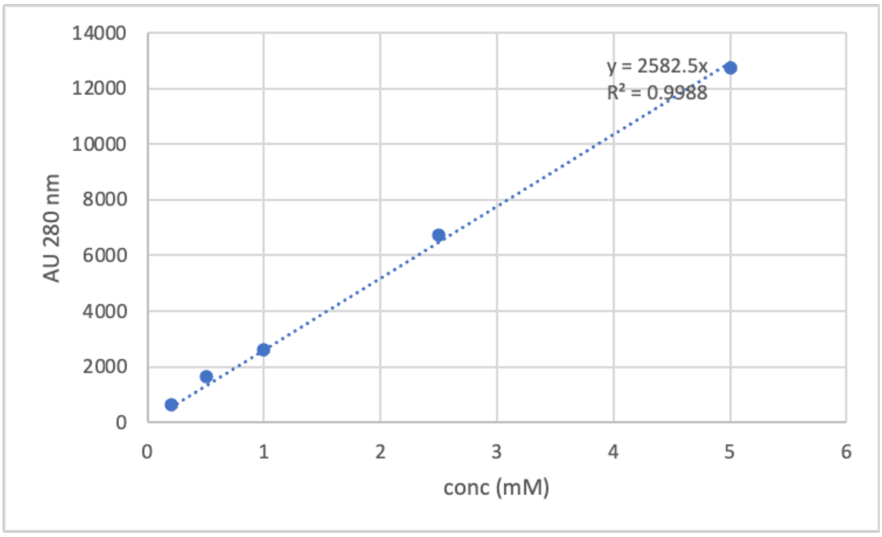

HPLC method C1 (chiral) (*S*)-**3**[4]

e.e. > 97%

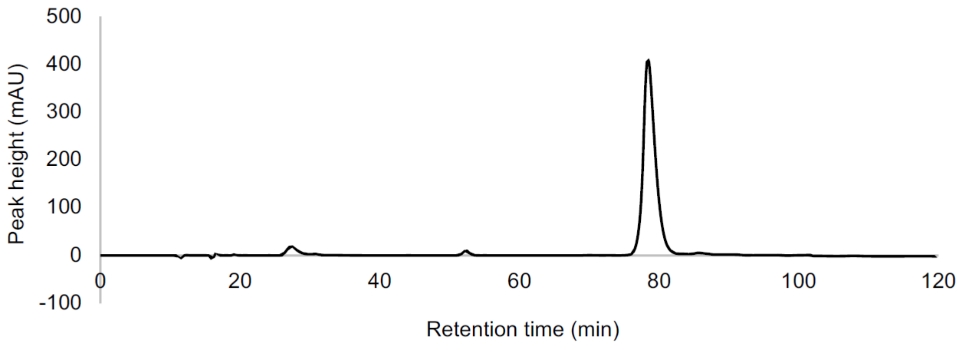

HPLC method C1 (chiral) rac-**3**[4]

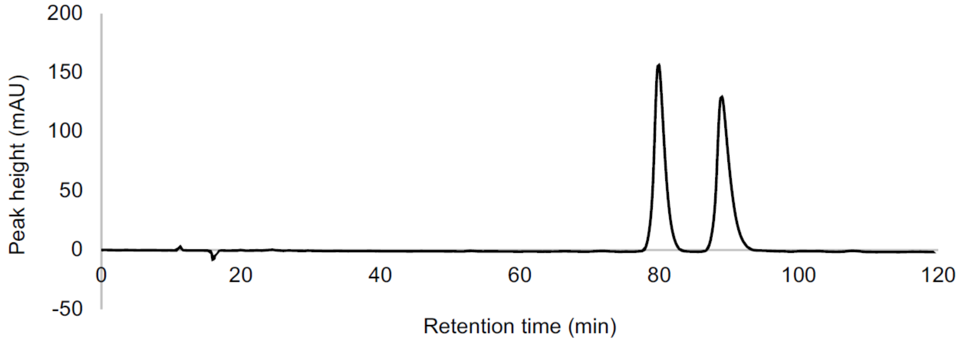

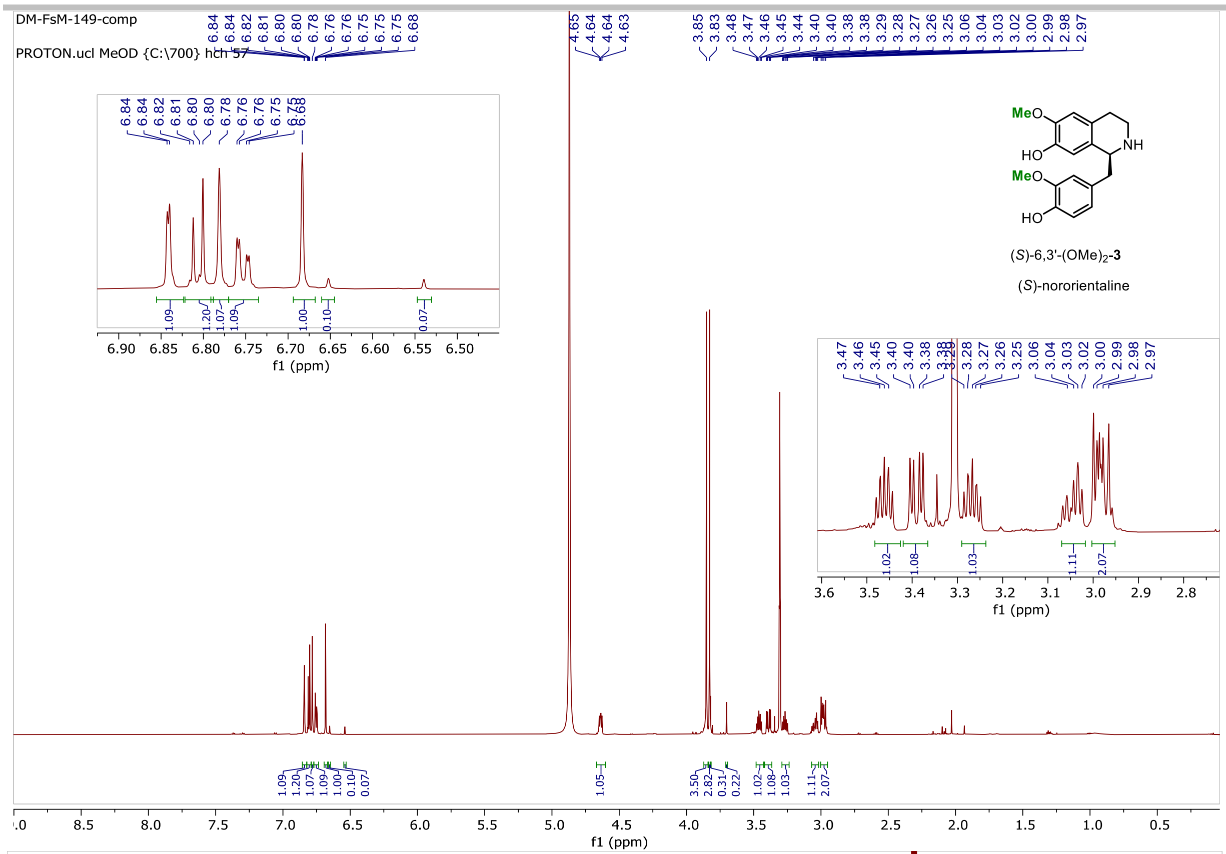
DM-FsM-149-comp
PROTON.ucl MeOD {C:\700} hch 57
6.84 6.84 6.82 6.81 6.80 6.80 6.78 6.76 6.76 6.75 6.75 6.75 6.68
4.65 4.64 4.64 4.63
3.85 3.83 3.48 3.47 3.46 3.45 3.44 3.40 3.40 3.38 3.38 3.29 3.28 3.27 3.26 3.25 3.06 3.04 3.03 3.02 3.00 2.99 2.98 2.97
MeO
HO
NH
MeO
HO
(S)-6,3'-(OMe)2-3
(S)-nororientaline
1.09 1.20 1.07 1.09 1.00 0.10 0.07
1.05
3.50 2.82 0.31 0.22
1.02 1.08 1.03
1.11 2.07
f1 (ppm)

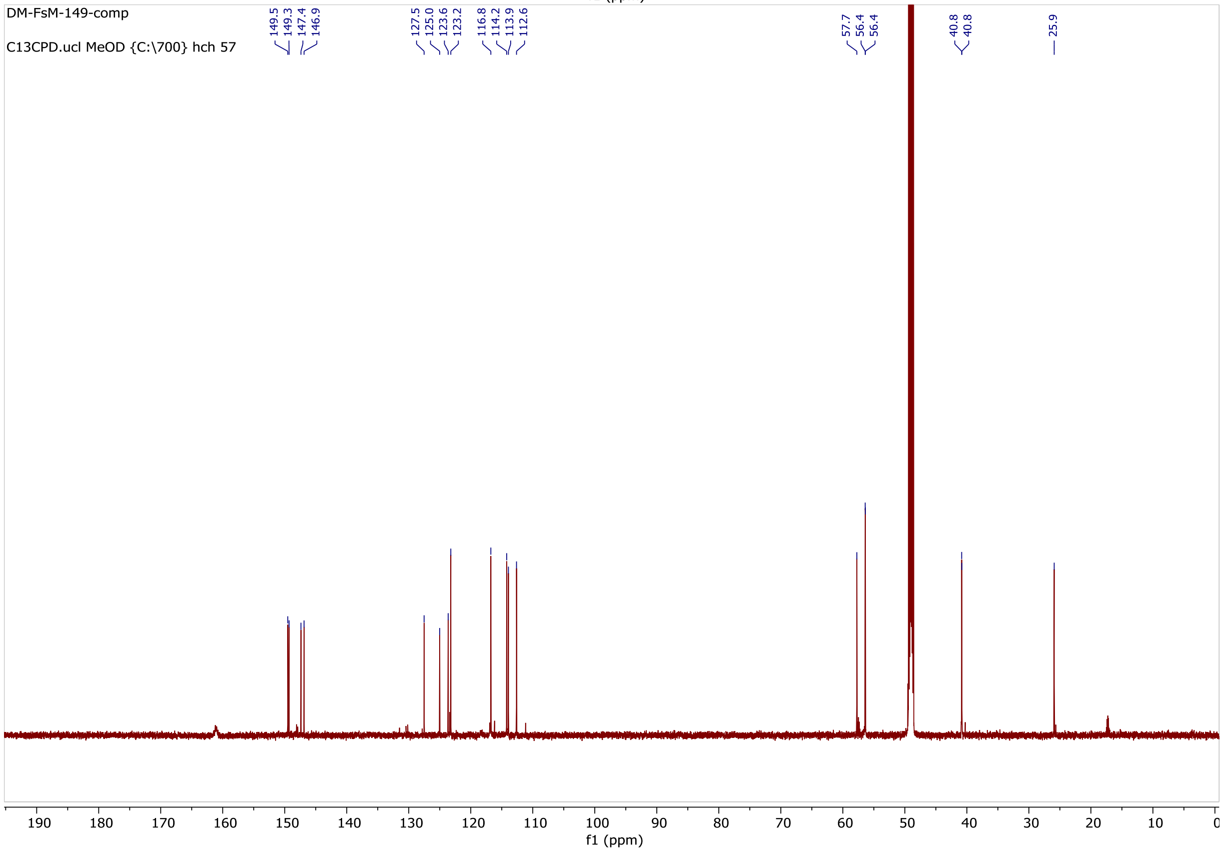
DM-FsM-149-comp
C13CPD.ucl MeOD {C:\700} hch 57
149.5 149.3 147.4 146.9
127.5 125.0 123.6 123.2
116.8 114.2 113.9 112.6
57.7 56.4 56.4
40.8 40.8
25.9
f1 (ppm)

Six-Enzymes Cascade to (*S*)-1-(4-hydroxy-3-methoxybenzyl)-6-methoxy-2-methyl-1,2,3,4-tetrahydroisoquinolin-7-ol (*S*)-2-NMe-6,3'-(OMe)$_2$-3

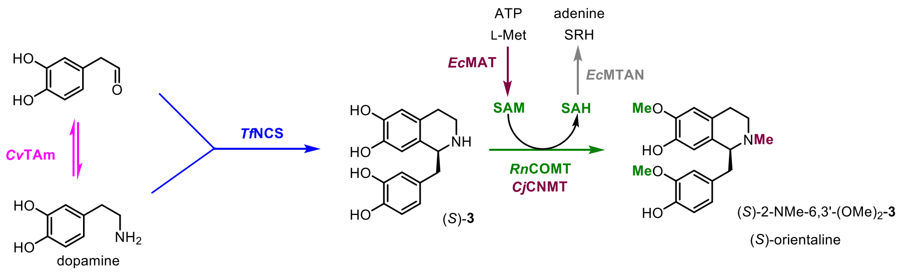

Dopamine hydrochloride (19 mg, 0.1 mmol), sodium ascorbate (20 mg, 0.1 mmol), sodium pyruvate (5.5 mg, 0.05 mmol), *Cv*TAm 10% v/v lysate, and 10% v/v of *Tf*NCS lysate desalted into 50 mM HEPES (pH 7.5), using a SephadexTM G-25 in PD-10 column (GE Healthcare lifesciences), were combined with HEPES (50 mM pH 7.5) containing PLP 0.5 mM to a total volume of 5 mL. The reaction was stirred at 37 °C until completion. Once the reaction had completed, the yellow suspension was centrifuged, and the supernatant used for the next step. ATP (110 mg, 0.20 mmol) and L-methionine (30 mg, 0.20 mmol) were combined in HEPES buffer 50 mM and the pH was adjusted to 7.5. The ATP/L-methionine mixture was added to the supernatant and the methylation step was initiated upon addition of 10% v/v of *Ec*MAT lysate, 2% v/v of *Ec*MTAN lysate, and 10% v/v of *Rn*COMT lysate, and *Cj*CNMT (5 mg of lyophilized pure protein) in a total volume of 10 mL containing 20 mM $MgCl_2$ and 200 mM KCl. The reaction was incubated overnight at 37 °C and 180 rpm. After this time the reaction was centrifuged, the supernatant was basified with $NaHCO_3$ and extracted with EtOAc (3 x 10 mL). The organic fractions were collected, evaporated under vacuum and the residue purified using preparative HPLC (method B). Fractions containing the desired product were freeze-dried to give orientaline (*S*)-2-NMe-6,3'-(OMe)$_2$-**3** as white powder (TFA salt, 7.9 mg, 38% yield). Trace impurities (< 5%) were also identified by NMR corresponding to the regioisomeric product methylated at *O*-7. HRMS (ES+) found $[M+H]^+$ 330.1700; $C_{19}H_{24}NO_4$ requires 330.1700; $[\alpha]_D^{25}$ +45.0 (c 0.24, MeOH).[8]

The product was characterized as a free base and only signal for the major regioisomer are given:

^{1}H NMR (700 MHz, CD_3OD) δ 6.50 (s, 1H, 5-H), 6.78 (d, *J* = 7.8 Hz, 1H, 5'-H), 6.68 (m, 2H, 2'-H and 6'-H), 6.28 (s, 1H, 8-H), 4.55 (t, *J* = 7.0 Hz, 1H, 1-H), 3.86 (s, 3H, 6-OMe), 3.78 (s, 3H, 3'-OMe), 2.97 – 2.90 (m, 1H, 3-H), 2.93 (dd, J = 14.8, 6.2 Hz, 1H, NHCHC*H*H), 2.89 (dd, *J* = 14.8, 4.7 Hz, 1H, NHCHCH*H*), 2.71 (ddd, *J* = 15.4, 9.2, 5.1 Hz, 1H, 4-H), 2.52 (dt, *J* = 12.0, 5.1 Hz, 1H, 3-H), 2.47 (dt, *J* = 15.4, 4.8 Hz, 1H, 4-H), 2.35 (s, 3H); ^{13}C NMR (176 MHz, CD_3OD) δ 162.9, 156.0, 151.7, 151.1, 131.5, 126.2, 123.3, 119.1, 118.6, 118.2, 113.4, 111.4, 65.9, 2 x 55.8, 48.3, 42.9, 42.2, 26.3.

HPLC method A1 (achiral)

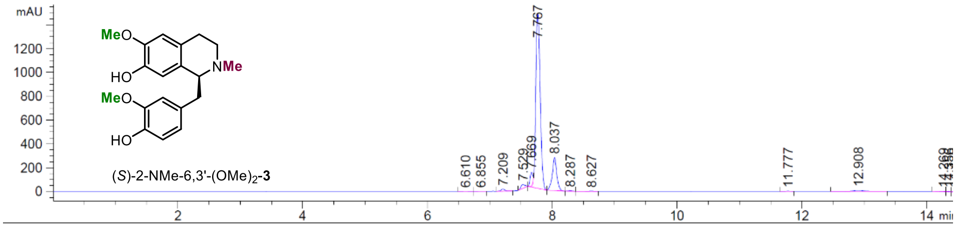

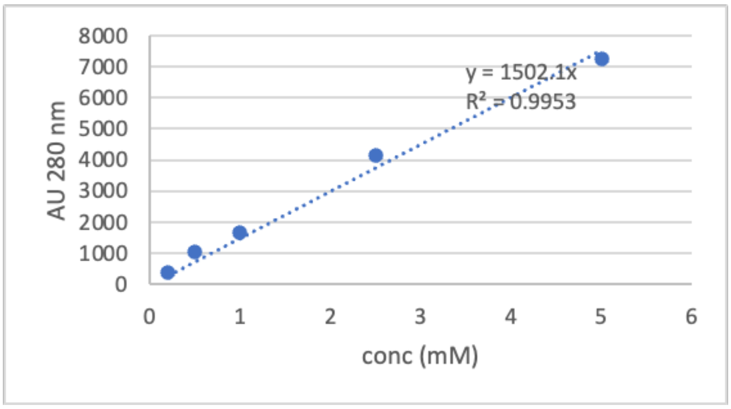

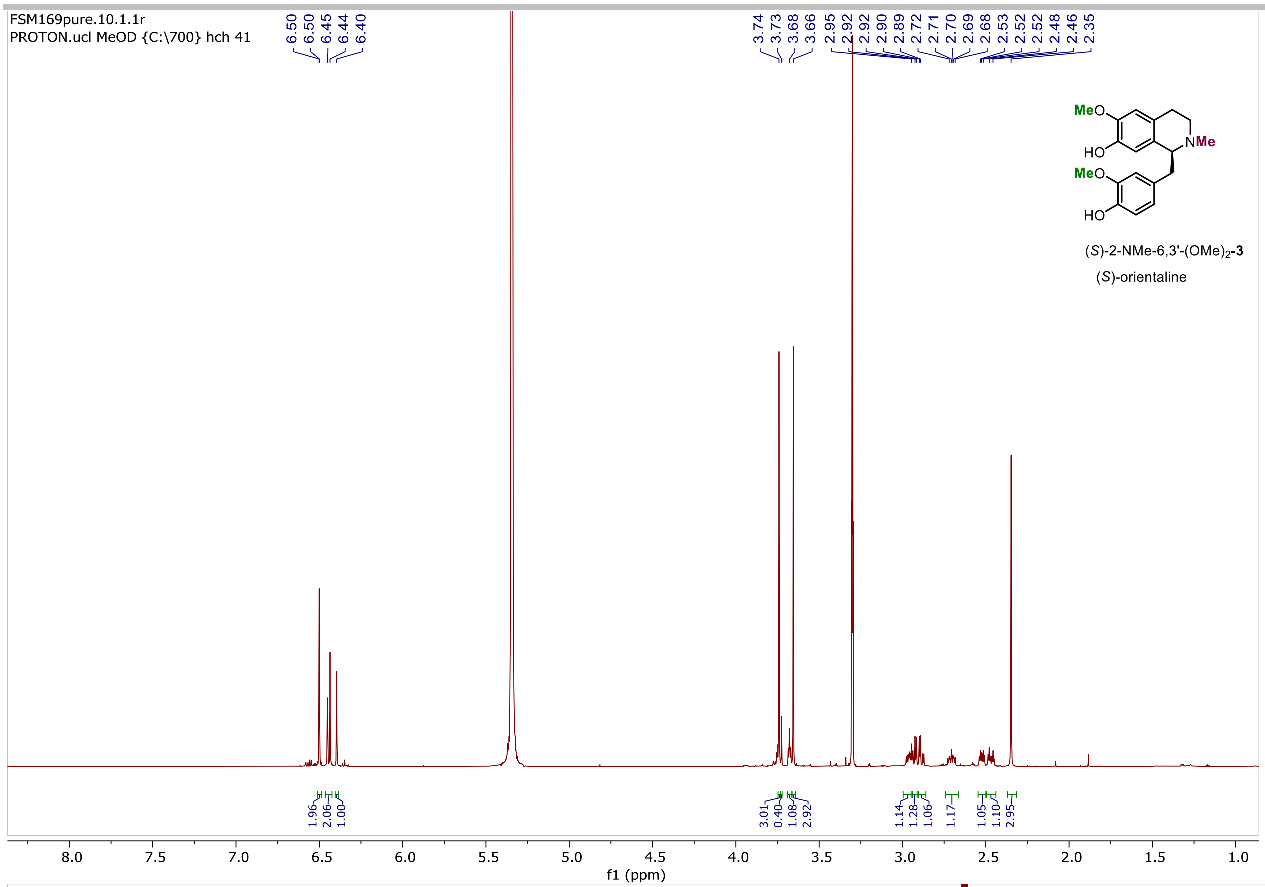
FSM169pure.10.1.1r
PROTON.ucl MeOD {C:\700} hch 41
6.50 6.50 6.45 6.44 6.40
3.74 3.73 3.68 3.66 2.95 2.92 2.92 2.90 2.89 2.72 2.71 2.70 2.69 2.68 2.53 2.52 2.52 2.48 2.46 2.35
MeO
HO
NMe
MeO
HO
(S)-2-NMe-6,3'-(OMe)2-3
(S)-orientaline
1.96 2.06 1.00
3.01 0.40 1.08 2.92
1.14 1.28 1.06
1.17
1.05 1.10 2.95
8.0 7.5 7.0 6.5 6.0 5.5 5.0 4.5 4.0 3.5 3.0 2.5 2.0 1.5 1.0
f1 (ppm)

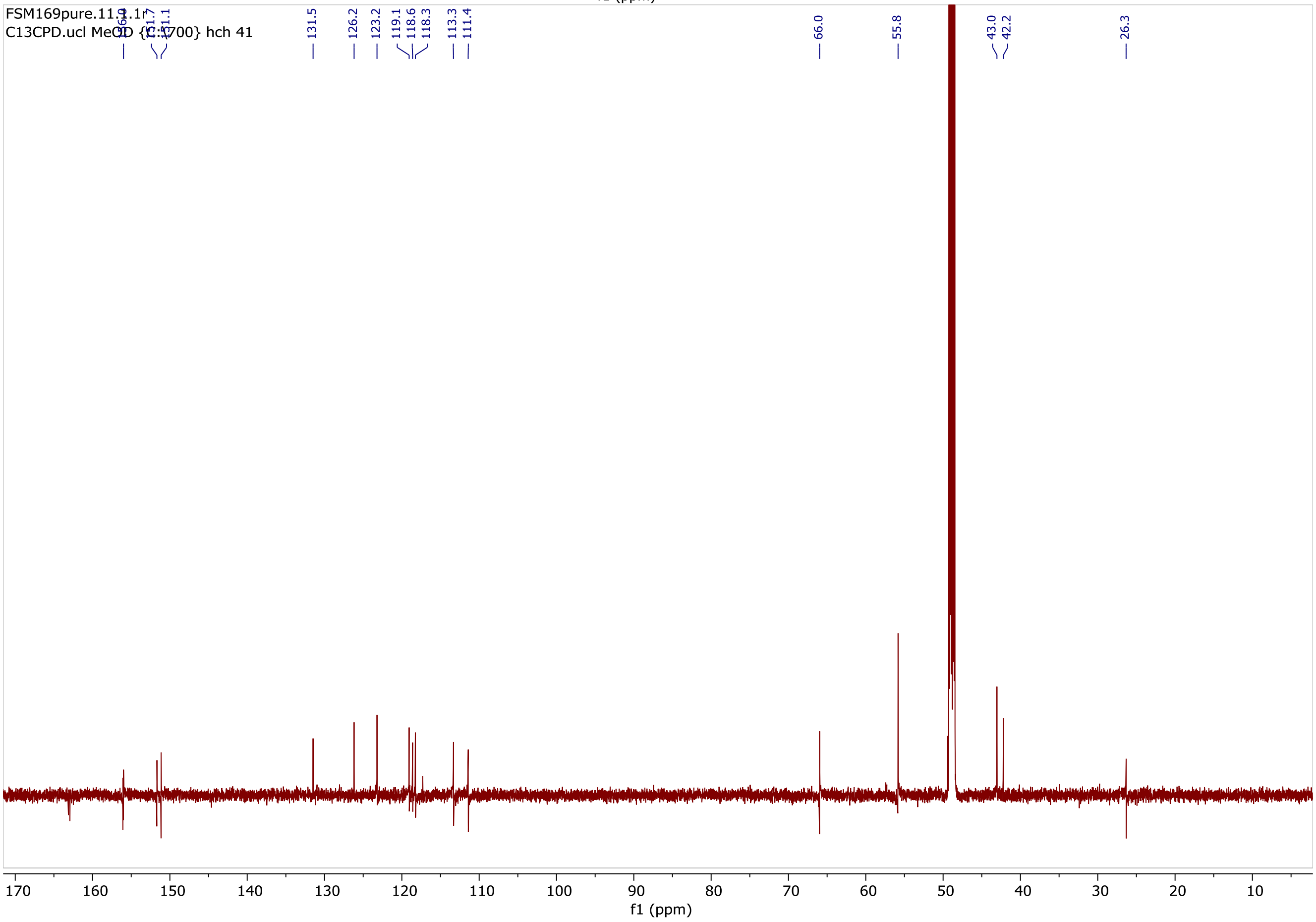
C13CPD.ucl MeOD hch 41
131.5 126.2 123.2 119.1 118.6 118.3 113.3 111.4
66.0 55.8 43.0 42.2 26.3
170 160 150 140 130 120 110 100 90 80 70 60 50 40 30 20 10
f1 (ppm)

Five-Enzymes Cascade to (*S*)-1-(4-hydroxy-3-methoxybenzyl)-6-methoxy-1,2,3,4-tetrahydroisoquinolin-7-ol (*S*)-6,4'-(OMe)$_2$-3

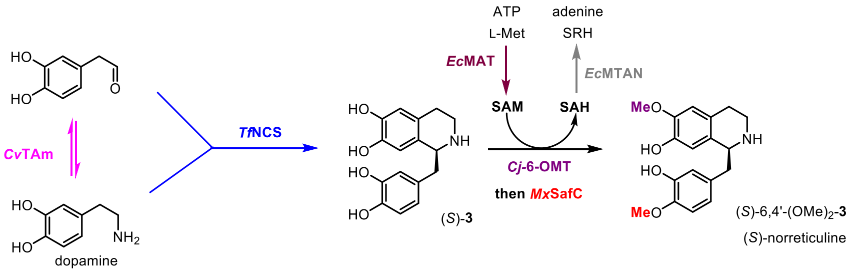

Dopamine hydrochloride (19 mg, 0.1 mmol), sodium ascorbate (20 mg, 0.1 mmol), sodium pyruvate (5.5 mg, 0.05 mmol), *Cv*TAm 10% v/v lysate, and 10% v/v of *Tf*NCS lysate desalted into 50 mM HEPES (pH 7.5), using a SephadexTM G-25 in PD-10 column (GE Healthcare lifesciences), were combined with HEPES (50 mM pH 7.5) containing PLP 0.5 mM to a total volume of 5 mL. The reaction was stirred at 37 °C until completion. Once the reaction had completed, the yellow suspension was centrifuged, and the supernatant used for the next step. ATP (55 mg, 0.10 mmol) and L-methionine (15 mg, 0.10 mmol) were combined in HEPES buffer 50 mM and the pH was adjusted to 7.5. The ATP/ L-methionine mixture was added to the supernatant and the methylation step was initiated upon addition of 10% v/v of *Ec*MAT lysate, 2% v/v of *Ec*MTAN lysate, and 10% v/v of *Cj*-6-OMT lysate in a total volume of 10 mL containing 20 mM $MgCl_2$ and 200 mM KCl. The reaction was incubated overnight at 37 °C and 180 rpm. After this time an aliquot was taken to calculate the yield by HPLC against the product standard (see calibration curve below). The reaction was centrifuged and the supernatant used for the next step. A solution of ATP and L-methionine (0.75 mL, 100 mM in HEPES buffer 50 mM with the pH adjusted to 7.5) was added to the supernatant and the methylation step was initiated upon addition of *Ec*MAT lysate (1 mL), *Ec*MTAN lysate (0.2 mL), and *Mx*SafC lysate (1 mL). The reaction was incubated overnight at 37 °C and 180 rpm. After this time the mixture was centrifuged and the supernatant was basified with $NaHCO_3$ and extracted with EtOAc (3 x 10 mL). The organic fractions were collected, evaporated under vacuum and the residue purified using preparative HPLC (method B). Fractions containing the desired product were freeze-dried to give norreticuline (*S*)-6,4'-(OMe)$_2$-**3** as white powder (TFA salt, 5.6 mg, 27% yield, 70% yield by HPLC). ^{1}H NMR (700 MHz; CD_3OD) δ = 6.93 (d, *J* = 8.2 Hz, 1H, 5'-H), 6.80 (d, *J* = 2.2 Hz, 1H, 2'-H), 6.78 (s, 1H, 5-H), 6.75 (dd, *J* = 8.2, 2.2 Hz, 1H, 6'-H), 6.68 (s, 1H, 8-H), 4.61 (dd, *J* = 9.1, 5.5 Hz, 1H, 1-H), 3.86 (s, 3H, 6-OMe), 3.83 (s, 3H, 4'-OMe), 3.48 (ddd, *J* = 12.5, 6.8, 5.6 Hz, 1H, 3-H*H*), 3.37 (dd, *J* = 14.6, 5.5 Hz, 1H, 4-H*H*), 3.27 (ddd, *J* = 12.8, 7.6, 5.7 Hz, 3-*H*H), 3.10-3.03 (m, 1H, NHCHC*H*H), 2.98 (dt, *J* = 17.1, 6.2 Hz, 1H, NHCHC*H*H), 2.94 (dd, *J* = 14.6, 9.2 Hz, 1H, 4-H*H*); ^{13}C NMR (151 MHz; CD_3OD) δ = 149.2, 148.7, 148.2, 146.8, 128.9, 124.9, 123.4, 121.6, 117.1, 113.9, 113.1, 112.5, 57.7, 56.3 x 2, 40.8, 40.5, 25.8; HRMS (ES+) found $[M+H]^+$ 316.1542; $C_{18}H_{22}NO_4$ requires 316.1543. $[\alpha]_D^{25}$ -27.4 (c 0.25, H_2O).[9]

HPLC method A1 (achiral)

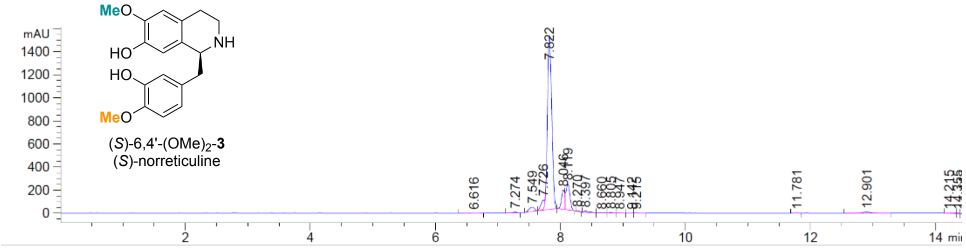

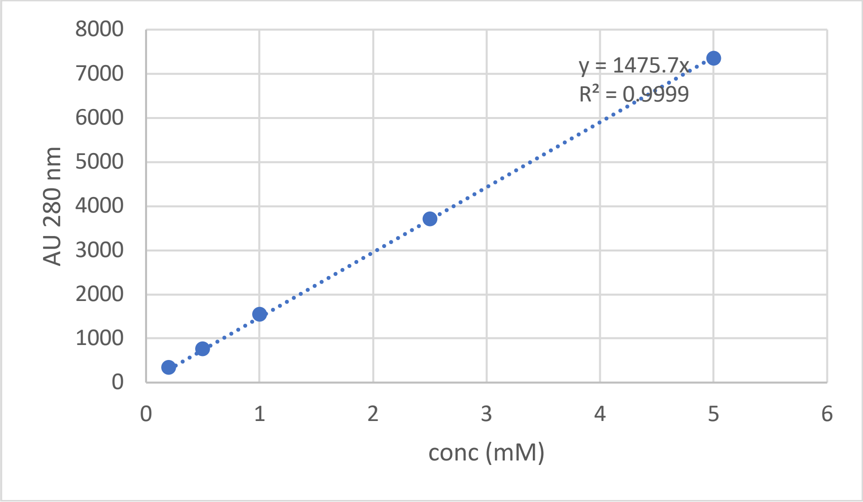

HPLC method C1 (chiral) (*S*)-6,4'-(OMe)$_2$-**3**

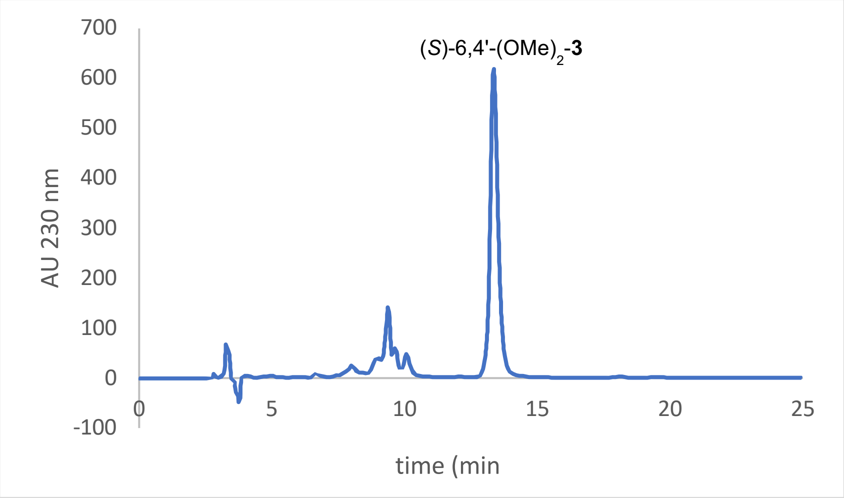

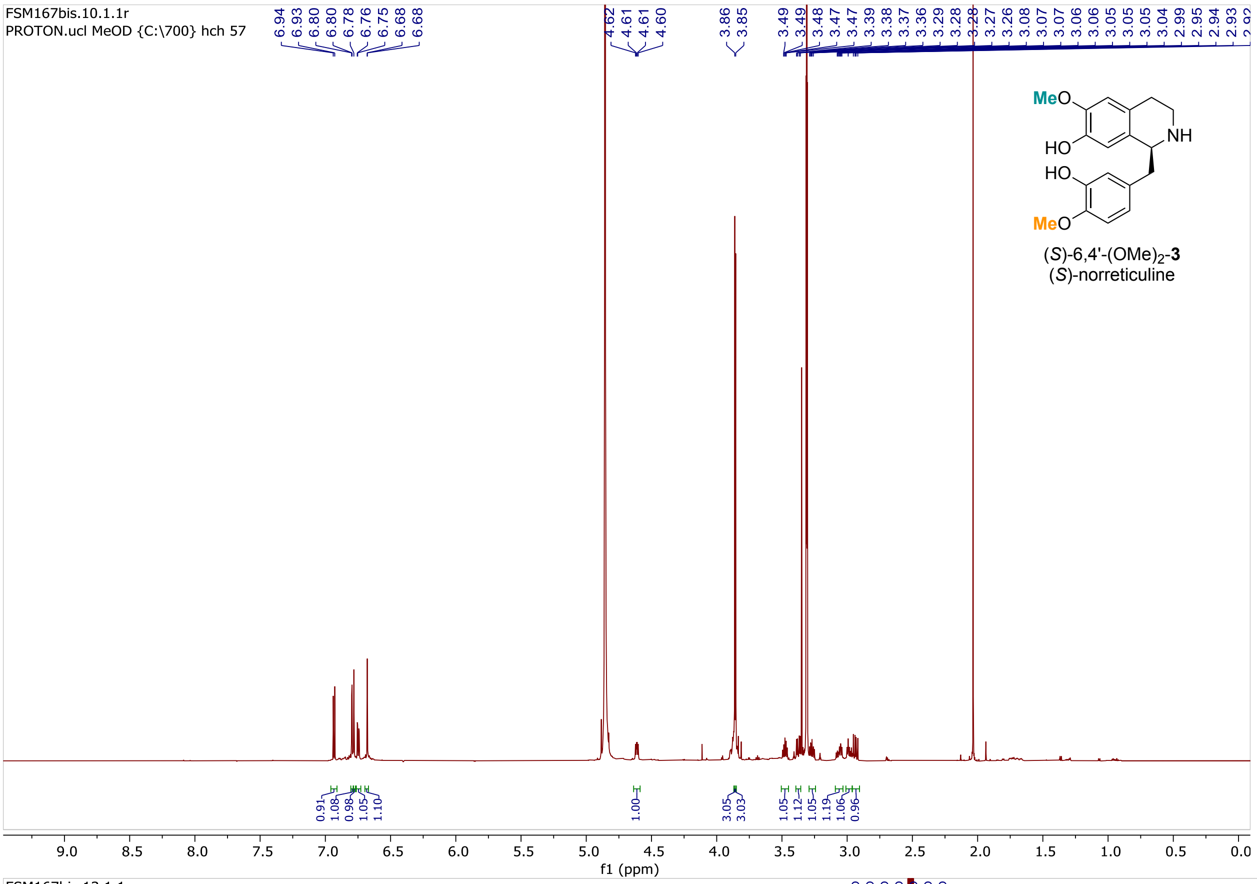
FSM167bis.10.1.1r
PROTON.ucl MeOD {C:\700} hch 57
6.94
6.93
6.80
6.80
6.78
6.76
6.75
6.68
6.68
4.62
4.61
4.61
4.60
3.86
3.85
0.91
1.08
0.98
1.05
1.10
1.00
3.05
3.03
1.05
1.12
1.05
1.19
1.06
0.96
MeO
HO
NH
HO
MeO
(S)-6,4'-(OMe)2-3
(S)-norreticuline
f1 (ppm)

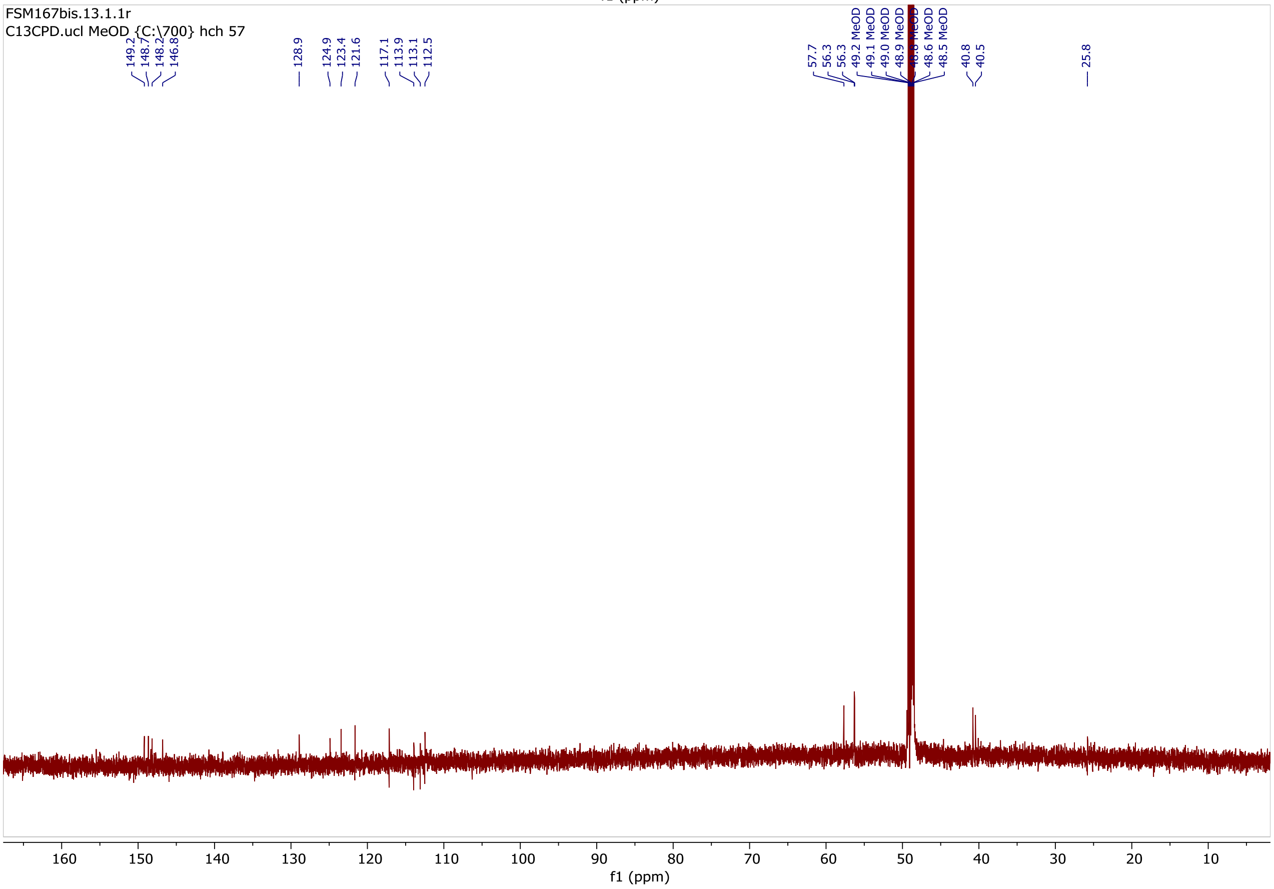
FSM167bis.13.1.1r
C13CPD.ucl MeOD {C:\700} hch 57
149.2
148.7
148.2
146.8
128.9
124.9
123.4
121.6
117.1
113.9
113.1
112.5
57.7
56.3
56.3
49.2 MeOD
49.1 MeOD
49.0 MeOD
48.9 MeOD
48.6 MeOD
48.5 MeOD
40.8
40.5
25.8
f1 (ppm)

Six-Enzymes Cascade to (*S*)-1-benzyl-8-fluoro-6-methoxy-1,2,3,4-THIQ-7-ol (*S*)-6-OMe-5

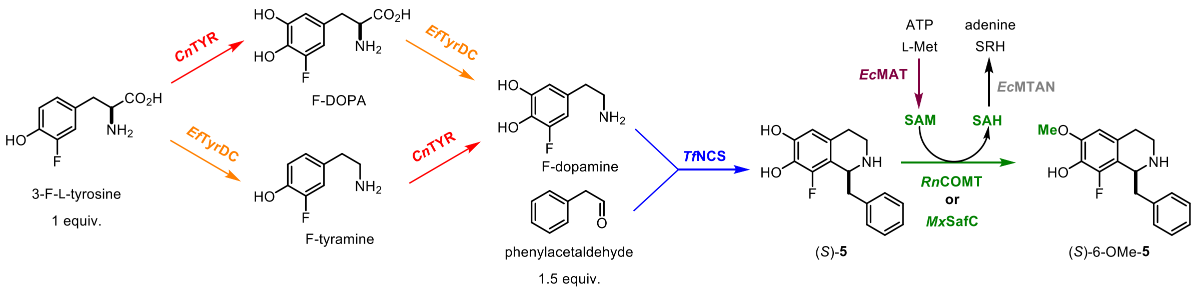

The reaction mixture (20 mL, pH 5.5) consisted of 50 mM HEPES, 10 mM 3-F-L-tyrosine, 40 mM sodium ascorbate, 100 µM $CuSO_4 \cdot 5H_2O$ and 5 mM PLP. To initiate the hydroxylase and decarboxylation step, 10% v/v of *Cn*TYR and *Ef*TyrDC lysate were added to the reaction mixture which was incubated at 25 °C, 250 rpm for 16 h. After this time, the pH was adjusted to 7.5 with 2.5 M NaOH, and 15 mM phenylacetaldehyde were added. The Pictet-Spengler reaction was initiated by adding 50 µg/mL of pure *Tf*NCS and the reaction was incubated at 37 °C, 250 rpm for 8 h. The reaction mixture was then centrifuged, and 40 mM $MgCl_2$, 200 mM KCl, 20 mM ATP and 20 mM L-methionine were added to the supernatant. To initiate the methylation step, 10% v/v of *Rn*COMT lysate, 10% v/v of *Ec*MAT lysate, and 2.5% v/v of *Ec*MTAN lysate were added to the reaction mixture which was incubated at 37 °C, 250 rpm for another 8 h. After this time the reaction was centrifuged, and the supernatant was extracted with EtOAc (3 x 20 mL). The organic fractions were collected, evaporated under vacuum, and the residue purified using preparative HPLC (method B). Fractions containing the desired product were freeze-dried to give (*S*)-6-OMe-**5** as an off-white powder (TFA salt, 14.6 mg, 19%, 27% yield by HPLC). Trace impurities (5 %) were also identified by NMR corresponding to the regioisomeric product methylated at *O*-7. ^{1}H NMR (700 MHz; CD_3OD) δ = 7.39 (t, *J* = 7.4 Hz, 2H, 3'-H and 5'-H), 7.34-7.31 (m, 3H, 2'-H, 4'-H, and 6'-H), 6.69 (s, 1H, 5-H), 4.94 (dd, *J* = 9.5, 4.5 Hz, 1H, 1-H), 3.88 (s, 3H, OMe), 3.53 (dt, *J* = 13.0, 7.5 Hz, 1H, 3-*H*H), 3.43 (dd, *J* = 15.0, 4.5 Hz, 1H, NHCHC*H*H), 3.35-3.32 (m, 1H, 3-H*H*), 3.18 (dd, *J* = 15.0, 9.5 Hz, 1H, NHCHCH*H*), 3.04 (dd, *J* =7.5, 5.1 Hz, 2H, 4-H); ^{13}C NMR (151 MHz; CD_3OD) δ = 151.1, 151.0, 150.3, 148.8, 136.4, 135.0, 130.5, 130.3, 128.9, 108.2, 56.8, 53.3, 39.9, 38.8, 25.4; HRMS (ES+) found $[M+H]^+$ 288.1394; $C_{17}H_{19}FNO_2$ requires 288.1394; $[\alpha]_D^{26}$ + 2.0 (c 0.2, MeOH).

Compound (S)-6-OMe-**5** was also obtained as an off-white powder (TFA salt, 15.3 mg, 20%, 31% yield by HPLC) using *Mx*SafC instead of *Rn*COMT and following the above procedure.

HPLC Method A2 (achiral) (*S*)-6-OMe-**5**

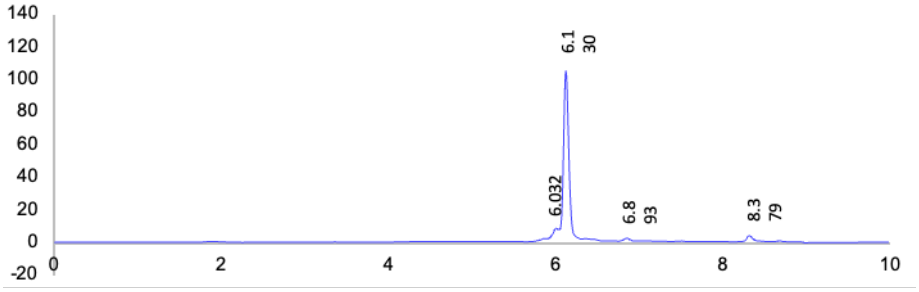

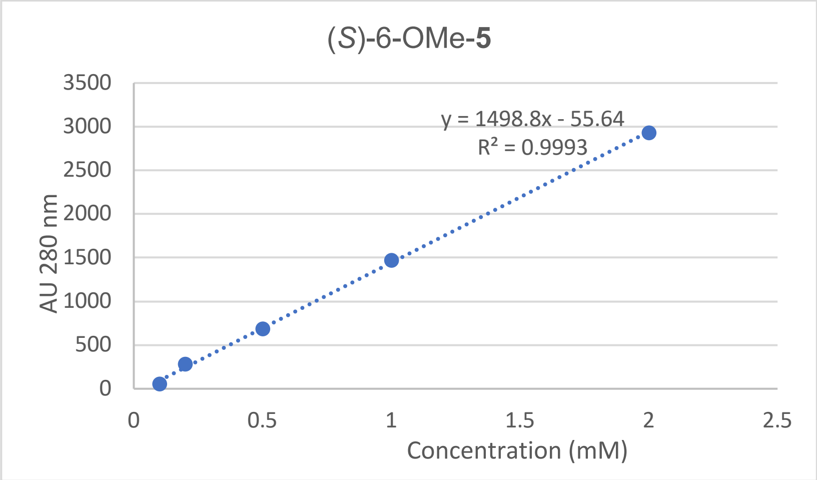

HPLC methd C1 (chiral) (*S*)-**5**[4]

e.e 90%

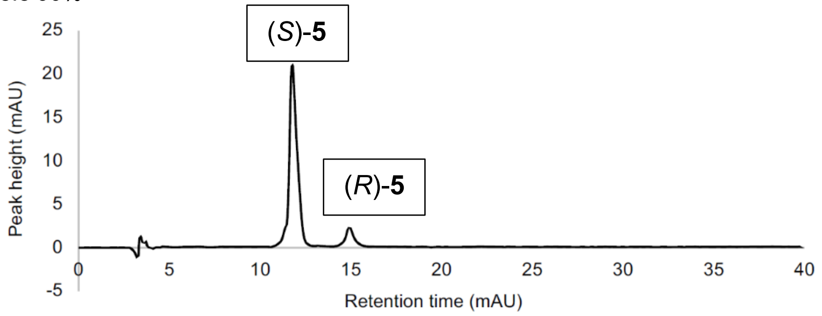

HPLC methd C1 (chiral) rac-**5**[4]

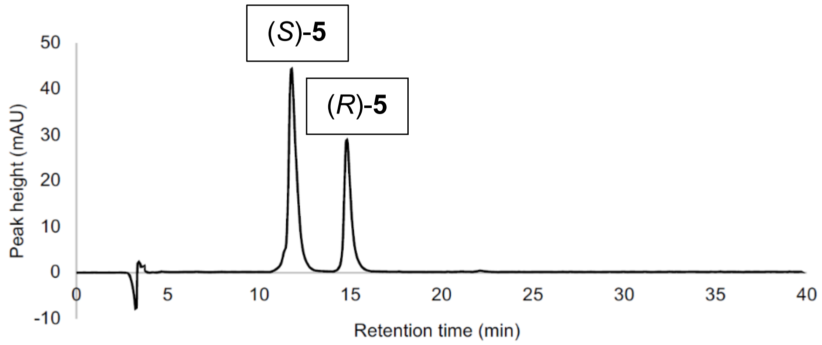

HPLC methd C1 (chiral) (*S*)-6-OMe-**5**

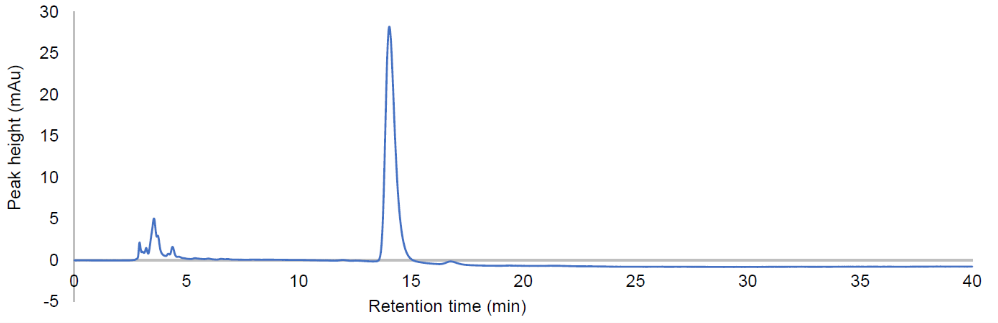

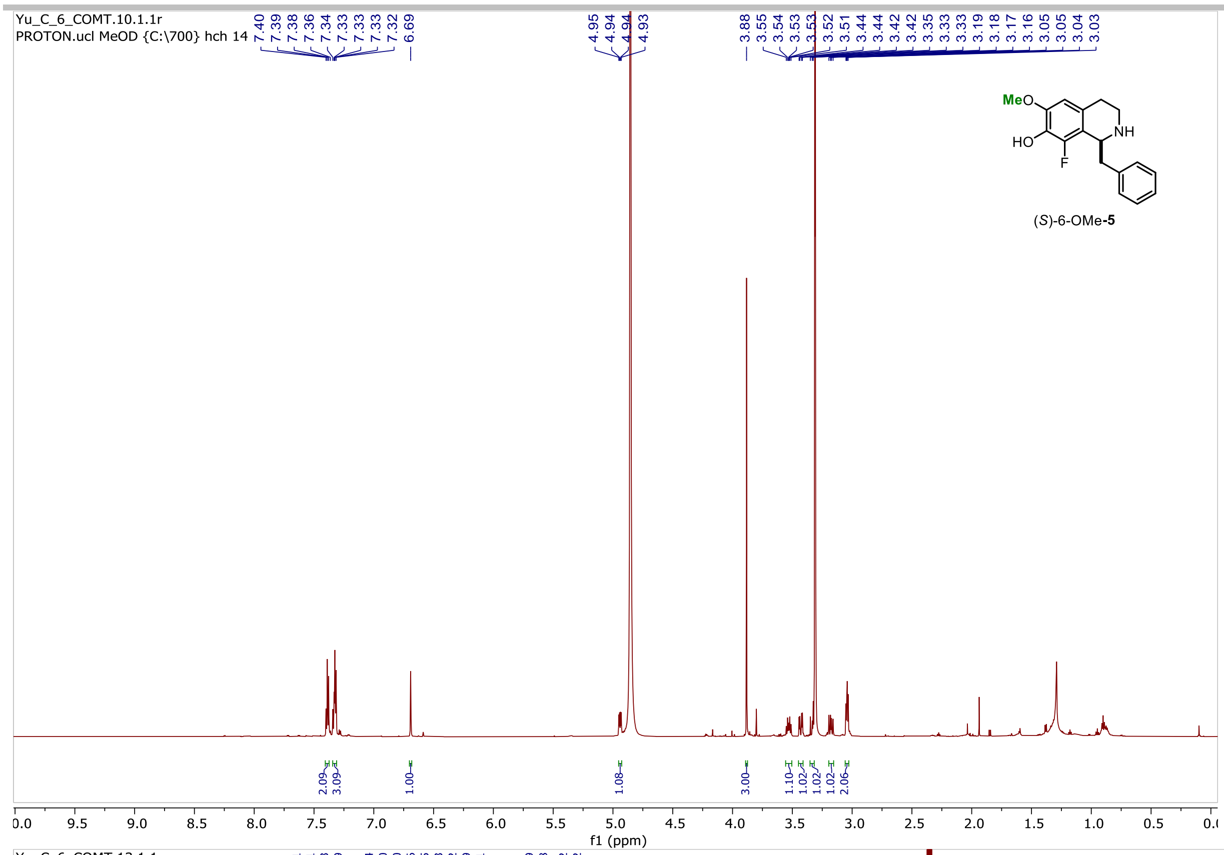
Yu_C_6_COMT.10.1.1r
PROTON.ucl MeOD {C:\700} hch 14
7.40 7.39 7.38 7.36 7.34 7.33 7.33 7.33 7.32 6.69
4.95 4.94 4.94 4.93
3.88 3.55 3.54 3.53 3.53 3.52 3.51 3.44 3.44 3.42 3.42 3.35 3.33 3.33 3.19 3.18 3.17 3.16 3.05 3.05 3.04 3.03
MeO
HO
F
NH
(S)-6-OMe-5
2.09 3.09 1.00 1.08 3.00 1.10 1.02 1.02 1.02 2.06
f1 (ppm)

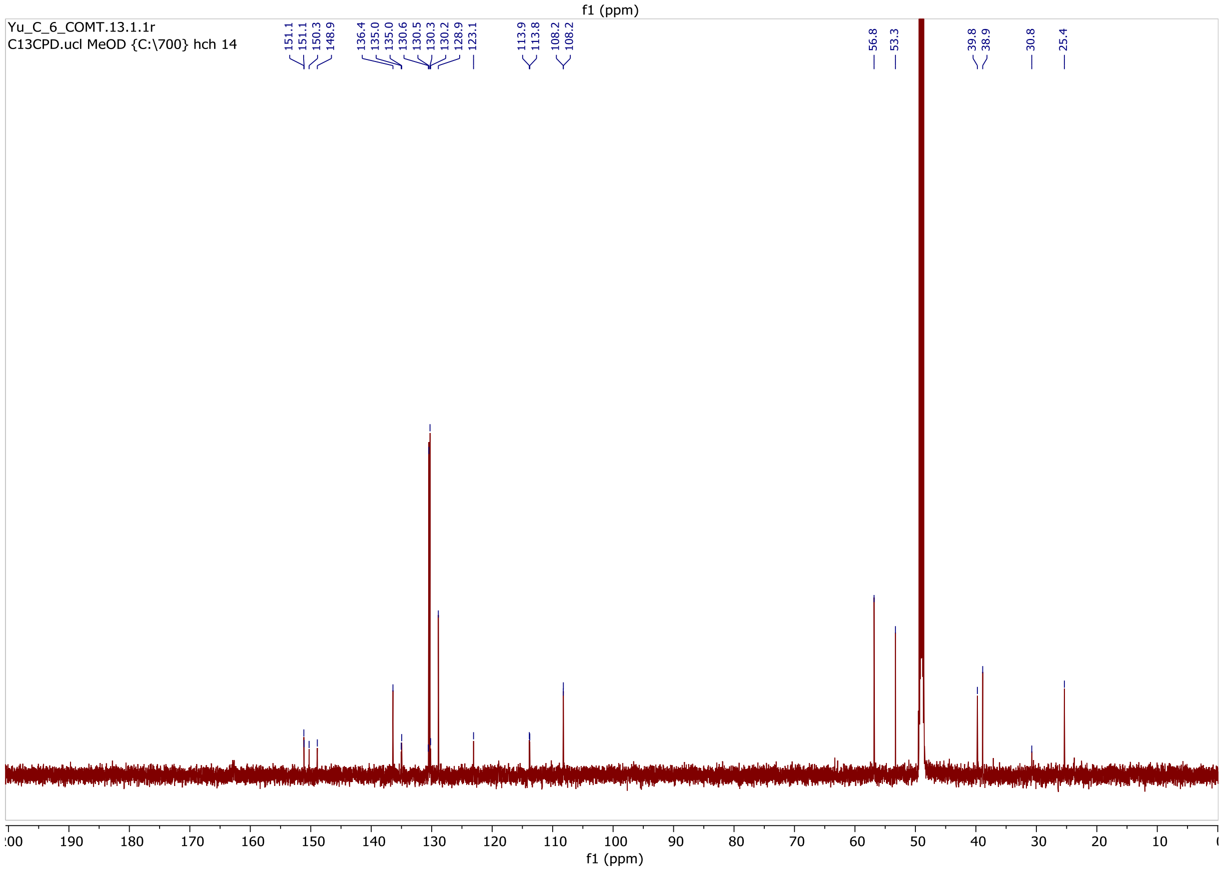
Yu_C_6_COMT.13.1.1r
C13CPD.ucl MeOD {C:\700} hch 14
151.1 151.1 150.3 148.9 136.4 135.0 135.0 130.6 130.5 130.3 130.2 128.9 123.1 113.9 113.8 108.2 108.2 56.8 53.3 39.8 38.9 30.8 25.4
f1 (ppm)

Four-Enzymes Cascade to (*S*)-6-methoxy-1-pentyl-1,2,3,4-tetrahydroisoquinolin-7-ol (*S*)-6-OMe-6

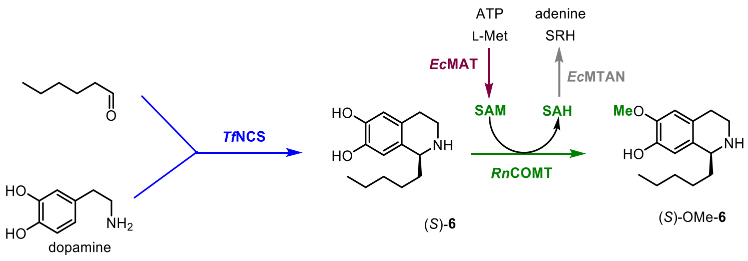

Dopamine hydrochloride (19 mg, 0.1 mmol), hexanal (49.2 µl, 0.4 mmol) dissolved in 1 mL of acetonitrile, sodium ascorbate (19.8 mg, 0.025 mmol), and *Tf*NCS (5 mg) were combined with HEPES (50 mM pH 7.5) mM to a total volume of 10 mL. The reaction was incubated at 37 °C with 200 rpm shaking until completion (3 h). Once the reaction had completed, the suspension was centrifuged, and the supernatant used for the next step. The methylation step was initiated in the same pot and all the components were added to have a final concentration of 5 mM ATP (110.2 mg, 0.2 mmol), 5 mM of L-methionine (30 mg, 0.2 mmol), 10 % v/v of *Ec*MAT lysate, 2% v/v of *Ec*MTAN lysate, and 10% v/v of *Rn*COMT lysate in a total volume of 20 mL containing 20 mM $MgCl_2$ and 200 mM KCl. The reaction was started upon enzymes addition and incubated overnight at 37 °C at 200 rpm. After this time an aliquot was taken to calculate the HPLC yield against the product standard (see calibration curve below). The reaction was quenched with 1 M HCl (2 ml) and the product was purified using preparative HPLC (method B). Fractions containing the desired product were concentrated under vacuum and re-dissolved in 0.1 M aqueous HCl, before lyophilisation to give (*S*)-6-OMe-**6** as a white powder (HCl salt, 12.6 mg, 44 % yield, 90% yield by HPLC). ^{1}H NMR (700 MHz; D_2O) δ = 6.84 (s, 1H, 5-H), 6.75 (s, 1H, 8-H), 4.45 (dd, *J* = 8.3, 4.8 Hz, 1H, 1-H), 3.86 (s, 1H, -OMe), 3.57 (dt, *J* = 12.4, 6.0 Hz, 1H, 3-H), 3.40-3.35 (m, 1H, 3-H); 3.06 (dt, *J* = 17.2, 6.2 Hz, 1H, 4-H), 3.00 (dt, *J* = 17.2, 6.2 Hz, 1H, 4-H), 2.09-2.02 (m, 1H, 1'-H), 1.92-1.81 (m, 1H, 1'-H), 1.49-1.33 (m, 2H, 3'-*HH*), 1.39-1.30 (m, 4H, 4'-*HH* and 2'-*HH*), 0.87 (t, *J* = 7.6 Hz, 3H, -Me); ^{13}C NMR (176 MHz; D_2O - MeOH standard) δ = 147.7, 144.3, 125.3, 124.3, 113.6, 112.9, 56.6, 55.5, 39.9, 33.7, 31.3, 24.8, 24.7, 22.2, 13.7; HRMS (ES+) found $[M+H]^+$ 250.1802; $C_{15}H_{24}NO_2$ requires 250.1802; $[\alpha]_D^{25}$ +13.2 (c 0.07, H_2O).

HPLC Method A1 (achiral)

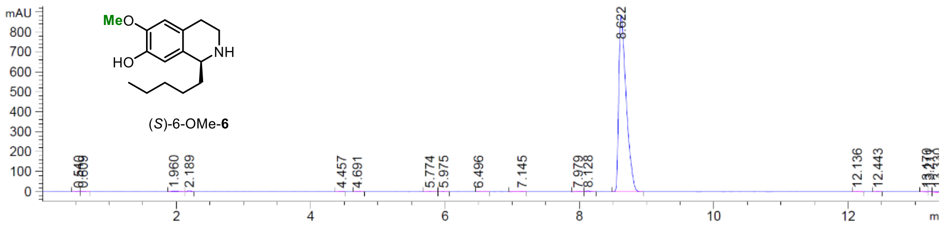

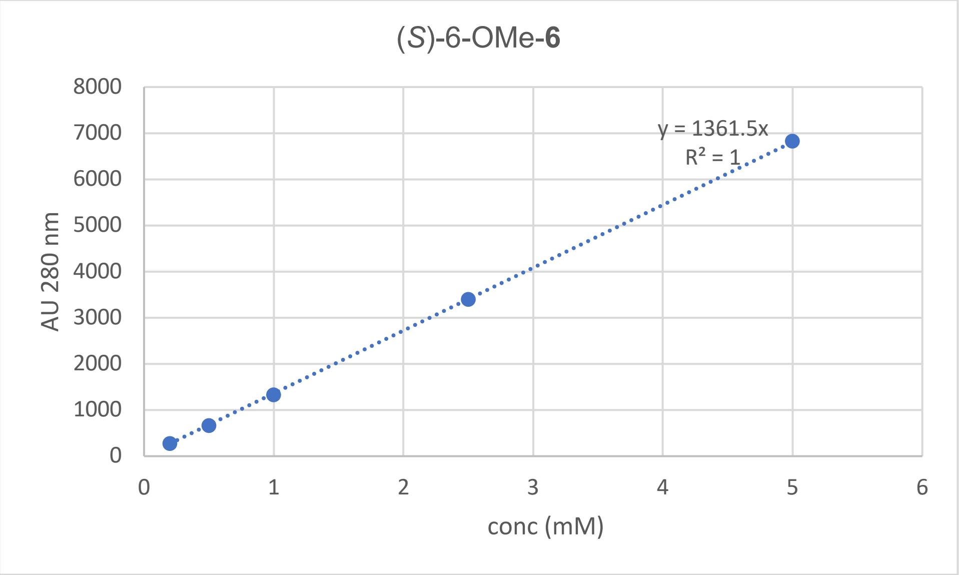

HPLC method C4 (chiral) (*S*)-**6**
e.e. >98%

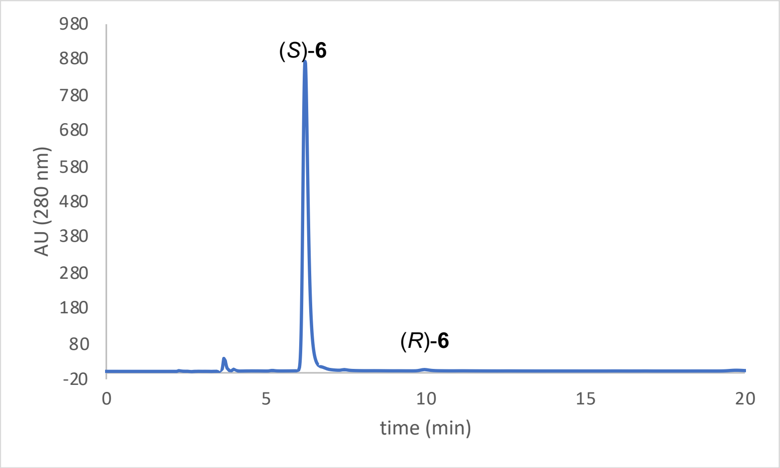

HPLC method C4 (chiral) rac-**6**

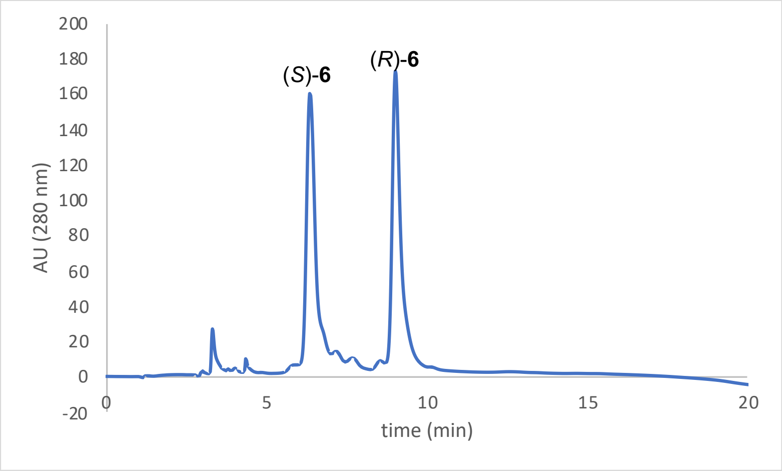

HPLC method C1 (chiral) (*S*)-6-OMe-**6**

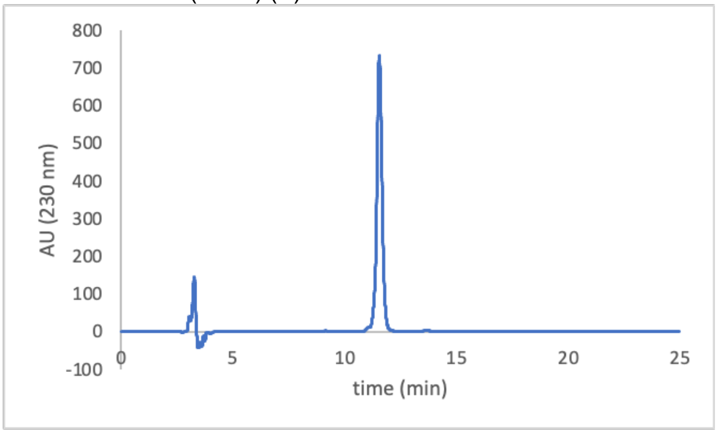

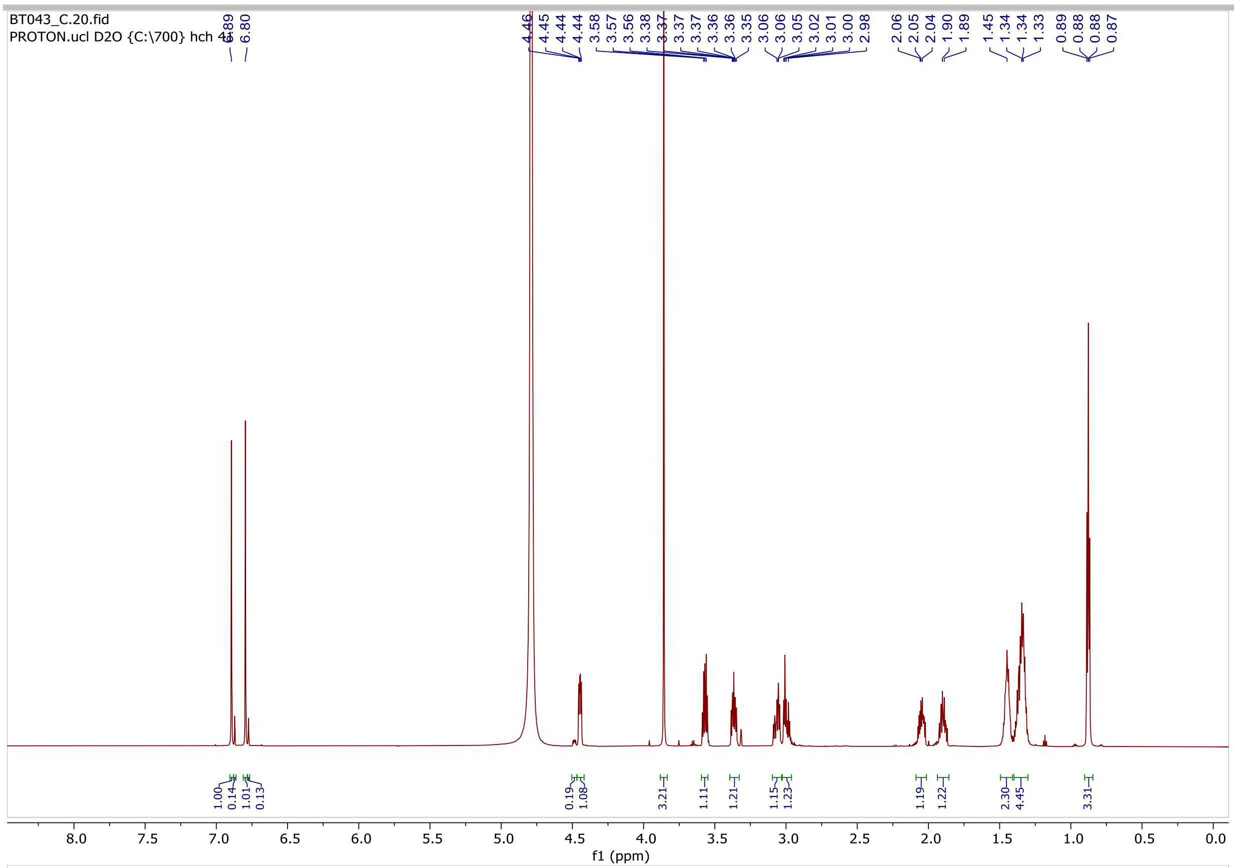
BT043_C.20.fid
PROTON.ucl D2O {C:\700} hch 41
6.89
6.80
4.46
4.45
4.44
4.44
3.58
3.57
3.56
3.38
3.37
3.37
3.37
3.36
3.36
3.35
3.06
3.06
3.05
3.02
3.01
3.00
2.98
2.06
2.05
2.04
1.90
1.89
1.45
1.34
1.34
1.33
0.89
0.88
0.88
0.87
1.00
0.14
1.01
0.13
0.19
1.08
3.21
1.11
1.21
1.15
1.23
1.19
1.22
2.30
4.45
3.31
f1 (ppm)

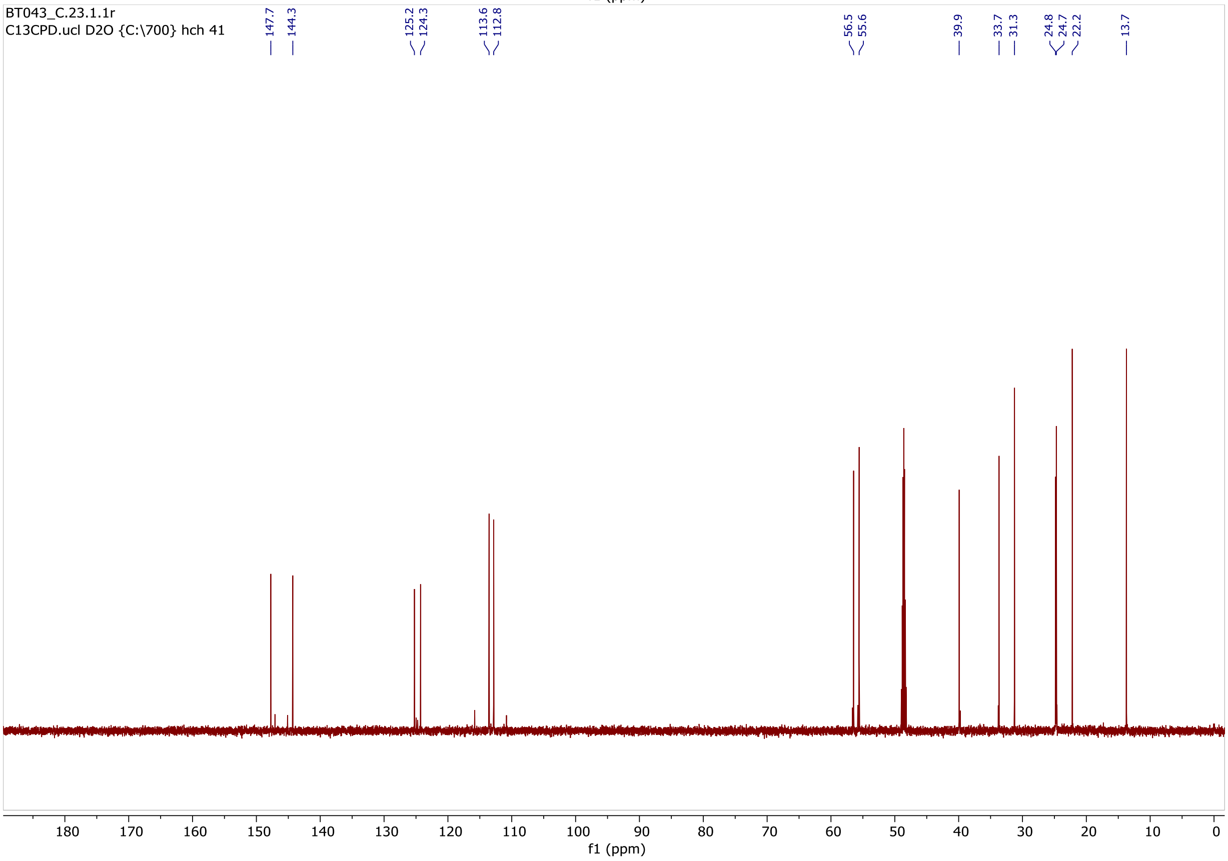
BT043_C.23.1.1r
C13CPD.ucl D2O {C:\700} hch 41
147.7
144.3
125.2
124.3
113.6
112.8
56.5
55.6
39.9
33.7
31.3
24.8
24.7
22.2
13.7
f1 (ppm)

Four-Enzymes Cascade to 7'-methoxy-3',4'-dihydro-2'H-spiro[cyclohexane-1,1'-isoquinolin]-6'-ol 7-OMe-9

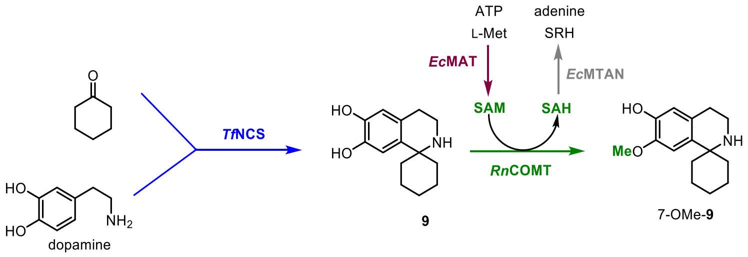

Dopamine hydrochloride (9.5 mg, 0.05 mmol), cyclohexanone (7.5 µL, 0.075 mmol) dissolved in 0.25 mL of acetonitrile, sodium ascorbate (10 mg, 0.05 mmol), and 50% v/v of *Tf*NCS clarified lysate were combined with HEPES (50 mM pH 7.5) to a total volume of 2.5 mL. The reaction was stirred at 37 °C until completion (16 h). Once the reaction had completed, the suspension was centrifuged supernatant used for the next step. ATP (27 mg, 0.05 mmol) and L-methionine (7.5 mg, 0.05 mmol) were combined in HEPES buffer 50 mM and the pH was adjusted to 7.5. The ATP/L-methionine mixture was added to the supernatant and the methylation step was initiated upon addition of 10% v/v of *Ec*MAT lysate, 2% v/v of *Ec*MTAN lysate, and 10% v/v of *Rn*COMT lysate in a total volume of 5 mL containing 20 mM $MgCl_2$ and 200 mM KCl. The reaction was incubated overnight at 37 °C and 180 rpm. After this time the reaction was centrifuged, the supernatant was basified with $NaHCO_3$ and extracted with EtOAc (3 x 10 mL). The organic fractions were collected, evaporated under vacuum and the residue purified using preparative HPLC (method B). Fractions containing the desired product were freeze-dried to give 7-OMe-**9** as white powder (TFA salt, 9.6 mg, 40%). An impurity (10%) was also identified by NMR corresponding to the regioisomeric product methylated at *O*-7. ^{1}H NMR (700 MHz; CD_3OD) δ = 6.89 (s, 1H, 8'-H), 6.63 (s, 1H, 5'-H), 3.87 (s, 3H, OMe), 3.43 (t, *J* = 6.4 Hz, 2H, 3'-H), 2.99 (t, *J* = 6.4 Hz, 2H, 4'-H), 2.15 (td ap, *J* = 14.1, 13.6, 4.3 Hz, 2H, 2x 1-*HH*), 2.10-2.05 (m, 2H, 2 x 1-*HH*), 1.90-1.79 (m, 3H, 2 x 2*HH*+3*H*H), 1.66 (m, 2H, 2 x 2H*H*) – 1.53 (1H, m, 3H*H*); ^{13}C NMR (151 MHz; CD_3OD) δ = 148.7, 147.6, 129.8, 124.6, 116.1, 110.1, 61.3, 56.7, 38.5, 36.8, 36.6, 26.3, 25.1, 21.6; HRMS (ES+) found [M+H]+ 248.1645; $C_{15}H_{21}NO_2$ requires 248.1645.[10]

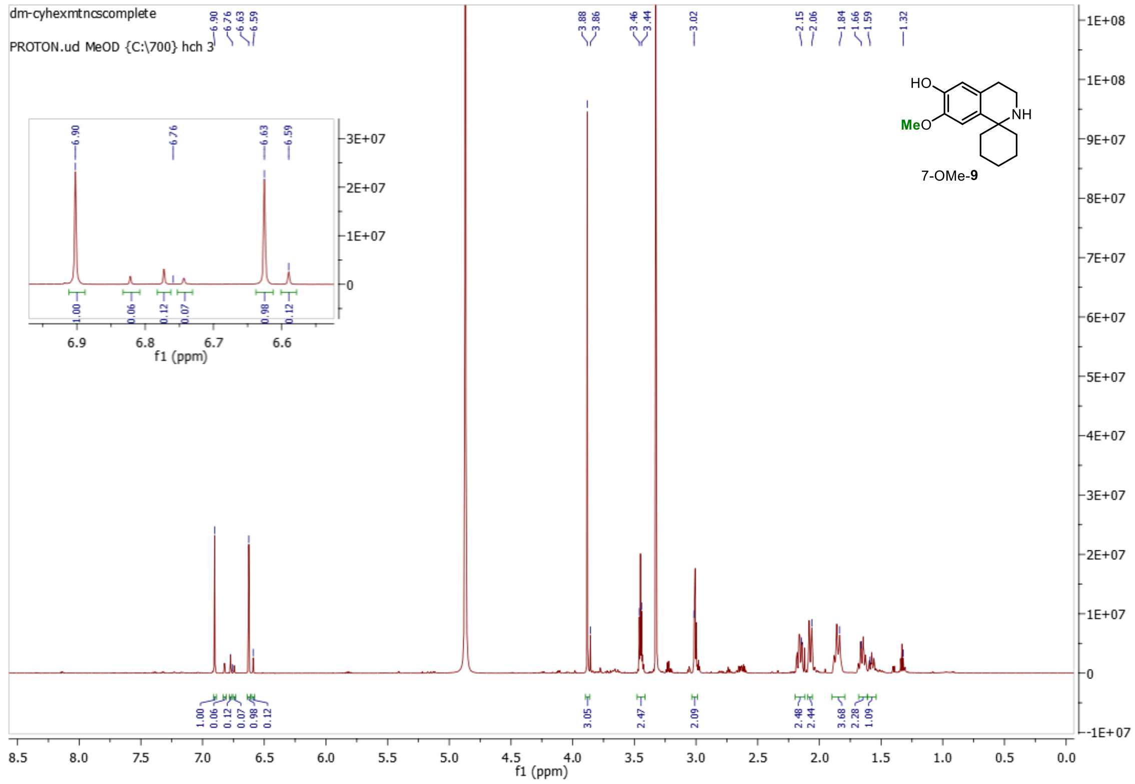
dm-cyhexmtncscomplete
PROTON.ud MeOD {C:\700} hch 3
HO
MeO
NH
7-OMe-9
f1 (ppm)

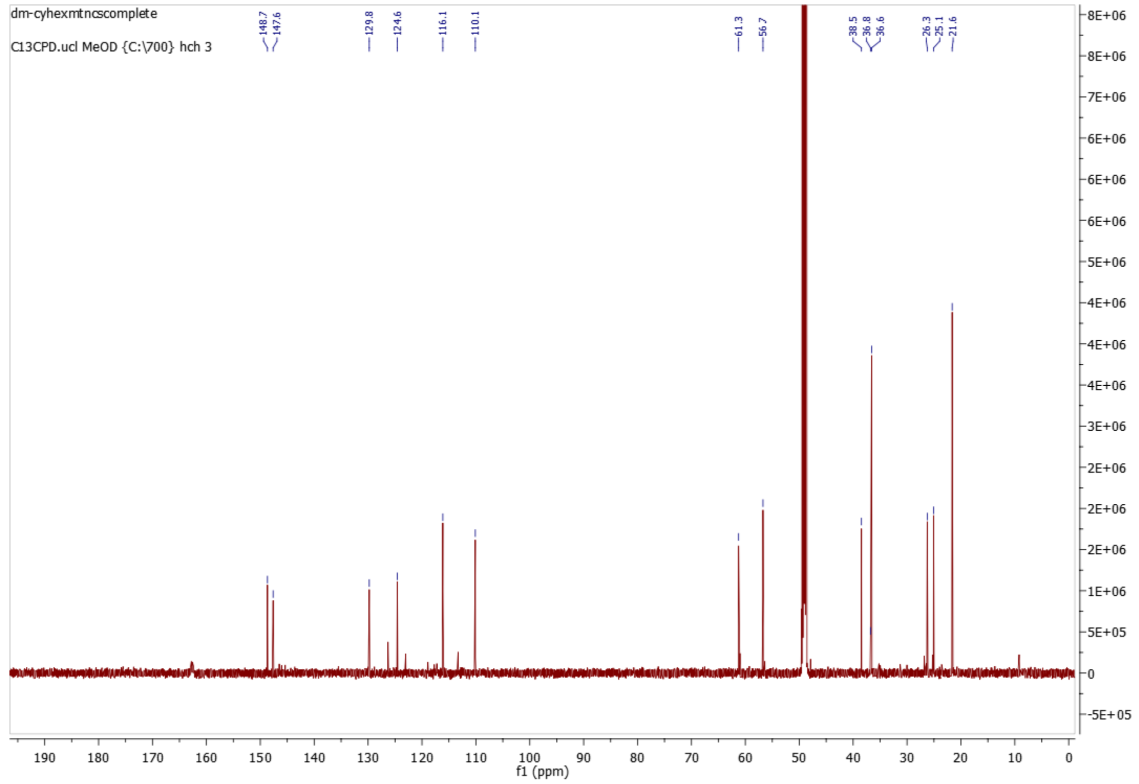
dm-cyhexmtncscomplete
C13CPD.ucl MeOD {C:\700} hch 3
f1 (ppm)

Four-Enzymes Cascade to 6'-methoxy-3',4'-dihydro-2'H-spiro[cyclohexane-1,1'-isoquinolin]-7'-ol 6-OMe-9

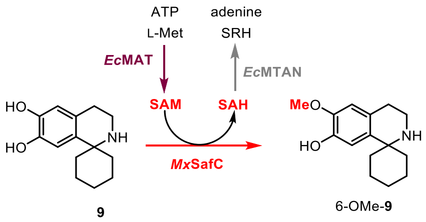

Compound **9** (TFA salt, 33 mg, 0.1 mmol), ATP (66 mg, 0.12 mmol), and L-methionine (18 mg, 0.12 mmol) were combined in HEPES buffer (50 mM pH 7.5). The methylation step was initiated upon addition of 10% v/v of *Ec*MAT lysate, 2% v/v of *Ec*MTAN lysate, and 10% v/v of *Mx*SafC lysate in a total volume of 10 mL containing 20 mM $MgCl_2$ and 200 mM KCl. The reaction was incubated overnight at 37 °C and 180 rpm. After this time an aliquot was taken to calculate the yield by HPLC against the product standard (see calibration curve below). The reaction was centrifuged, the supernatant was basified with $NaHCO_3$ and extracted with EtOAc (3 x 10 mL). The organic fractions were collected, evaporated under vacuum and the residue purified using preparative HPLC (method B). Fractions containing the desired product were freeze-dried to give 6-OMe-**9** as white powder (TFA salt, 13.8 mg, 56% yield, 94% yield by HPLC). An impurity (10%) was also identified by NMR corresponding to the regioisomeric product methylated at *O*-7. ^{1}H NMR (700 MHz; CD_3OD) δ = 6.81 (s, 1H, 8-H), 6.73 (s, 1H, 5-H), 3.84 (s, 3H, OMe), 3.44 (t, *J* = 6.4 Hz, 2H, 3'-H), 3.04 (t, *J* = 6.4 Hz, 2H, 4'-H), 2.08-2.02 (m, 4H, 2-HH and 6-HH), 1.89 – 1.79 (m, 3H), 1.65-1.55 (m, 2H), 1.51 (tt, *J* = 12.7, 3.7 Hz, 1H, 4-H*H*); ^{13}C NMR (151 MHz; CD_3OD) δ = 148.9, 147.1, 131.1, 122.9, 113.2, 112.5, 61.1, 56.4, 38.6, 36.6, 26.4, 25.2, 21.6; HRMS (ES+) found $[M+H]^+$ 248.1645; $C_{15}H_{22}NO_2$ requires 248.1645.

HPLC Method A1

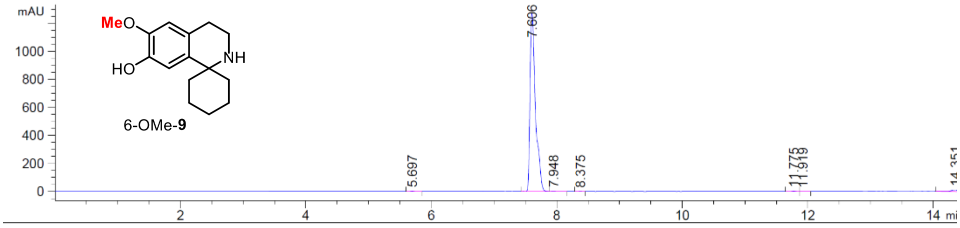

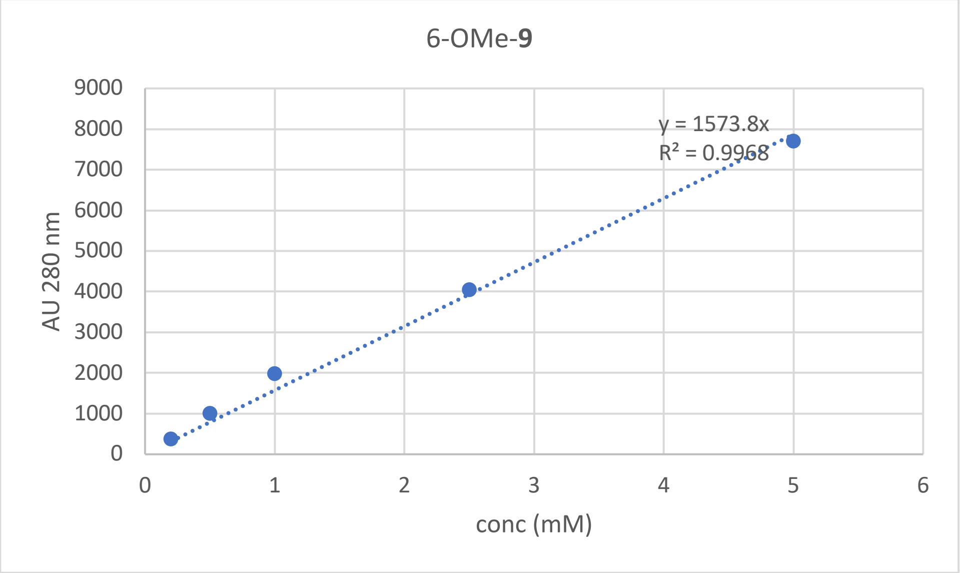

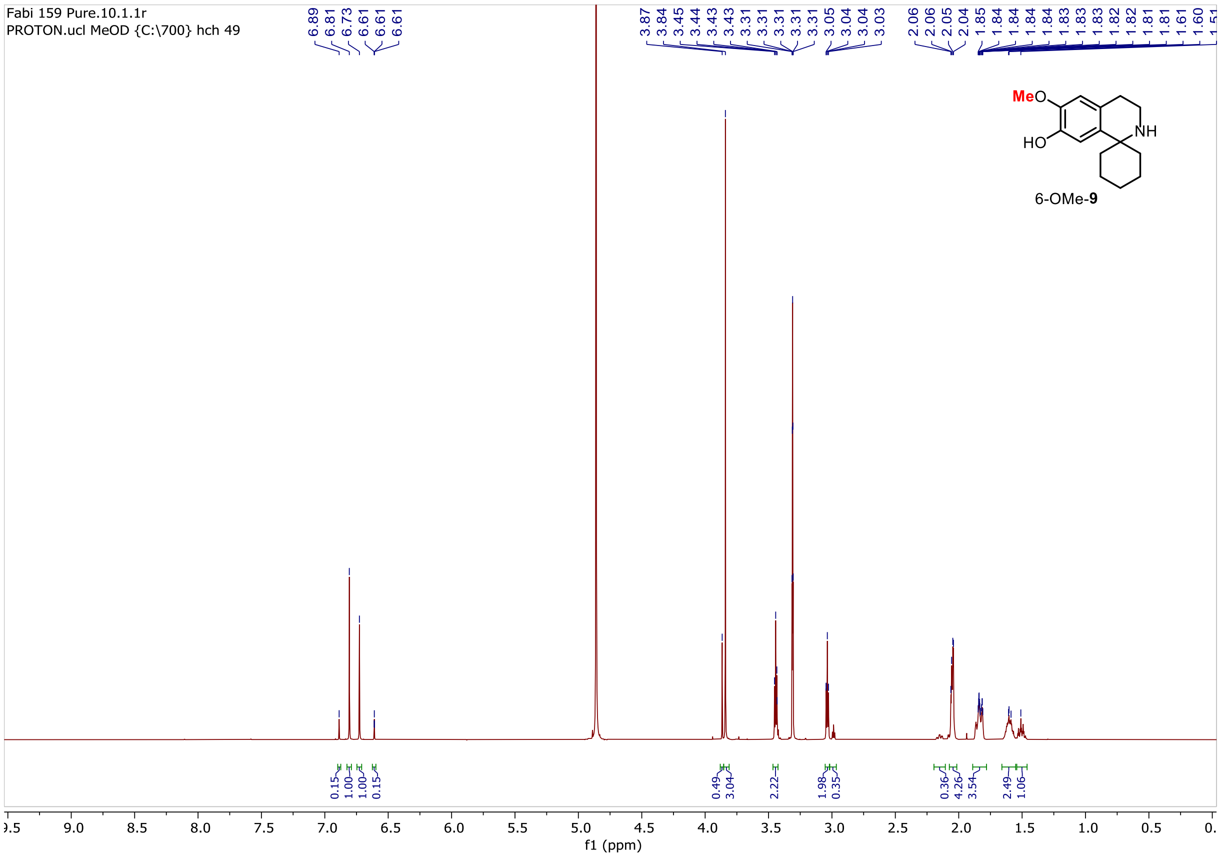
Fabi 159 Pure.10.1.1r
PROTON.ucl MeOD {C:\700} hch 49
6.89 6.81 6.73 6.61 6.61 6.61
3.87 3.84 3.45 3.44 3.43 3.43 3.31 3.31 3.31 3.31 3.31 3.05 3.04 3.04 3.03
2.06 2.06 2.05 2.04 1.85 1.84 1.84 1.84 1.84 1.83 1.83 1.83 1.82 1.81 1.81 1.61 1.60
MeO
HO
NH
6-OMe-9
0.15 1.00 1.00 0.15
0.49 3.04
2.22
1.98 0.35
0.36 4.26 3.54
2.49 1.06
f1 (ppm)

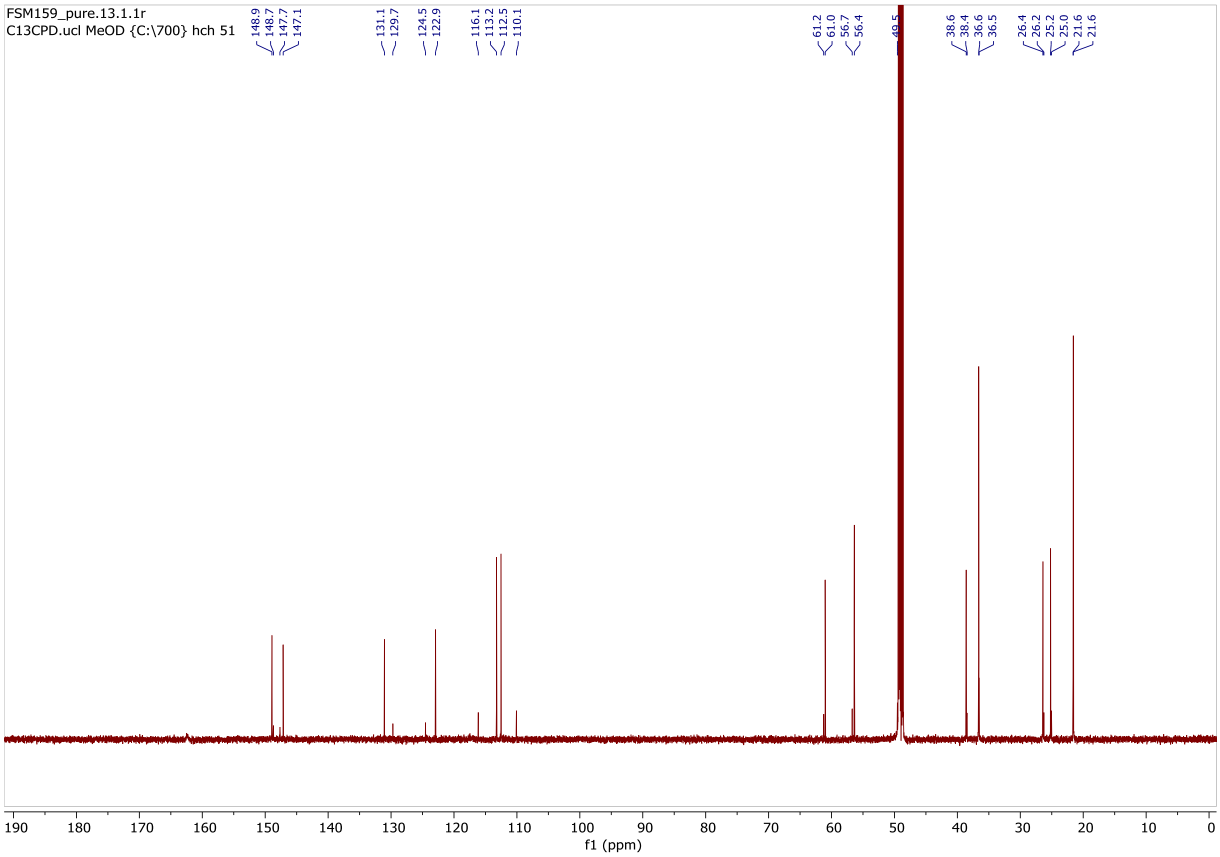
FSM159_pure.13.1.1r
C13CPD.ucl MeOD {C:\700} hch 51
148.9 148.7 147.7 147.1
131.1 129.7
124.5 122.9
116.1 113.2 112.5 110.1
61.2 61.0 56.7 56.4
49.5
38.6 38.4 36.6 36.5
26.4 26.2 25.2 25.0 21.6 21.6
f1 (ppm)

Synthesis of 7'-methoxy-3',4'-dihydro-2'H-spiro[cyclopentane-1,1'-isoquinolin]-6'-ol 7-OMe-10

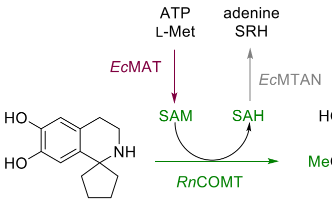

10 7-OMe-**10**

Compound **10** (TFA salt, 16 mg, 0.05 mmol), ATP (55 mg, 0.1 mmol), and L-methionine (15 mg, 0.1 mmol) were combined in HEPES buffer 50 mM pH 7.5. The methylation step was initiated upon addition of 10% v/v of *Ec*MAT lysate, 2% v/v of *Ec*MTAN lysate, and 10% v/v of *Mx*SafC lysate in a total volume of 10 mL containing 20 mM $MgCl_2$ and 200 mM KCl. The reaction was incubated overnight at 37 °C and 180 rpm. After this time an aliquot was taken to calculate the yield by HPLC against the product standard (see calibration curve below). The reaction was centrifuged, the supernatant was basified with $NaHCO_3$ and extracted with EtOAc (3 x 10 mL). The organic fractions were collected, evaporated under vacuum and the residue purified using preparative HPLC (method B). Fractions containing the desired product were freeze-dried to give 7-OMe-**10** as white powder (TFA salt, 7.4 mg, 45% yield, 90% yield by HPLC). An impurity (10%) were also identified by NMR corresponding to the regioisomeric product methylated at *O*-6. Only signal for the major isomer are shown. ^{1}H NMR (700 MHz, MeOD) δ 6.74 (s, 1H, 8'-H), 6.60 (s, 1H, 5'-H), 3.84 (s, 3H, OMe), 3.43 (t, *J* = 6.4 Hz, 2H, 3'-H), 2.97 (t, *J* = 6.4 Hz, 2H, 4'-H), 2.26 (m, 2H), 2.23 – 2.16 (m, 2H), 2.06 (m, 2H), 1.96 (m, 2H); ^{13}C NMR (176 MHz, MeOD) δ 148.8, 147.4, 129.1, 124.7, 115.8, 109.4, 68.9, 56.5, 41.8 x 2, 39.9, 25.7 x 3; HRMS (ES+) found [M+H]$^+$ 234.1486; $C_{14}H_{20}NO_2$ requires 234.1489.

HPLC Method A1

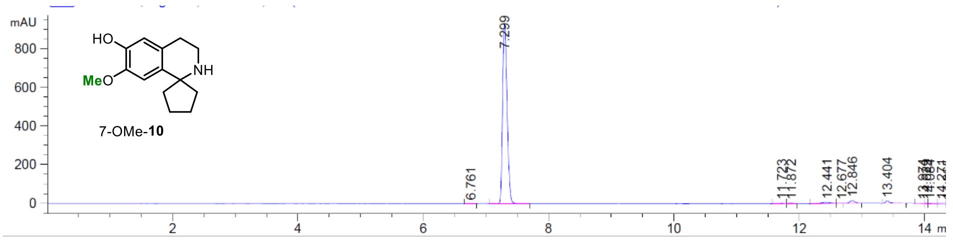

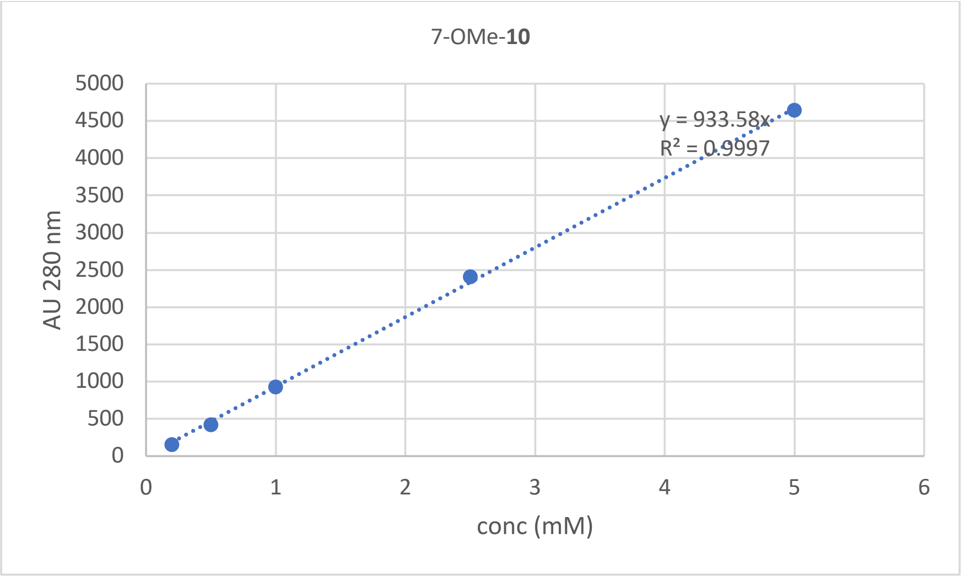

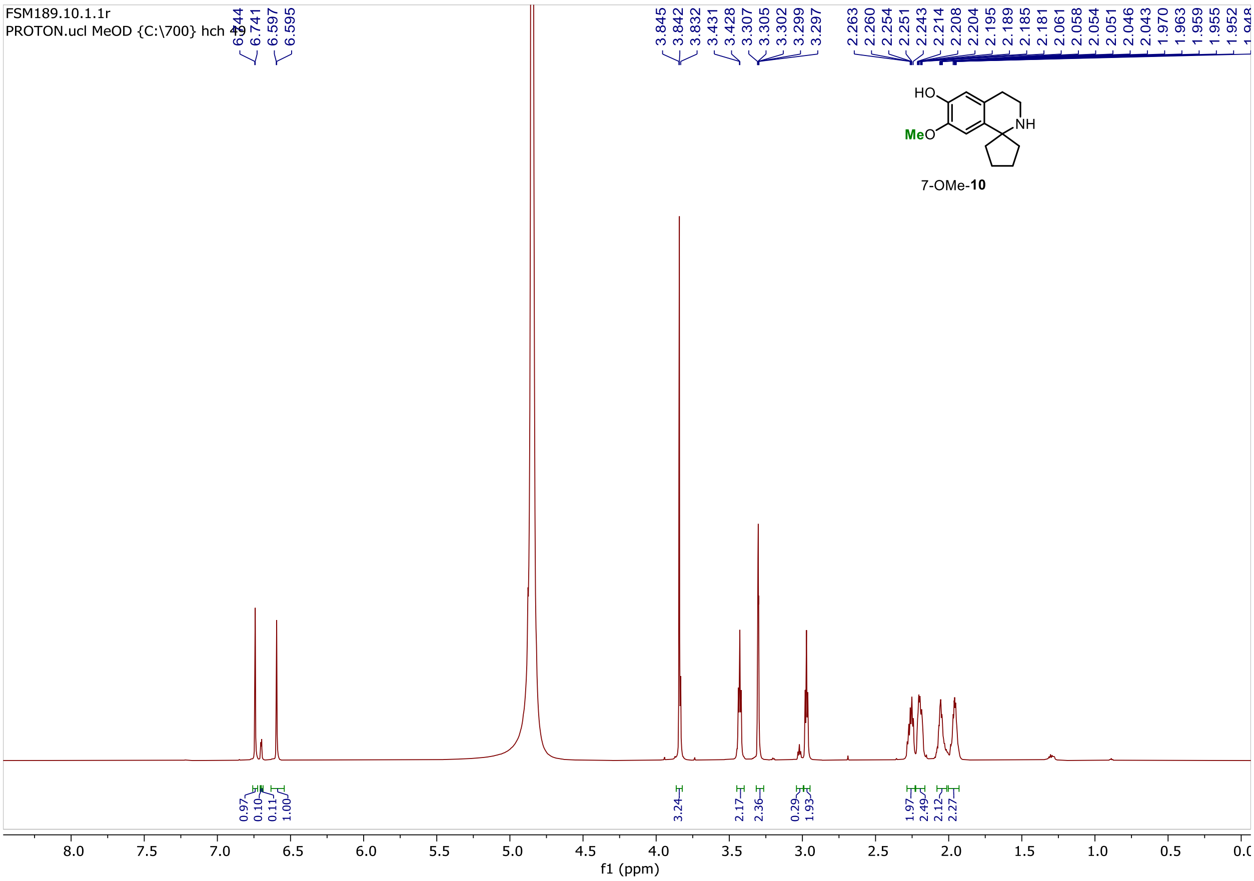
FSM189.10.1.1r
PROTON.ucl MeOD {C:\700} hch 49
6.744
6.741
6.597
6.595
3.845
3.842
3.832
3.431
3.428
3.307
3.305
3.302
3.299
3.297
2.263
2.260
2.254
2.251
2.243
2.214
2.208
2.204
2.195
2.189
2.185
2.181
2.061
2.058
2.054
2.051
2.046
2.043
1.970
1.963
1.959
1.955
1.952
HO
MeO
NH
7-OMe-10
0.97
0.10
0.11
1.00
3.24
2.17
2.36
0.29
1.93
1.97
2.49
2.12
2.27
f1 (ppm)

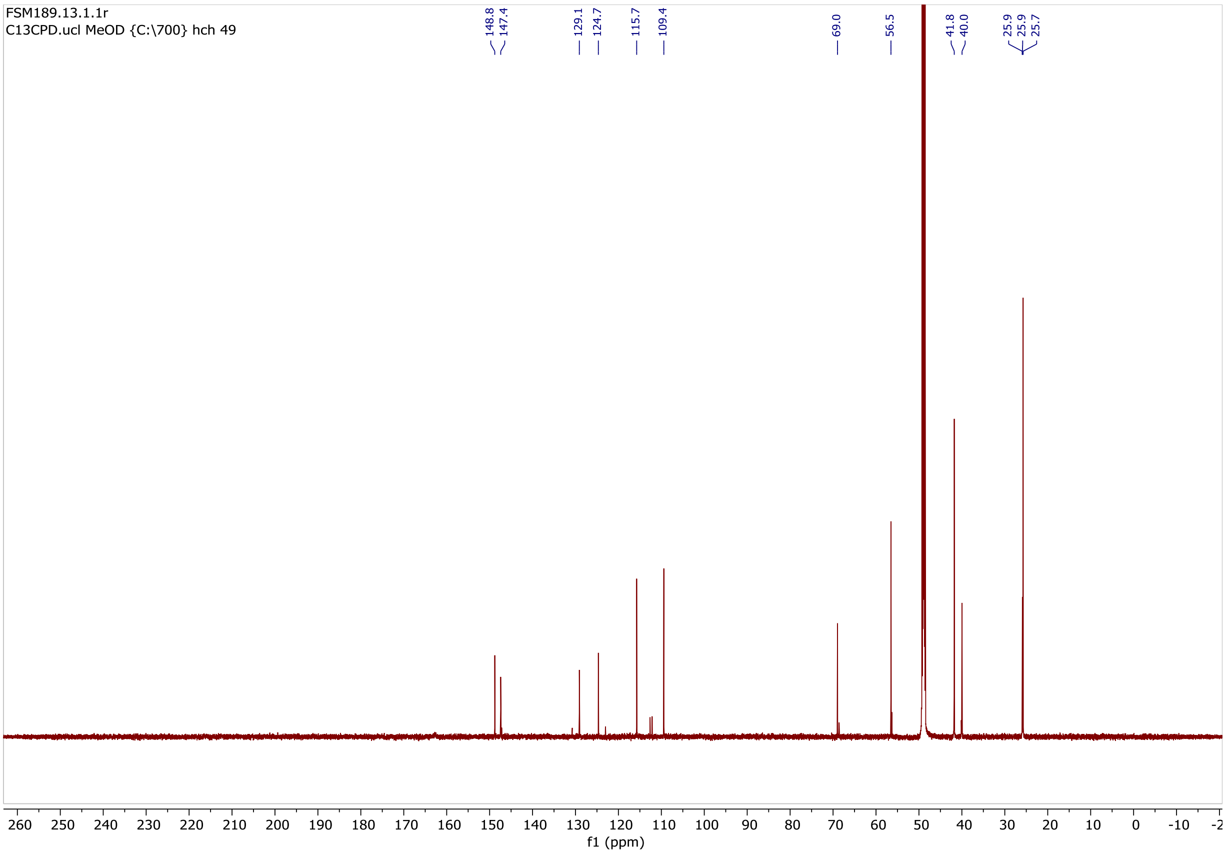
FSM189.13.1.1r
C13CPD.ucl MeOD {C:\700} hch 49
148.8
147.4
129.1
124.7
115.7
109.4
69.0
56.5
41.8
40.0
25.9
25.9
25.7
f1 (ppm)

Synthesis of 6'-methoxy-3',4'-dihydro-2'H-spiro[cyclopentane-1,1'-isoquinolin]-7'-ol 6-OMe-10

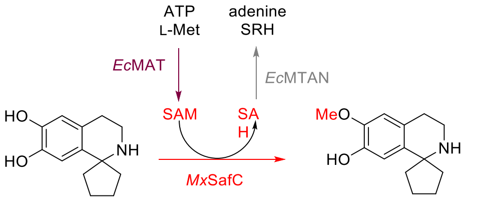

10 6-OMe-**10**

Compound **10** (TFA salt, 16 mg, 0.05 mmol), ATP (55 mg, 0.1 mmol), and L-methionine (15 mg, 0.1 mmol) were combined in HEPES buffer 50 mM pH 7.5. The methylation step was initiated upon addition of 10% v/v of *Ec*MAT lysate, 2% v/v of *Ec*MTAN lysate, and 10% v/v of *Mx*SafC lysate in a total volume of 10 mL containing 20 mM $MgCl_2$ and 200 mM KCl. The reaction was incubated overnight at 37 °C and 180 rpm. After this time an aliquot was taken to calculate the yield by HPLC against the product standard (see calibration curve below). The reaction was centrifuged, the supernatant was basified with $NaHCO_3$ and extracted with EtOAc (3 x 10 mL). The organic fractions were collected, evaporated under vacuum and the residue purified using preparative HPLC (method B). Fractions containing the desired product were freeze-dried to give 6-OMe-**10** as white powder (TFA salt, 4.9 mg, 30% yield, 89% yield by HPLC). An impurity (10%) was also identified by NMR corresponding to the regioisomeric product methylated at *O*-7. Only signals for the major isomer are shown. ^{1}H NMR (700 MHz, MeOD) δ 6.71 (s, 1H, 8'-H), 6.70 (s, 1H, 5'-H), 3.84 (s, 3H, OMe), 3.45 (t, *J* = 6.4 Hz, 2H, 3'-H), 3.02 (t, *J* = 6.4 Hz, 2H, 4'-H), 2.20-2.17 (m, 4H), 2.03-2.01 (m, 2H), 1.96-1.93 (m, 2H); ^{13}C NMR (176 MHz, MeOD) δ 148.9, 147.4, 130.8, 123.0, 112.7, 112.1, 68.7, 56.3, 42.0 x 2, 40.3, 26.2 x 3; HRMS (ES+) found $[M+H]^+$ 234.1485; $C_{14}H_{20}NO_2$ requires 234.1489.

HPLC Method A1 (achiral)

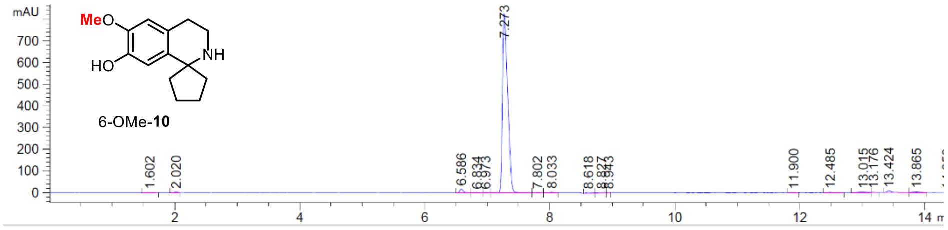

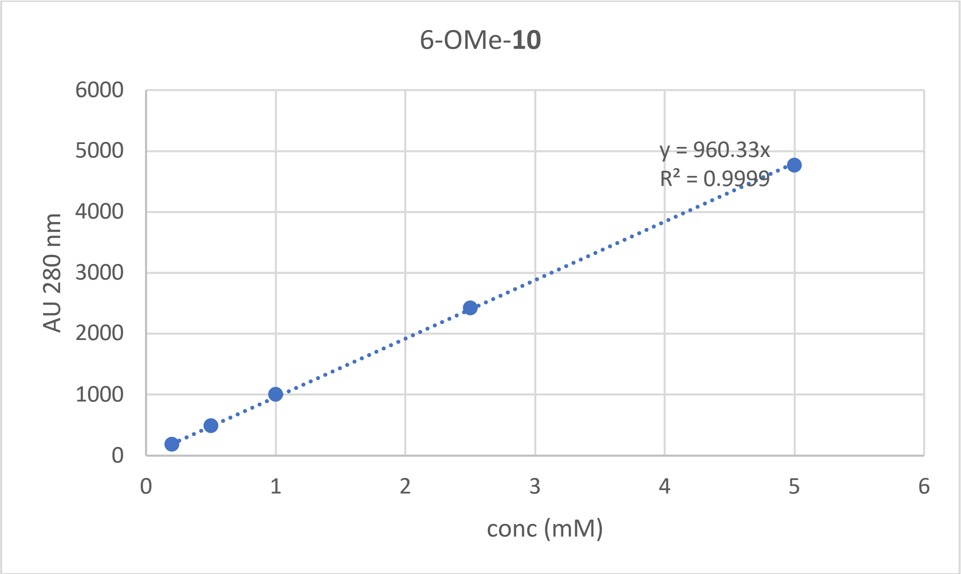

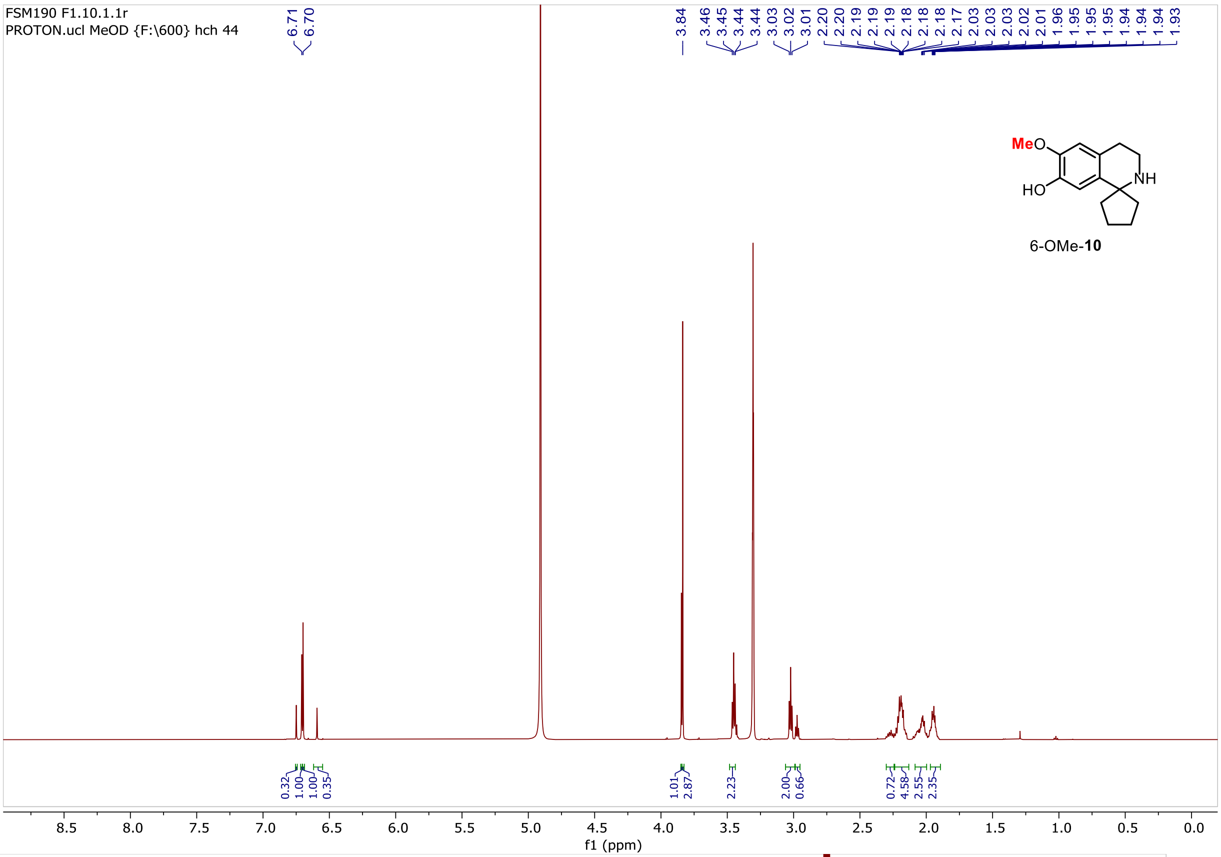
FSM190 F1.10.1.1r
PROTON.ucl MeOD {F:\600} hch 44
6.71
6.70
3.84
3.46
3.45
3.44
3.44
3.03
3.02
3.01
2.20
2.20
2.19
2.19
2.19
2.18
2.18
2.18
2.17
2.03
2.03
2.03
2.02
2.01
1.96
1.95
1.95
1.94
1.94
1.94
1.93
MeO
HO
NH
6-OMe-10
0.32
1.00
1.00
0.35
1.01
2.87
2.23
2.00
0.66
0.72
4.58
2.55
2.35
8.5
8.0
7.5
7.0
6.5
6.0
5.5
5.0
4.5
4.0
3.5
3.0
2.5
2.0
1.5
1.0
0.5
0.0
f1 (ppm)

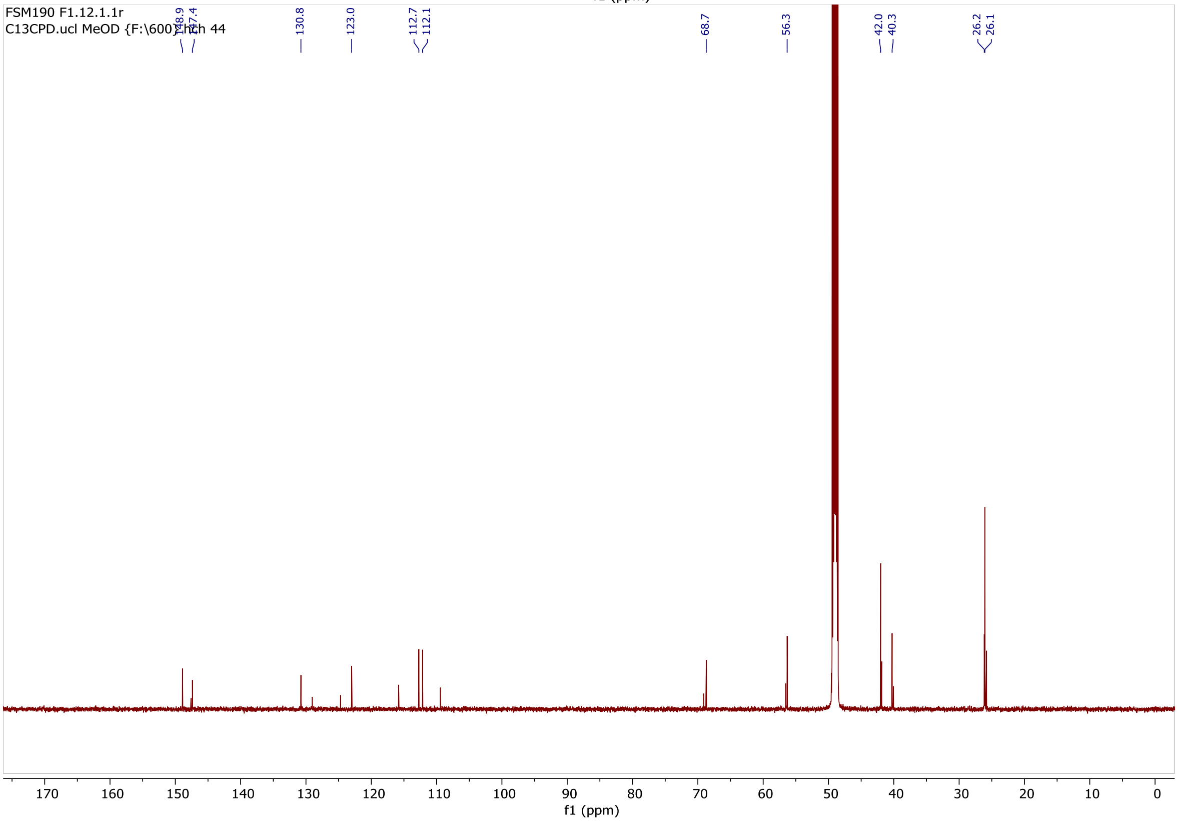
FSM190 F1.12.1.1r
C13CPD.ucl MeOD {F:\600} hch 44
148.9
147.4
130.8
123.0
112.7
112.1
68.7
56.3
42.0
40.3
26.2
26.1
170
160
150
140
130
120
110
100
90
80
70
60
50
40
30
20
10
0
f1 (ppm)

Four-Enzymes Cascade to (*S*)-6-methoxy-1-phenethyl-1,2,3,4-tetrahydroisoquinolin-7-ol (*S*)-6-OMe-11

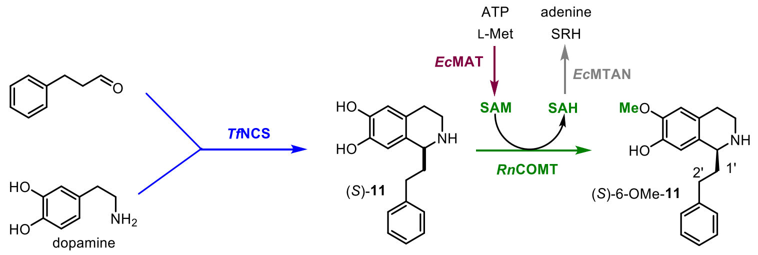

Dopamine hydrochloride (19 mg, 0.1 mmol), 3-phenylpropanal (53.1 µL, 0.4 mmol) dissolved in 1 mL of acetonitrile, sodium ascorbate (29.7 mg, 0.15 mmol), and *Tf*NCS (5 mg) were combined with HEPES (50 mM pH 7.5) mM to a total volume of 10 mL. The reaction was stirred at 37 °C with 200 rpm shaking until completion (3 h). After completion, the methylation step was initiated in the same pot and all the components were added to have a final concentration of 10 mM ATP (110 mg, 0.2 mmol), 10 mM of L-methionine (7.5 mg, 0.05 mmol), 10% v/v of *Ec*MAT lysate, 2% v/v of *Ec*MTAN lysate, and 10% v/v of *Rn*COMT lysate in a total volume of 20 mL containing 20 mM $MgCl_2$ and 200 mM KCl. The reaction was started upon enzymes addition and incubated overnight at 37 °C at 200 rpm. After this time an aliquot was taken to calculate the yield by HPLC against the product standard (see calibration curve below). The reaction was quenched with 0.5 M HCl (2 ml), centrifuged and the supernatant was purified using preparative HPLC (method B). Fractions containing the product were concentrated under vacuum and re-dissolved in 0.1 M aqueous HCl, before lyophilisation to give the HCl salt of (*S*)-6-OMe-**11** as a white powder (8.2 mg, 33%, 40% yield by HPLC). Impurities (15%) were also identified by NMR corresponding to the regioisomeric product methylated at *O*-7. ^{1}H NMR (700 MHz; D_2O with MeOH standard) δ = 7.37 (t, *J* = 7.6 H, 2H, *m*-Ph), 7.31-7.26 (m, 3H, *o*-Ph + *p*-Ph), 6.88 (s, 1H, 8-H), 6.74 (s, 1H, 5-H), 4.50 (dd, *J* = 7.8, 4.9 Hz, 1H, 1-H), 3.84 (s, 3H, OMe), 3.60 (dt, *J* = 13.0, 6.0 Hz, 1h, 3-*H*H), 3.39 (ddd, *J* = 13.0, 7.5, 6.0 Hz, 1H, 3-H*H*), 3.07 (dt, *J* = 17.0, 6.0 Hz, 1H, 4-*H*H), 3.00 (dt, *J* = 17.0, 6.0 Hz, 1H, 4-H*H*), 2.84 - 2.74 (m, 2H, 2'-H), 2.38 - 2.30 (m, 1H, 1'-*H*H), 2.28 - 2.19 (m, 1H, 1'-H*H*); ^{13}C NMR (151 MHz; D_2O with MeOH standard) δ = 148.3, 144.8, 141.6, 129.8, 129.4, 127.5, 125.0, 124.8, 114.0, 113.3, 56.8, 55.5, 40.3, 36.0, 31.7, 25.2; *m/z* HRMS (ESI+) found $[M+H]^+$ 284.1644; $C_{18}H_{22}NO_2$ requires 284.1645. $[\alpha]_D^{24}$ = +6.4 (c 0.10, H_2O).

HPLC Method A1 (achiral)

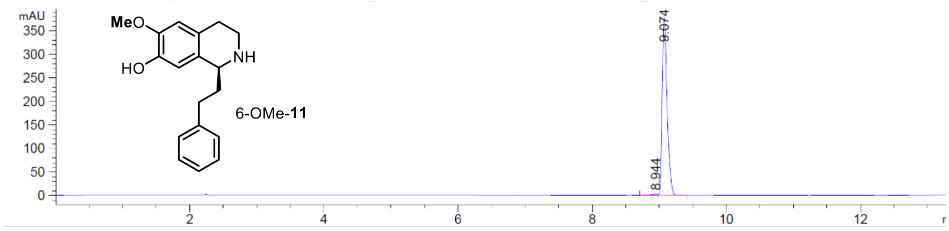

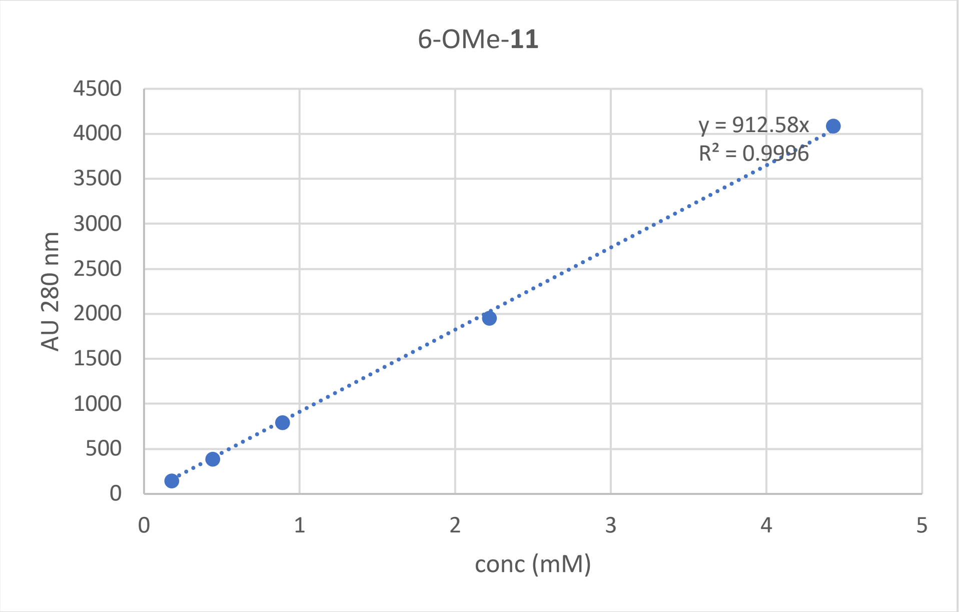

HPLC Method C3 (chiral) (*S*)-**11**

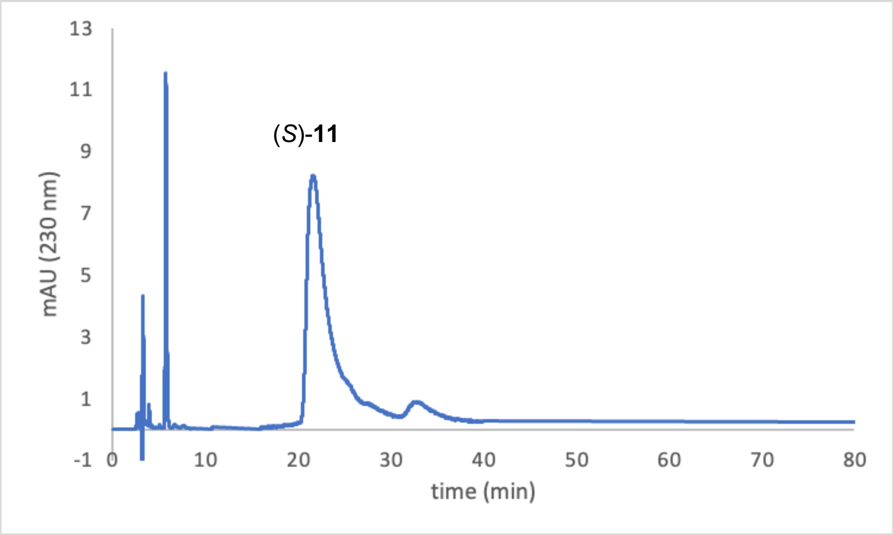

HPLC Method C3 (chiral) rac-**11**

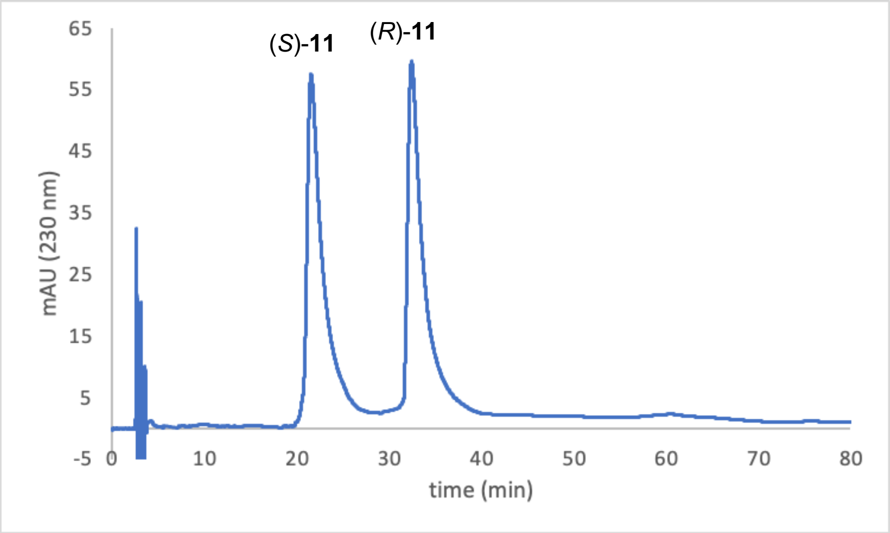

HPLC Method C1 (chiral) (*S*)-6-OMe-**11**

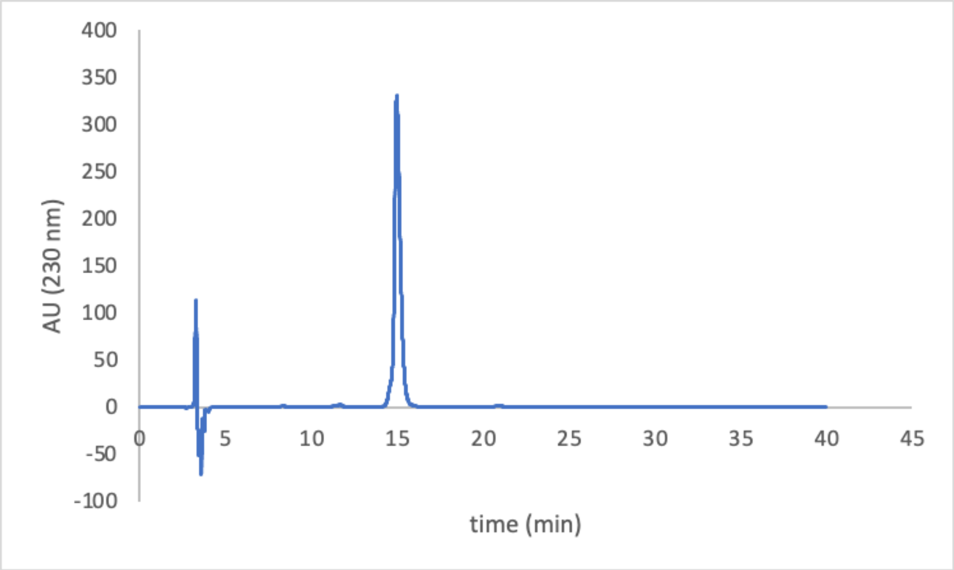

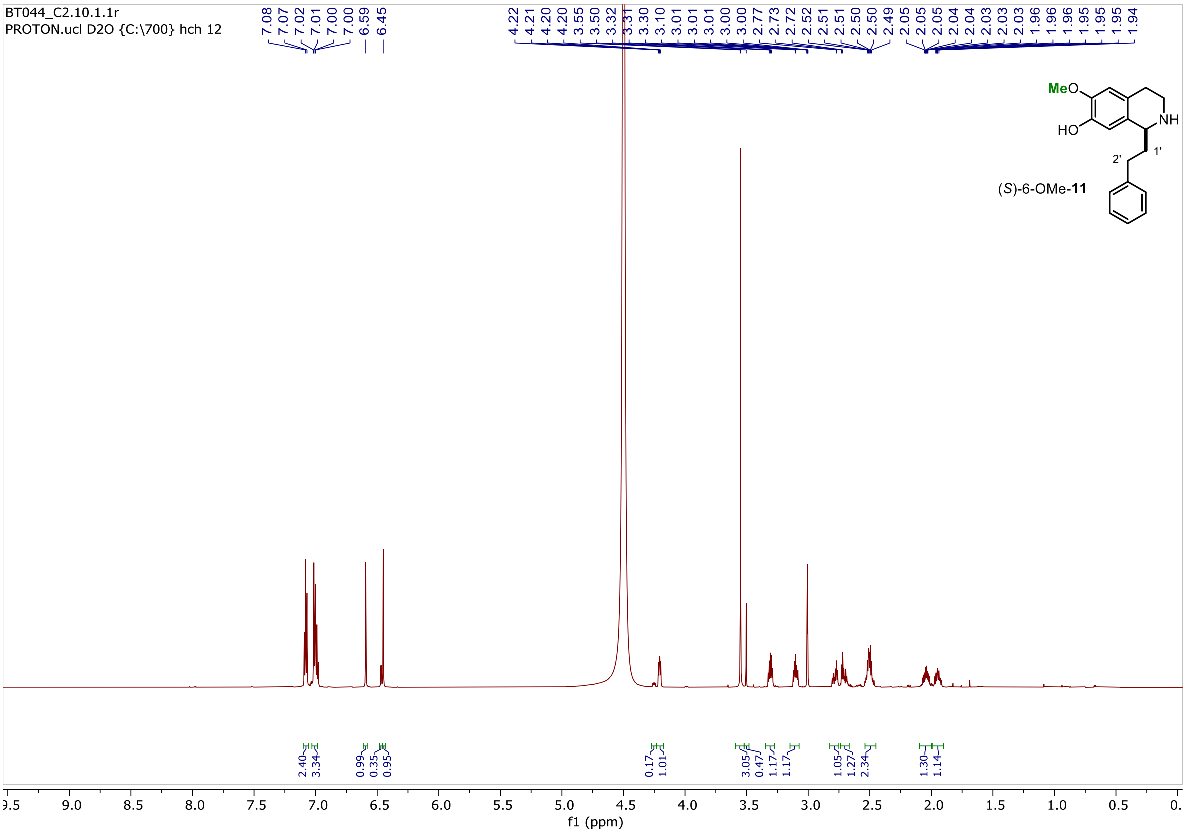
BT044_C2.10.1.1r
PROTON.ucl D2O {C:\700} hch 12
7.08 7.07 7.02 7.01 7.00 7.00 6.59 6.45
4.22 4.21 4.20 4.20 3.55 3.50 3.32 3.31 3.30 3.10 3.01 3.01 3.01 3.00 3.00 2.77 2.73 2.72 2.52 2.51 2.51 2.50 2.50 2.49 2.05 2.05 2.05 2.04 2.04 2.03 2.03 1.96 1.96 1.96 1.95 1.95 1.94
MeO
HO
NH
2'
1'
(S)-6-OMe-11
2.40 3.34 0.99 0.35 0.95 0.17 1.01 3.05 0.47 1.17 1.17 1.05 1.27 2.34 1.30 1.14
9.5 9.0 8.5 8.0 7.5 7.0 6.5 6.0 5.5 5.0 4.5 4.0 3.5 3.0 2.5 2.0 1.5 1.0 0.5 0
f1 (ppm)

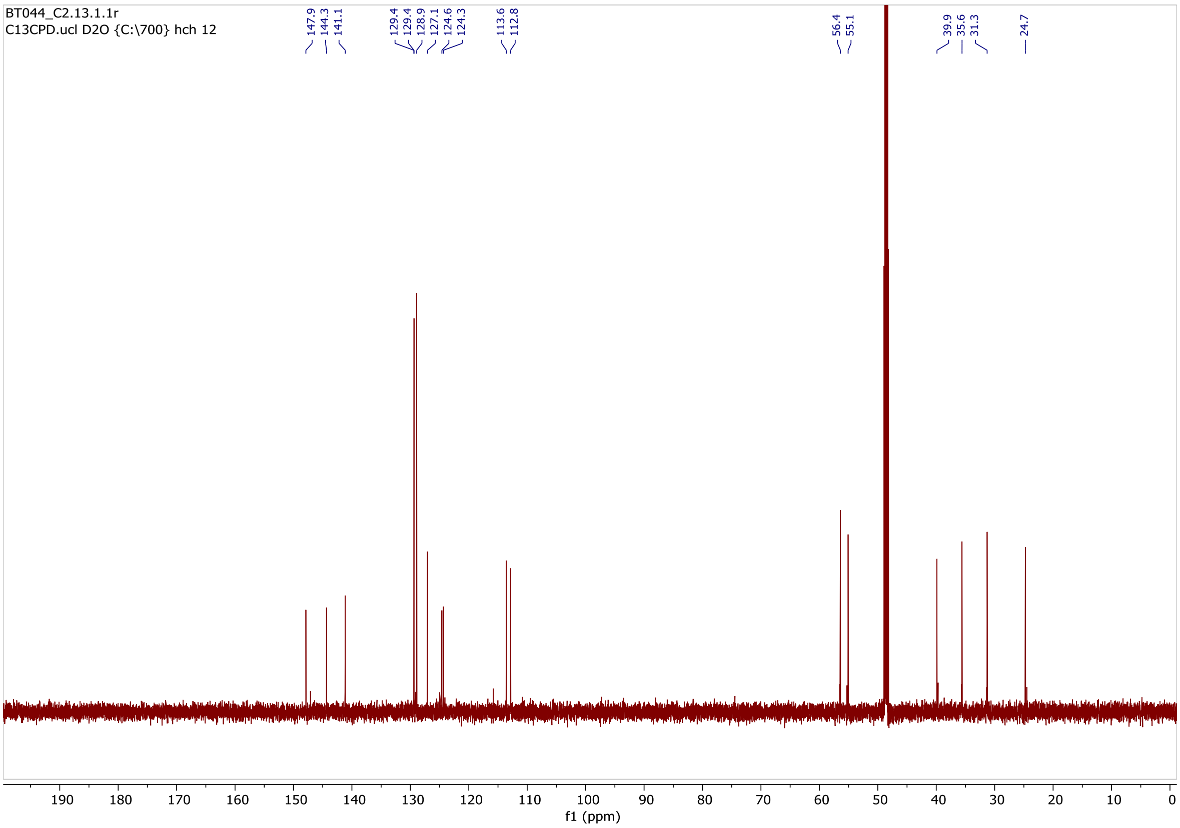
BT044_C2.13.1.1r
C13CPD.ucl D2O {C:\700} hch 12
147.9 144.3 141.1 129.4 129.4 128.9 127.1 124.6 124.3 113.6 112.8 56.4 55.1 39.9 35.6 31.3 24.7
190 180 170 160 150 140 130 120 110 100 90 80 70 60 50 40 30 20 10 0
f1 (ppm)

4. Analytical HPLC chromatograms

(a)

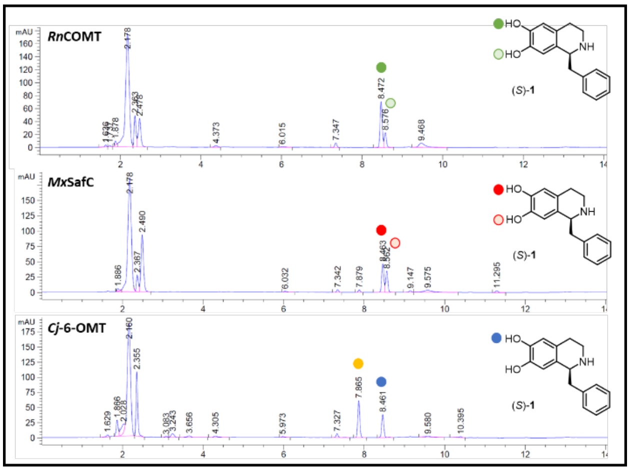

(b)

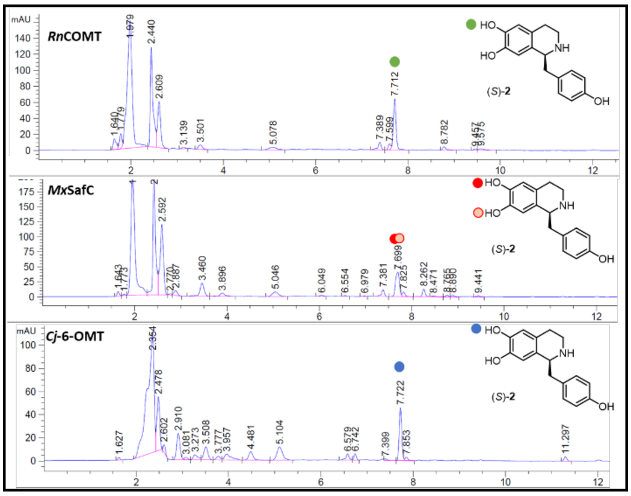

(c)

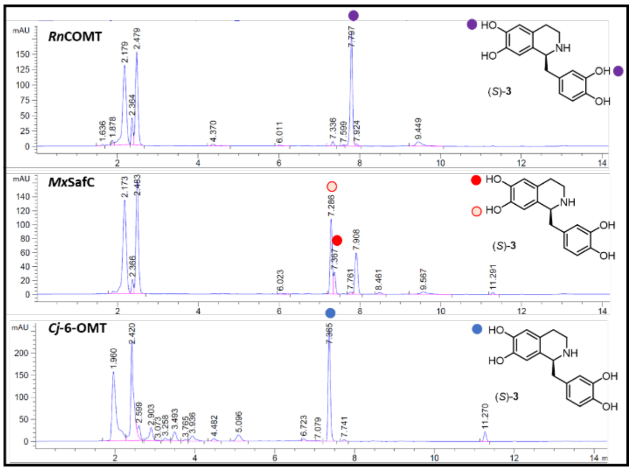

(d)

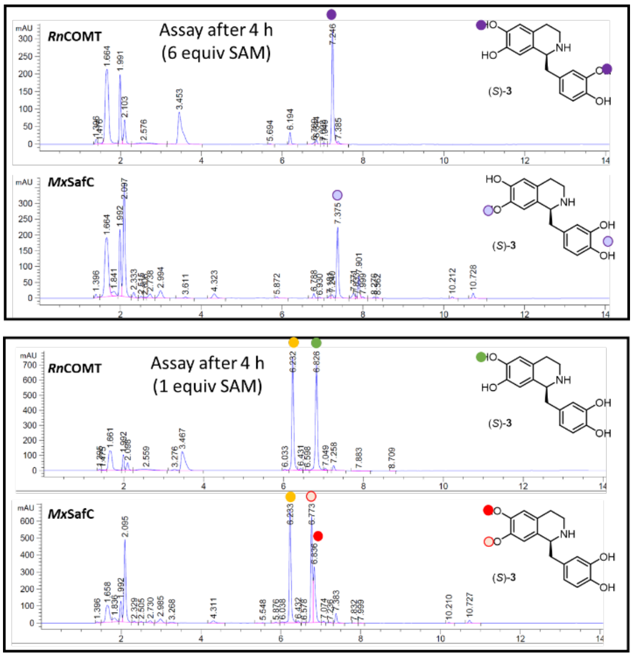

(e)

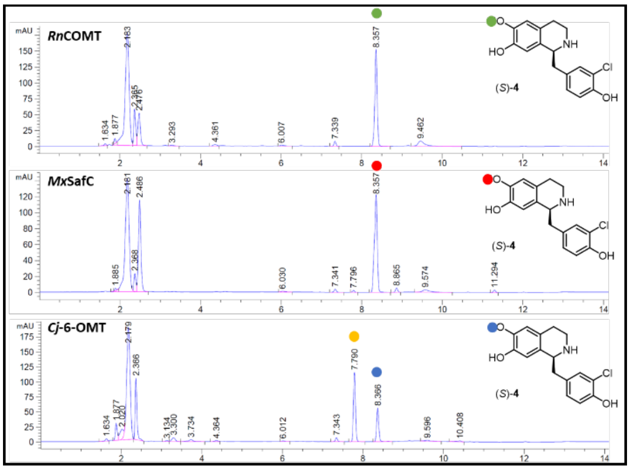
RnCOMT
MxSafC
Cj-6-OMT
(S)-4
HO
NH
Cl
OH
mAU

(f)

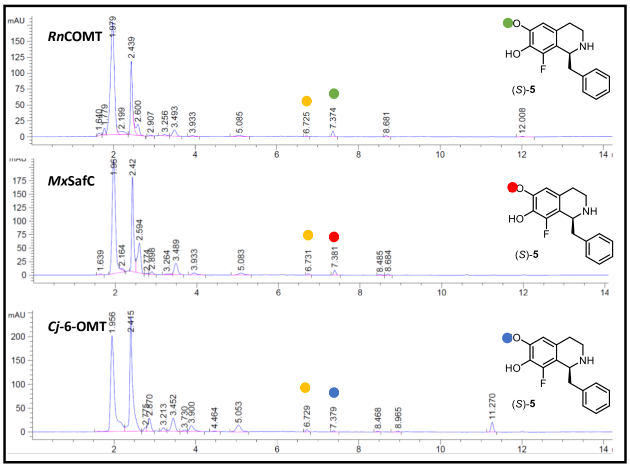
RnCOMT
MxSafC
Cj-6-OMT
(S)-5
HO
NH
F
mAU

(g)

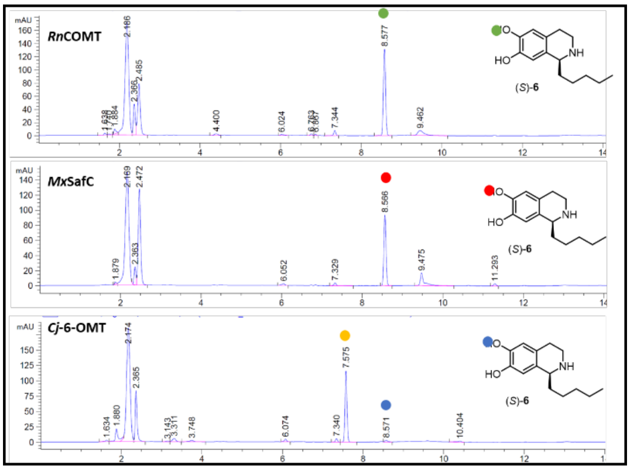
RnCOMT
MxSafC
Cj-6-OMT
(S)-6
HO
NH
mAU

(h)

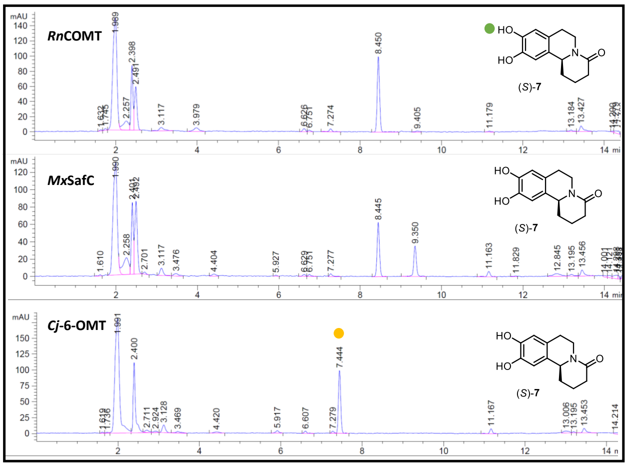
RnCOMT
MxSafC
Cj-6-OMT
(S)-7
HO
N
mAU

(i)

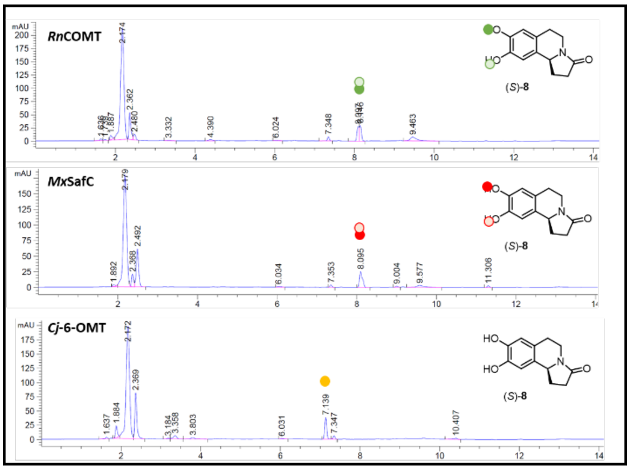
RnCOMT
MxSafC
Cj-6-OMT
(S)-8
HO
N
mAU

(j)

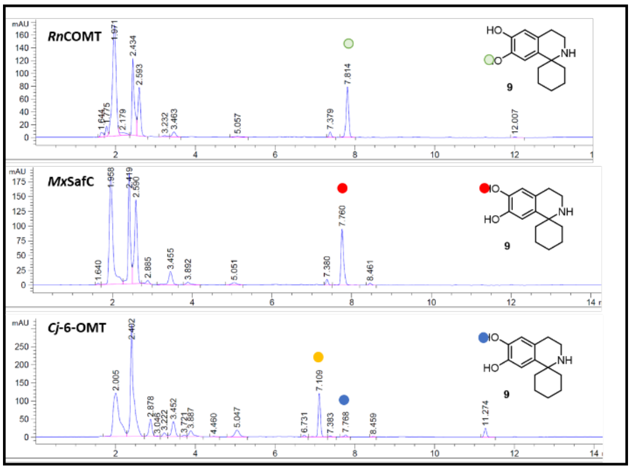
RnCOMT
MxSafC
Cj-6-OMT
9
HO
NH
mAU

(k)

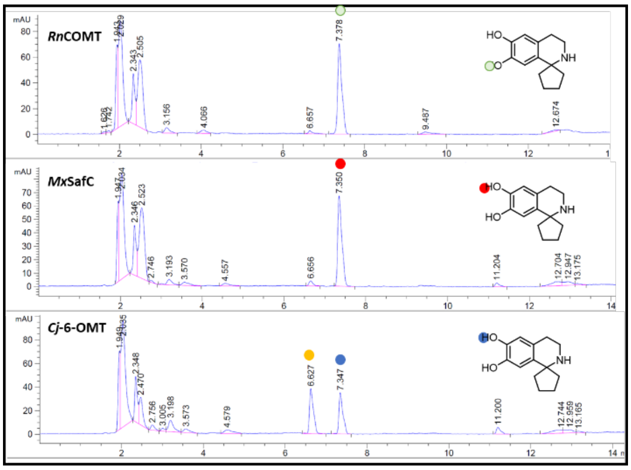

(l)

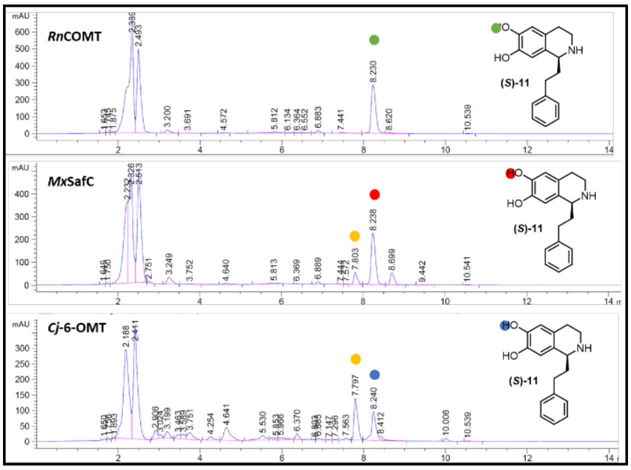

Figure S5. Representative HPLC chromatograms from the single step methylation reaction (preliminary screening) using SAM. For each methyltrasferase: yellow dots correspond to starting material, full dots correspond to 6-OMe product, light dots correspond to 7-OMe products, purple dots correspond respectively to the dimethoxy products.

5. Docking

Docking was performed with Chimera UCSF using the AutoDock Vina plugin,[11,12] (docking box parameters and settings: energy-range (kcal/mol) 3, exhaustiveness 8, number of modes 5). Binding modes relevant to the explanation of enzyme reactivity were selected. Images generated using Chimera.[12] *Rn*COMT (PDB ID: 1VID) and *Mx*SafC (PDB ID: 5LOG).

Docking analysis of derivative (*S*)-6-OMe-**3** for the second methylation with *Rn*COMT or *Mx*SafC

This showed that the aromatic side chain can occupy the active site, with the catechol moiety coordinated with the magnesium ion (Figure S6). *Rn*COMT exhibited high regioselectivity for the second methylation at 3'-OH which is located towards the cofactor and in proximity to the catalytic lysine (Figure S6a). The THIQ heterocyclic ring interacts with W143 (π-π stacking) on the hydrophobic wall, directing the methylation to the 3'-OH. For *Mx*SafC (Figure S6b) the interactions of the THIQ moiety with the outer region of the active site, is consistent with the favoured methylation at 4'-OH which is kept towards the catalytic lysine, K145 and near the cofactor.

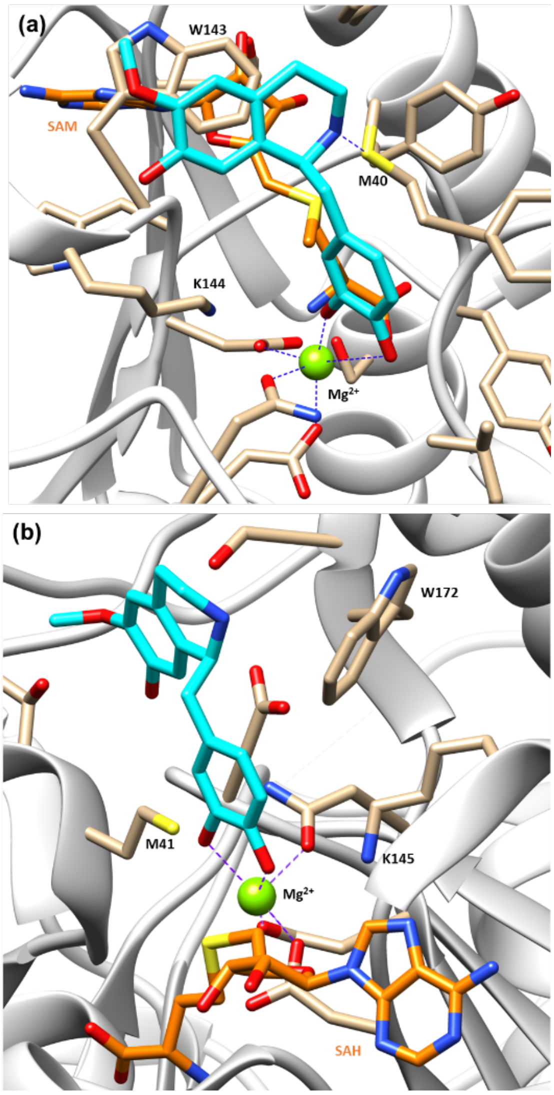

Figure S6. Computational docking of (*S*)-6-OMe-**3** (blue) into the active site **(a)** *Rn*COMT (PDB ID: 1VID) containing SAM (orange) and **(b)** *Mx*SafC (PDB ID: 5LOG) containing SAH (orange). The magnesium ion is represented in green.

6. References

[1] J. Vidgren, L. A. Svensson, A. Liljas, *Nature* **1994**, *368*, 354–358.

[2] J. Siegrist, J. Netzer, S. Mordhorst, L. Karst, S. Gerhardt, O. Einsle, M. Richter, J. N. Andexer, *FEBS Lett.* **2017**, *591*, 312–321.

[3] J. Siegrist, S. Aschwanden, S. Mordhorst, L. Thöny-Meyer, M. Richter, J. N. Andexer, *ChemBioChem* **2015**, *16*, 2576–2579.

[4] Y. Wang, N. Tappertzhofen, D. Méndez-Sánchez, M. Bawn, B. Lyu, J. M. Ward, H. C. Hailes, *Angew. Chem. Int. Ed.* **2019**, *58*, 10120–10125.

[5] D. S. Bhakuni, A. N. Singh, S. Jain, *Tetrahedron* **1981**, *37*, 2651–2655.

[6] T. Pesnot, M. C. Gershater, J. M. Ward, H. C. Hailes, *Chemm. Commun*, **2011**, *47*, 3242-3244.

[7] D. H. R. Barton, A. A. L. Gunatilaka, R. M. Letcher, A. M. F. T. Lobo, D. A. Widdowson, *J. Chem. Soc., Perkin Trans. I* **1973**, 874–880.

[8] N. Saidi, H. Morita, M. Litaudon, M. R. Mukhtar, K. Awang, A. Hamid, A. Hadi, *Indo. J. Chem.* **2011**, *11*, 59–66.

[9] K. C. Rice, A. Brossi, *J. Org. Chem.* **1980**, *45*, 592–601.

[10] M. J. Vanden Eynden, K. Kunchithapatham, J. P. Stambuli, *J. Org. Chem.* **2010**, *75*, 8542–8549.

[11] O. Trott, A. J. Olson, *J. Comput. Chem.* **2010**, *31*, 455–461.

[12] E. F. Pettersen, T. D. Goddard, C. C. Huang, G. S. Couch, D. M. Greenblatt, E. C. Meng, T. E. Ferrin, *J. Comput. Chem.* **2004**, *25*, 1605–1612.
